# Correction: Strategic selection of MDM2 inhibitors enhances the efficacy of FAK inhibition in mesothelioma based on *TP53* genotype

**DOI:** 10.1371/journal.pone.0361362

**Published:** 2026-10-26

**Authors:** The PLOS One Editors

The *PLOS One* Editors issue this Correction to resolve the following issues with the article:

- The following blot panels appear similar, despite their respective uncropped blots in the Supporting information files in [1] appearing different:
  - Fig 2B NCI-H2452 P-MDM2 and Fig 2B EHMES-10 P-MDM2
  - Fig 3B NCI-H226 P-p53 and Fig 4B NCI-H226 P-p53
  - Fig 3B JMN-1B P-FAK and Fig 4B JMN-1B P-FAK
  - Fig 4B MSTO-211H Actin and Fig 4B EHMES-1 Actin
- The following blot panels appear different to their respective uncropped blots in the Supporting information files in [1]:
  - Fig 2B NCI-H2452 FAK
  - Fig 2B EHMES-10 FAK
  - Fig 2B EHMES-10 p53
  - Fig 2B NCI-H2052 P-AKT
  - Fig 2B NCI-H2452 P-AKT
  - Fig 3B NCI-H28 P-FAK
- In Fig 5A:
  - The concentration of Defactinib in the NCI-H2452 panel should read 0.5 µM instead of 1 µM
  - In the EHMES-1 panel, the x-axis should read 0, 20, 30, 40, 50, 60 µM
- In Fig 6A, the concentration of Defactinib should be as follows: MSTO-211H: 1.5 µM, NCI-H2052: 0.5 µM, NCI-H226: 4 µM, NCI-H2452: 3uM, EHMES-10: 1.5 µM, EHMES-1: 0.5 µM.
- Contrary to the article’s Data Availability statement, the individual-level data files supporting the article’s results were not provided with the article.

The corresponding author stated that Fig 2B EHMES-10 P-MDM2 is incorrect and Fig 2B NCI-H2452 P-MDM2 is correct. They further stated that Fig 4B NCI-H226 P-p53 is incorrect and Fig 3B NCI-H226 P-p53 is correct. They provided replacement Figs 2B and 4B and stated that the respective underlying blots for these panels in the Supporting information figures in [1] are correct.

The corresponding author provided the original blot images underlying Fig 3B JMN-1B P-FAK and Fig 4B JMN-1B P-FAK in S1 File and the original blot images underlying Fig 4B MSTO-211H Actin and Fig 4B EHMES-1 Actin in S2 File. They provided an updated version of Fig 3B with a replacement JMN-1B P-FAK panel, and an updated version of Fig 4B with replacement JMN-1B P-FAK, MSTO-211H Actin, and EHMES-1 Actin panels from the original blot images.

Regarding the differences between the published panels and their respective uncropped blots for Fig 2B NCI-H2452 FAK, Fig 2B EHMES-10 FAK, and Fig 2B NCI-H2452 P-AKT, the corresponding author stated the published panels are correct and the respective underlying blots in the Supporting information figures in [1] are incorrect, and that the wrong replicates for these panels were presented in Figs S3 and S8 in [1]. They provided the correct underlying blot for Fig 2B NCI-H2452 FAK and Fig 2B EHMES-10 FAK and a revised Fig S3 in S3 File. For Fig 2B NCI-H2452 P-AKT the correct underlying blot is presented in S4 File and the revised Fig S8 in S5 File.

Regarding the differences between the published panels and their respective uncropped blots for Fig 2B EHMES-10 p53 and Fig 2B NCI-H2052 P-AKT, the corresponding author stated that these published panels in [1] are correct and show a short exposure and the corresponding Supporting information figures in [1] show a long exposure of the same blot. They provided the short exposure versions for Fig 2B EHMES-10 p53 and a revised Fig S5 in S6 File, and the short exposure blot for Fig 2B NCI-H2052 P-AKT in S7 File and the revised Fig S8 in S5 File.

The corresponding author stated that a short exposure version of the blot was used in Fig 3B NCI-H28 P-FAK and a long exposure blot was presented in the Supporting information figures in [1]. They provided the short exposure uncropped blot in S8 File and an updated version of Fig S15 in S9 File**.** They provided an updated version of Fig 3 with a replacement Fig 3B NCI-H28 P-FAK panels from the original blot images.

A member of the *PLOS One* Editorial Board reviewed the updated concentration information in Fig 5A and 6A and stated that this does not affect the main results or conclusions of the article.

The corresponding author provided updated versions of Figs 2–6 and the individual-level quantitative data underlying the published results (S10 File). With this correction, all relevant data are now provided.

During post-publication discussions, the corresponding author stated that the blot panels presented within a given figure in [1] were not detected on a single, uncut blot simultaneously nor were they run on the same day, but that the proteins were derived from the same sample preparation and the aliquots of the samples were stored under −90 degrees to prevent degradation. They further stated the electrophoresis, transfer, and antibody incubation were identical between experimental runs, and the same amount of protein was loaded into each lane to ensure quantitative accuracy. The same blot was used for multiple proteins in some cases after confirmation that the blot had low background and the molecular size of the target protein was quite different.

**Fig 2:**
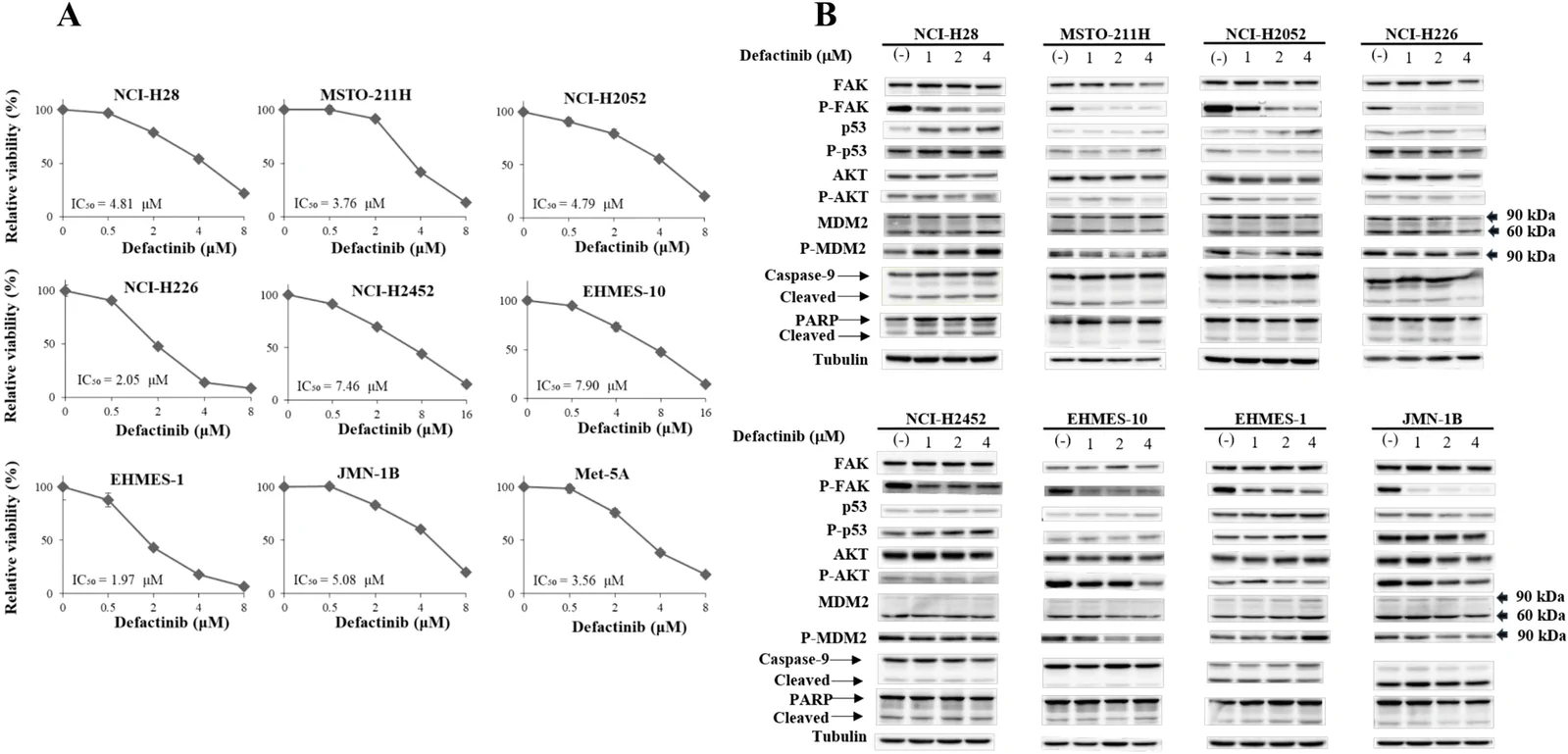
Growth inhibition and molecular changes induced by defactinib. (A) Growth inhibitory effects of defactinib on mesothelioma and Met-5A cells. Cell cells were treated with defactnib as indicated for 72 hrs and the viability was assayed with the WST assay. IC_50_ values and SE bars (n  =  3) are also included. (B) Expression level of the molecules associated with FAK and p53 in mesothelioma cells treated with defactinib as indicated for 24 hrs. The western blot analysis included expression levels of a phosphorylated form of FAK, p53 and AKT, and a cleaved form of caspase-9 and PARP. Tubulin was used as a loading control. The expression of each molecule was quantified with ImageJ software (NIH, Bethesda, MD, USA) with tubulin intensity as a normalized control (see S4 Table).

**Fig 3:**
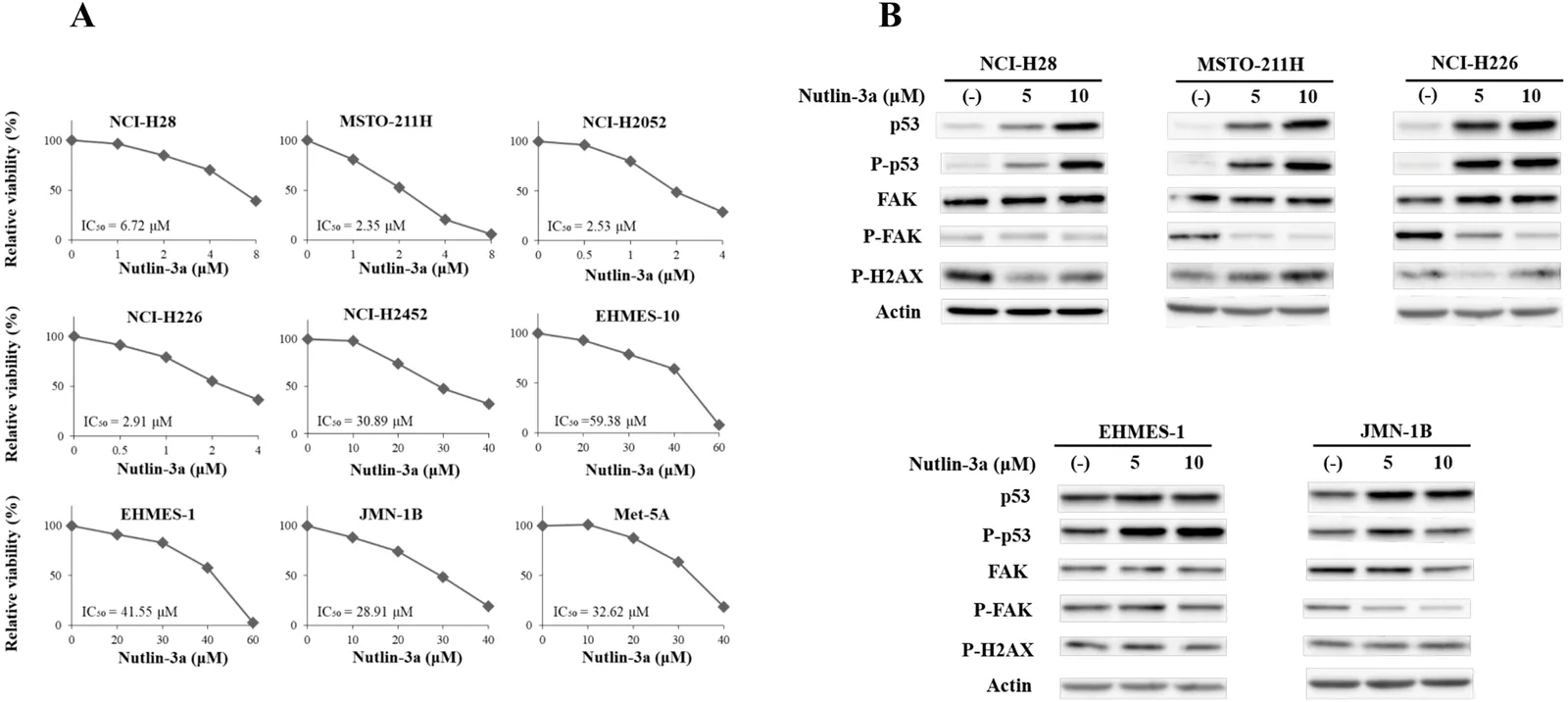
Growth inhibition and molecular changes induced by nutlin-3a. (A) Growth inhibitory effects of nutlin-3a on mesothelioma and Met-5A cells. Cell cells were treated with nutlin-3a as indicated for 72 hrs and the viability was assayed with the WST assay. IC_50_ values and SE bars (n  =  3) are also included. (B) Expression level of the molecules associated with FAK and p53 in mesothelioma cells treated with nultin-3a as indicated for 24 hrs. The western blot analysis also included expression levels of a phosphorylated form of FAK, p53 and H2AX. Actin was used as a loading control. For phosphorylated H2AX, the blots were exposed for a longer period to ensure the detection of basal signals in untreated cells. The basal intensity therefore differed from that of the RITA experiments in Fig 4B where shorter exposures were used. The expression of each molecule was quantified with ImageJ software (NIH, Bethesda, MD, USA) with actin intensity as a normalized control (see S5 Table).

**Fig 4:**
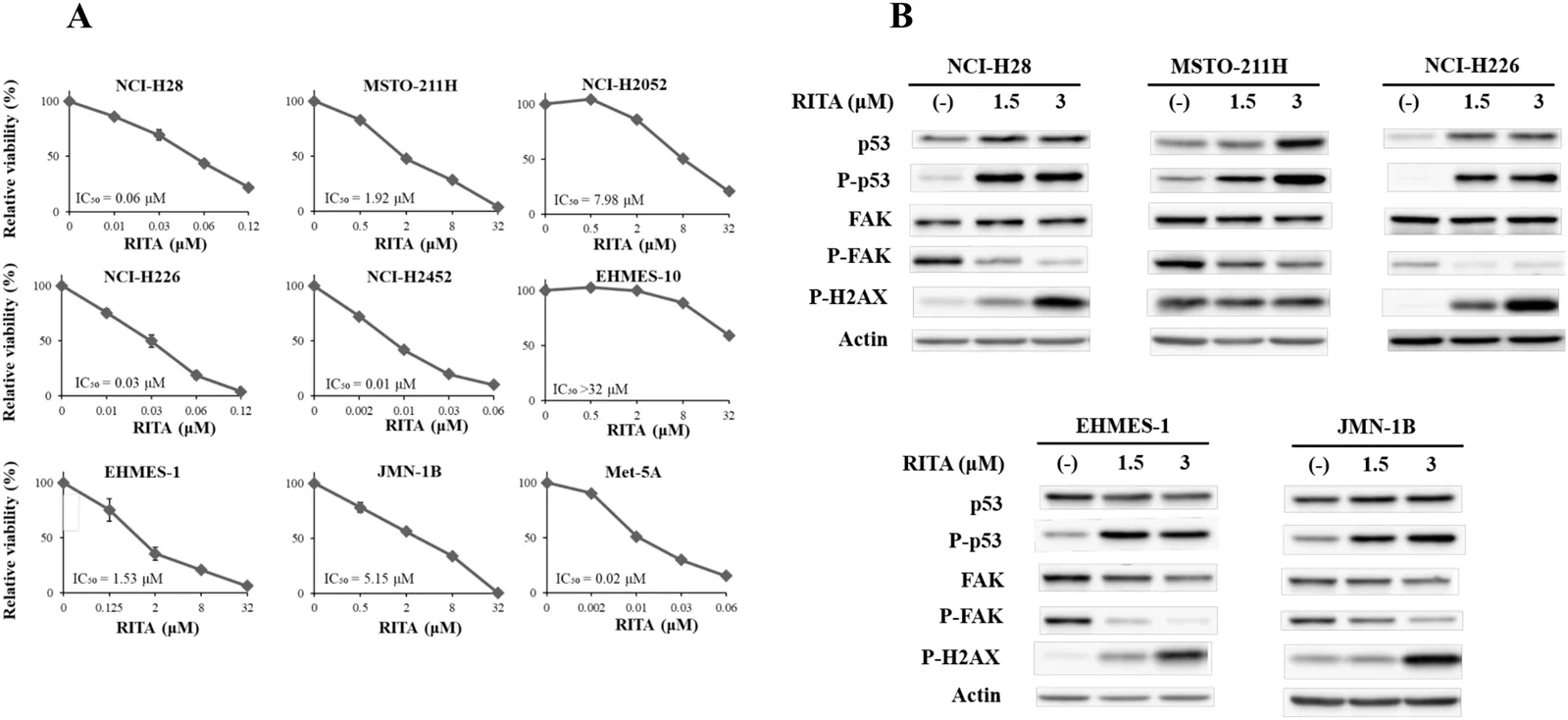
Growth inhibition and molecular changes induced by RITA. (A) Growth inhibitory effects of RITA on mesothelioma and Met-5A cells. Cell cells were treated with RITA as indicated for 72 hrs and the viability was assayed with the WST assay. IC_50_ values and SE bars (n  =  3) are also included. (B) Expression level of the molecules associated with FAK and p53 in mesothelioma cells treated with RITA as indicated for 24 hrs. The western blot analysis included expression levels of a phosphorylated form of FAK, p53 and H2AX. Actin was used as a loading control. For phosphorylated H2AX of MSTO-211H cells, the blot was exposed for a longer period to ensure the detection of basal signals in untreated cells. The expression of each molecule was quantified with ImageJ software (NIH, Bethesda, MD, USA) with actin intensity as a normalized control (see S6 Table).

**Fig 5:**
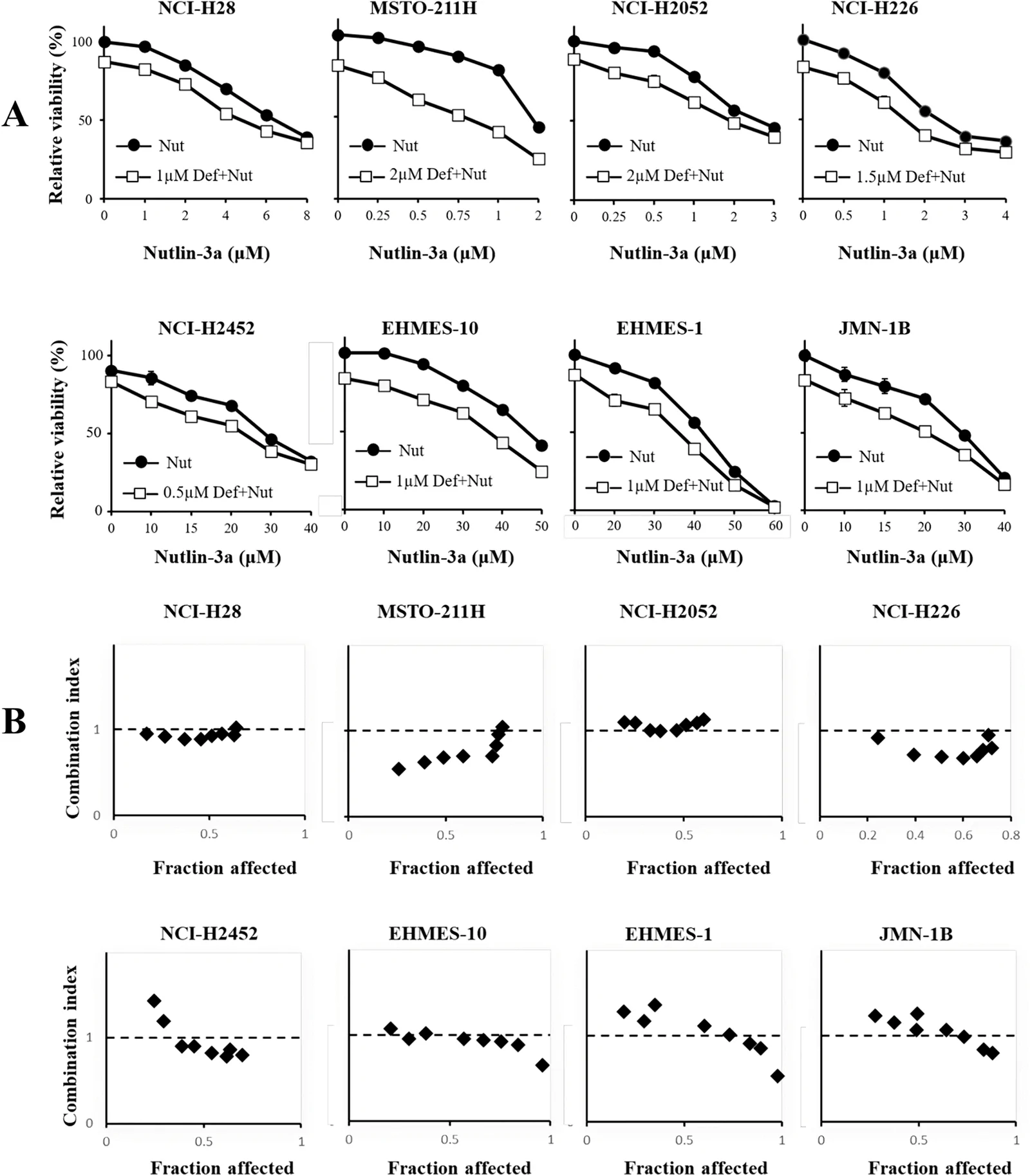
Combination of defactinib and nutlin-3a. (A) Combinatory effects of defactinib and nutlin-3a on mesothelioma. Cells were treated with defactinib and nutlin-3a as indicated for 72 hrs and the viability was assayed with the WST assay. SE bars (n  =  3) are shown. (B) The combinatory effects were examined with the CalcuSyn software and the CI values at respective Fa points are shown.

**Fig 6:**
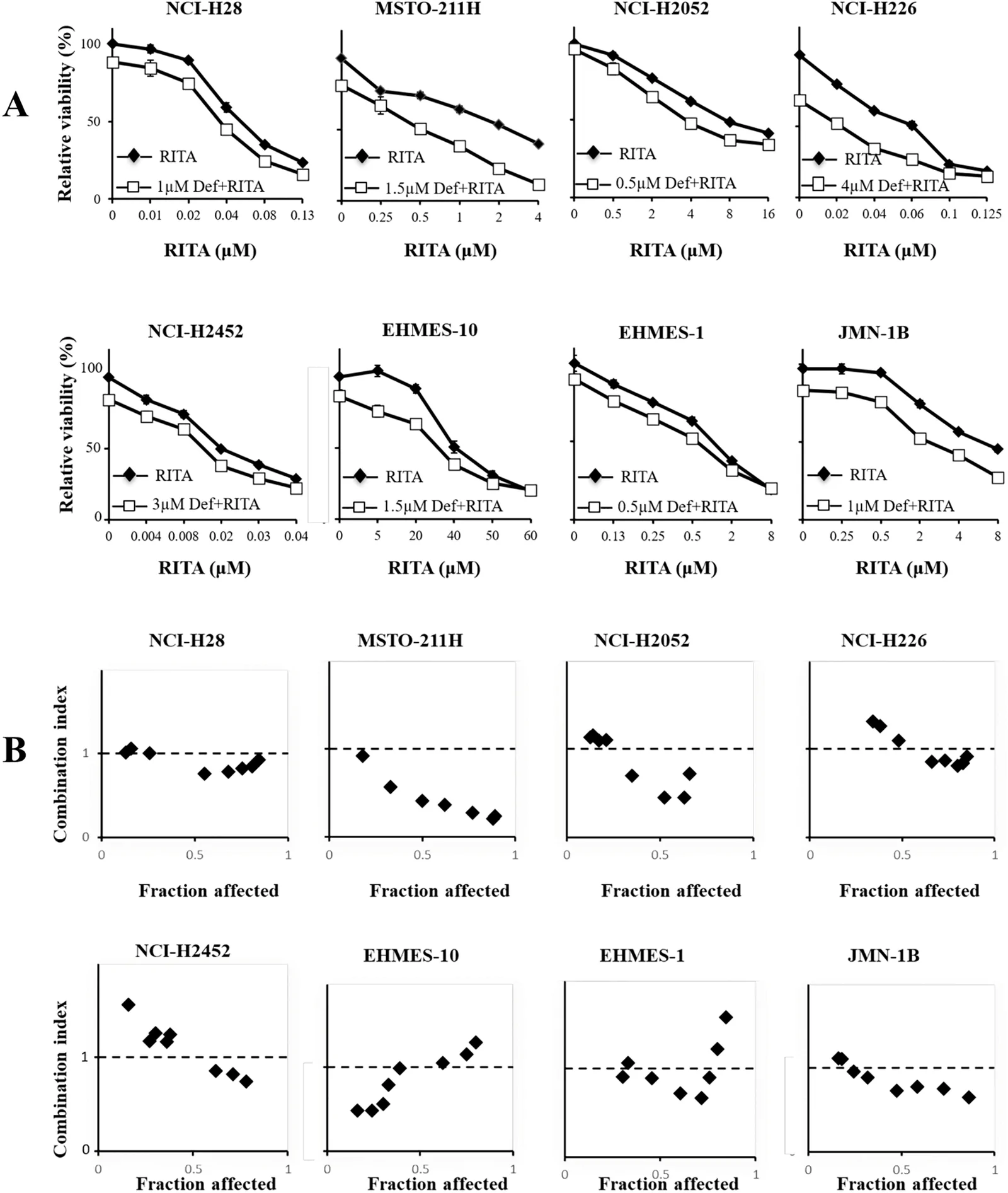
Combination of defactinib and RITA. (A) Combinatory effects of defactinib and RITA on mesothelioma. Cells were treated with defactinib and RITA as indicated for 72 hrs and the viability was assayed with the WST assay. SE bars (n  =  3) are shown. (B) The combinatory effects were examined with the CalcuSyn software and the CI values at respective Fa points are shown.

## Supporting information

S1 File Original blot images underlying Fig 3B JMN-1B P-FAK and Fig 4B JMN-1B P-FAK. (ZIP)

S2 File Original blot image underlying Fig 4B MSTO-211H and Fig 4B EHMES-1 Actin panels. (ZIP)

S3 File Underlying blot data for Fig 2B FAK. Including: original uncropped blot image, revised Fig S3.
(ZIP)

S4 File Updated uncropped blot Fig 2B NCI-H2452 P-AKT. (PDF)

S5 File Revised Fig S8. Showing short exposure Figure 2B p-AKT used in [1].
(PDF)

S6 File Underlying blot data Fig 2B EHMES-10 p53. Including: short exposure and long exposure uncropped blot, revised Fig S5.
(ZIP)

S7 File Short exposure uncropped blot Fig 2B NCI-H2052 P-AKT. (PNG)

S8 File Short exposure uncropped blot Fig 3B NCI-H28 P-FAK. The right hand lanes correspond to Fig 3B NCI-H28 P-FAK.
(ZIP)

S9 File Revised Fig S15. (PDF)

S10 File Individual-level quantitative data underlying the published results. (ZIP)
