# Supplementary figures and images for: Strategic selection of MDM2 inhibitors enhances the efficacy of FAK inhibition in mesothelioma based on TP53 genotype
Source: PLoS One. 2026 Feb 23;21(2):e0343551. doi: 10.1371/journal.pone.0343551 (PMC12928570; doi:10.1371/journal.pone.0343551)

Supplementary Figure 1

(A)

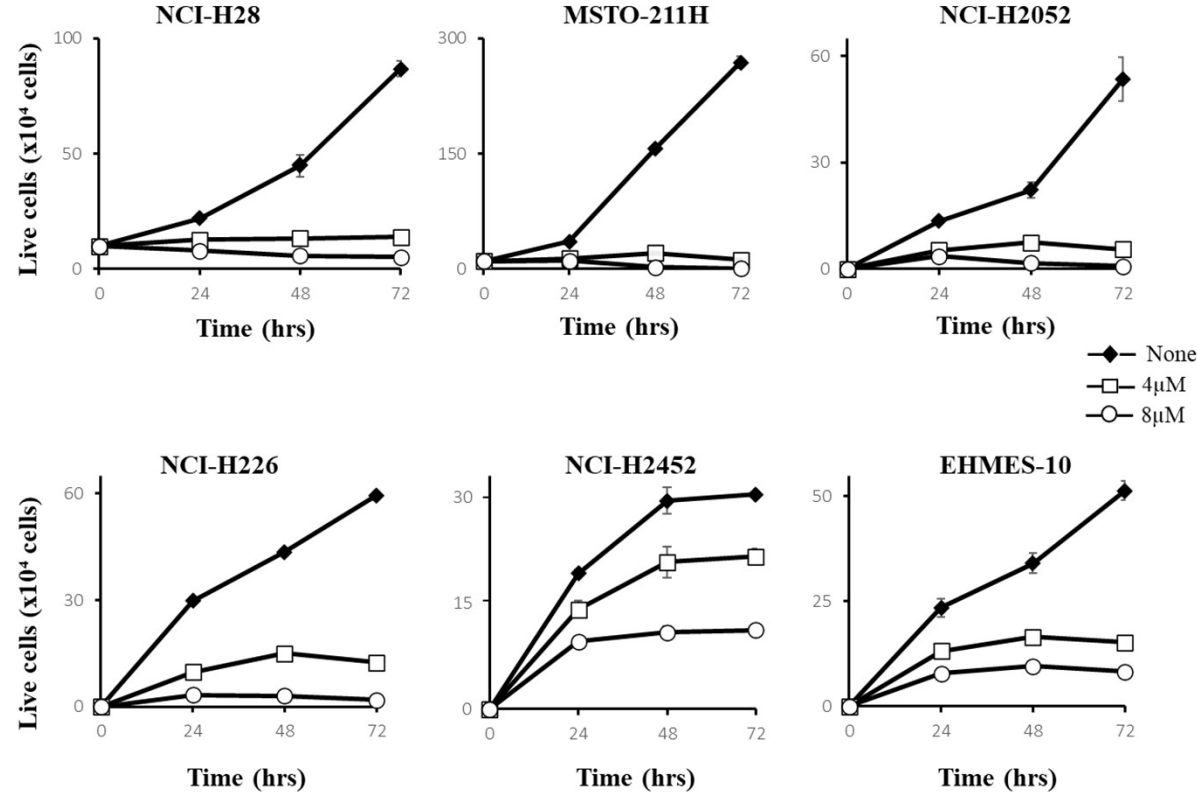

(B)

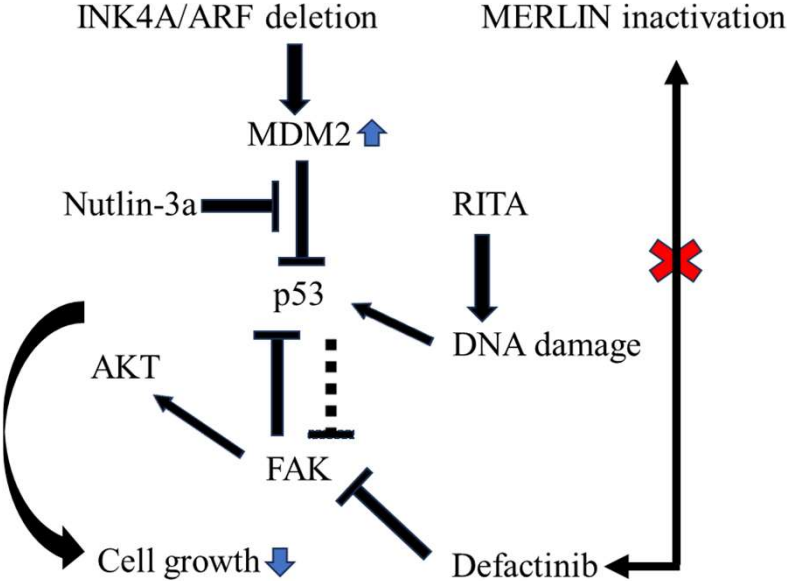

Supplement: S1 Fig — (A) Defactinib-mediated inhibition of mesothelioma cell proliferation with a dye exclusion assay. Cells were seeded in 6 well-plates and treated with defactinib for 24–72 hrs. They were then stained with 0.4% trypan blue solution (Sigma-Aldrich, St. Louis, MO) for 3 minutes at room temperature. The number of stained and unstained cells was counted and the assay were tested in triplicate. (B) A schema of the present study. Mesothelioma cells often have deletion of INK4A/ARF region and MERLIN inactivation. These characteristics induce up-regulated MDM2 expression with subsequent p53 down-regulation. An MDM2 and a FAK inhibitor can augment p53 expression and induce growth inhibition. The current study indicated that nutlin-3a and RITA, a representative MDM2 inhibitor, up-regulated p53 expression via a different mechanism and that the growth inhibition by defactinib, a FAK inhibitor, was unrelated MERLIN expression. A combination of the MDM2 inhibitor and the FAK inhibitor achieved synergistic or additive inhibitory effects, and the effects were linked with the AKT signaling. Nevertheless, the growth inhibitory activity was also subjected to the kind of the MDM2 inhibitor and cells used. (PDF) [file pone.0343551.s001.pdf]

## Slide 1
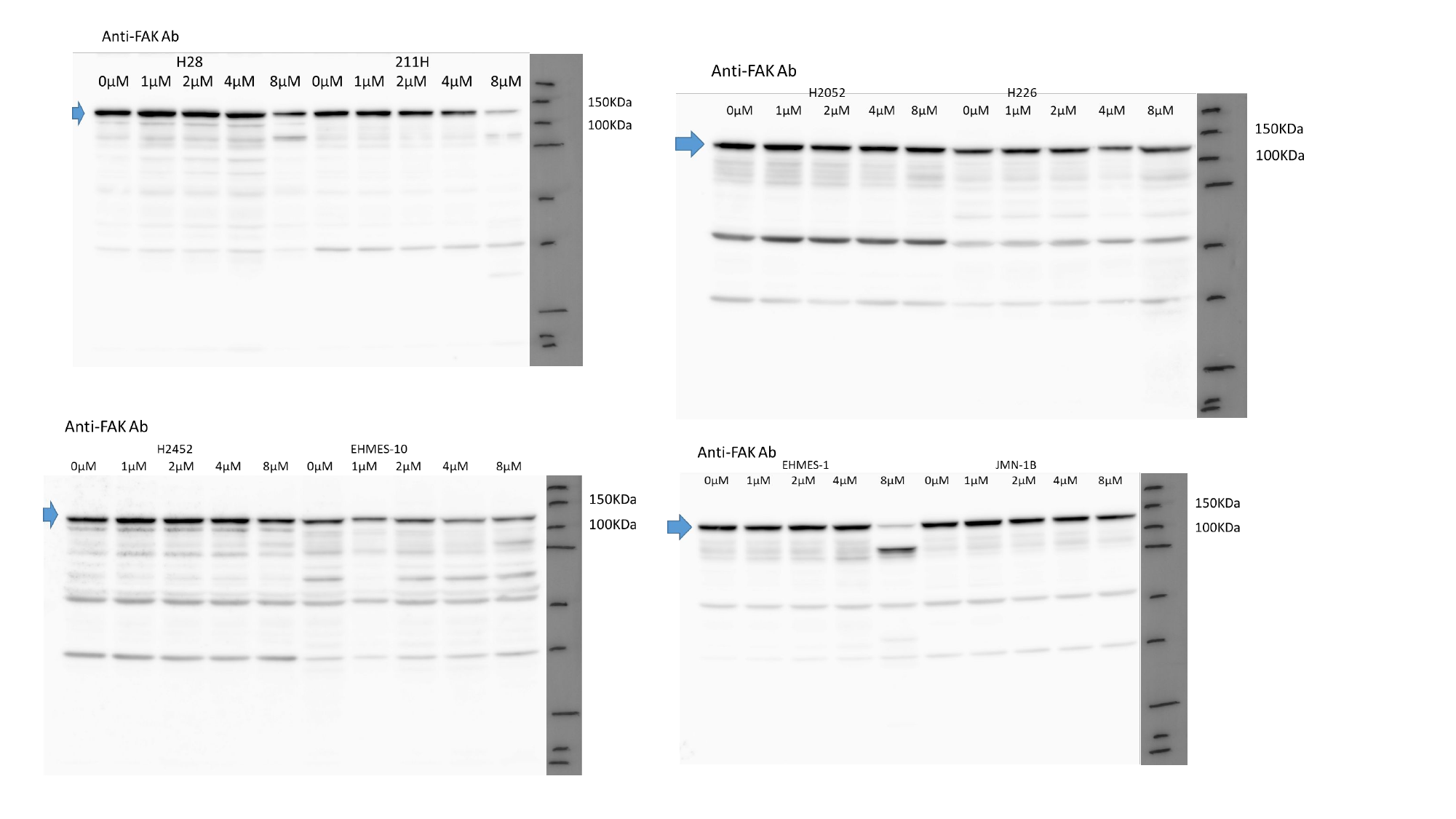

Supplement: S3 Fig — Original blots which were used for Fig 2B FAK expression. Arrows indicate the target molecules. The name of cells was shown in the abbreviations. We did not use a photo of 8 μM defactinib treatments. In some of the blots, we used the same blot to detect others molecules without stripping the blot and consequently showed the target molecules by the arrows. (PPTX) [file pone.0343551.s003.pptx]

## Slide 1
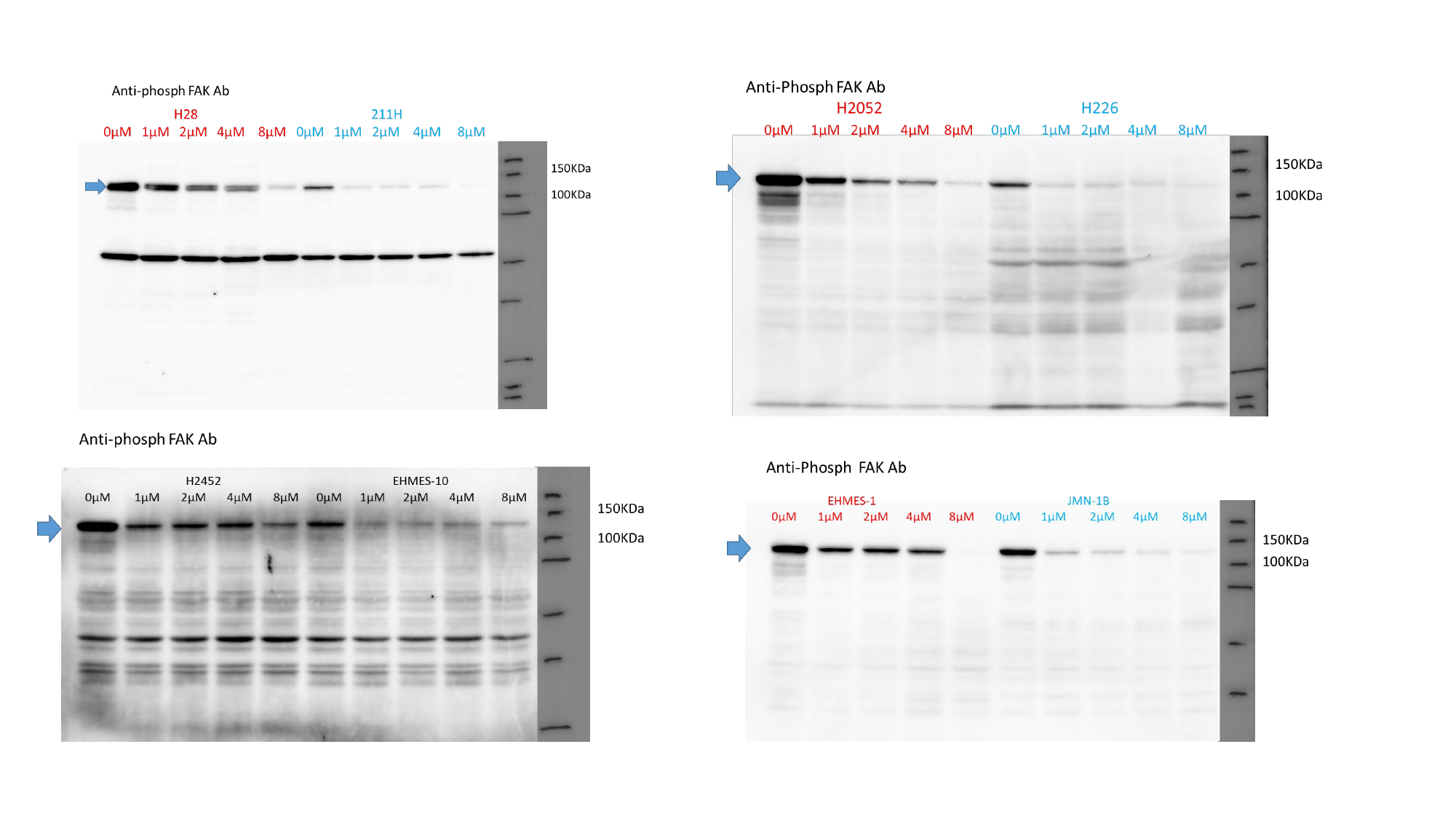

Supplement: S4 Fig — Original blots which were used for Fig 2B phosphorylated FAK expression. Arrows indicate the target molecules. The name of cells was shown in the abbreviations. We did not use a photo of 8 μM defactinib treatments. In some of the blots, we used the same blot to detect others molecules without stripping the blot and consequently showed the target molecules by the arrows. (PPTX) [file pone.0343551.s004.pptx]

## Slide 1
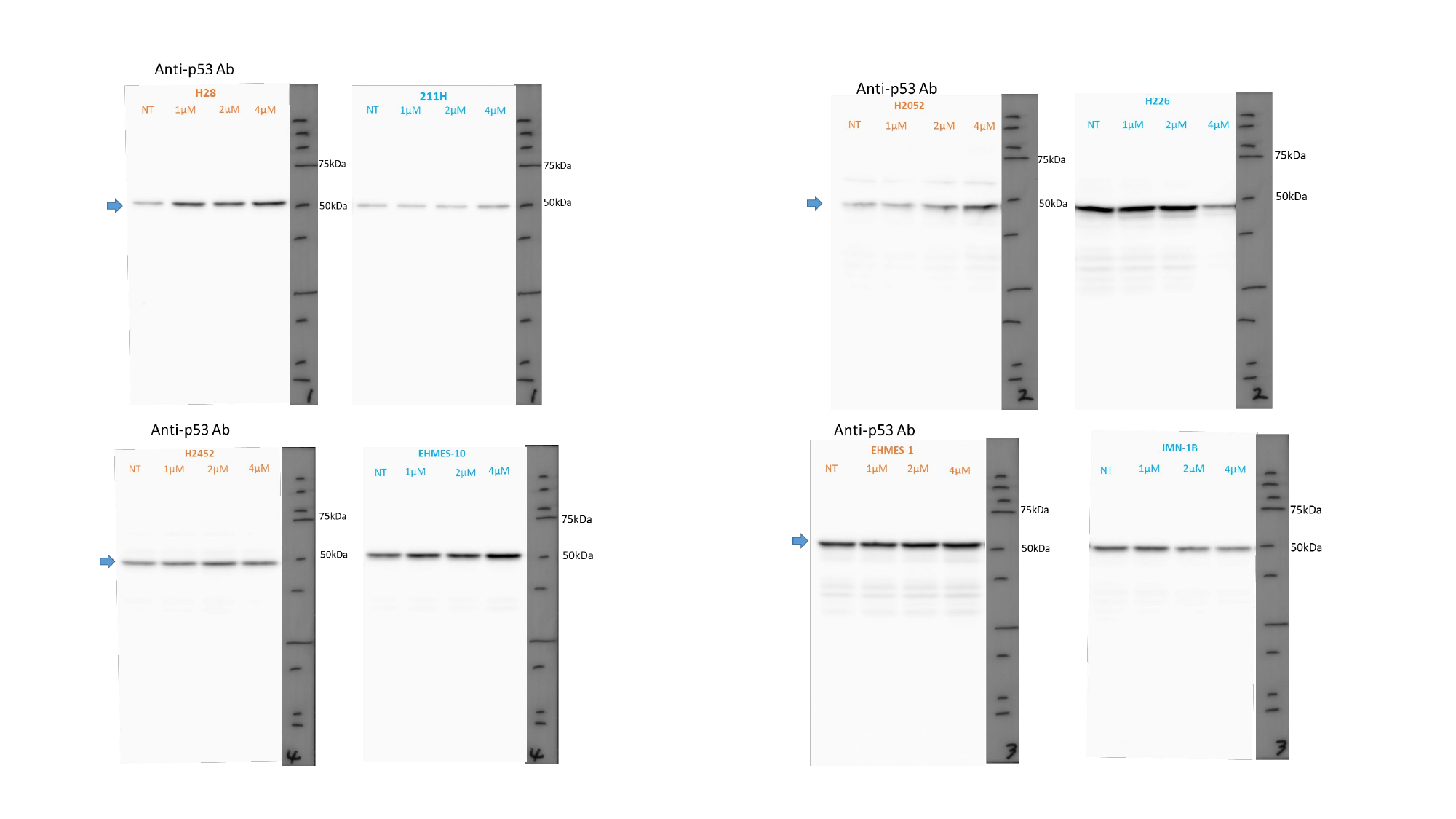

Supplement: S5 Fig — Original blots which were used for Fig 2B p53 expression. Arrows indicate the target molecules (NCI-H2452 had a truncated p53). The name of cells was shown in the abbreviations. (PPTX) [file pone.0343551.s005.pptx]

## Slide 1
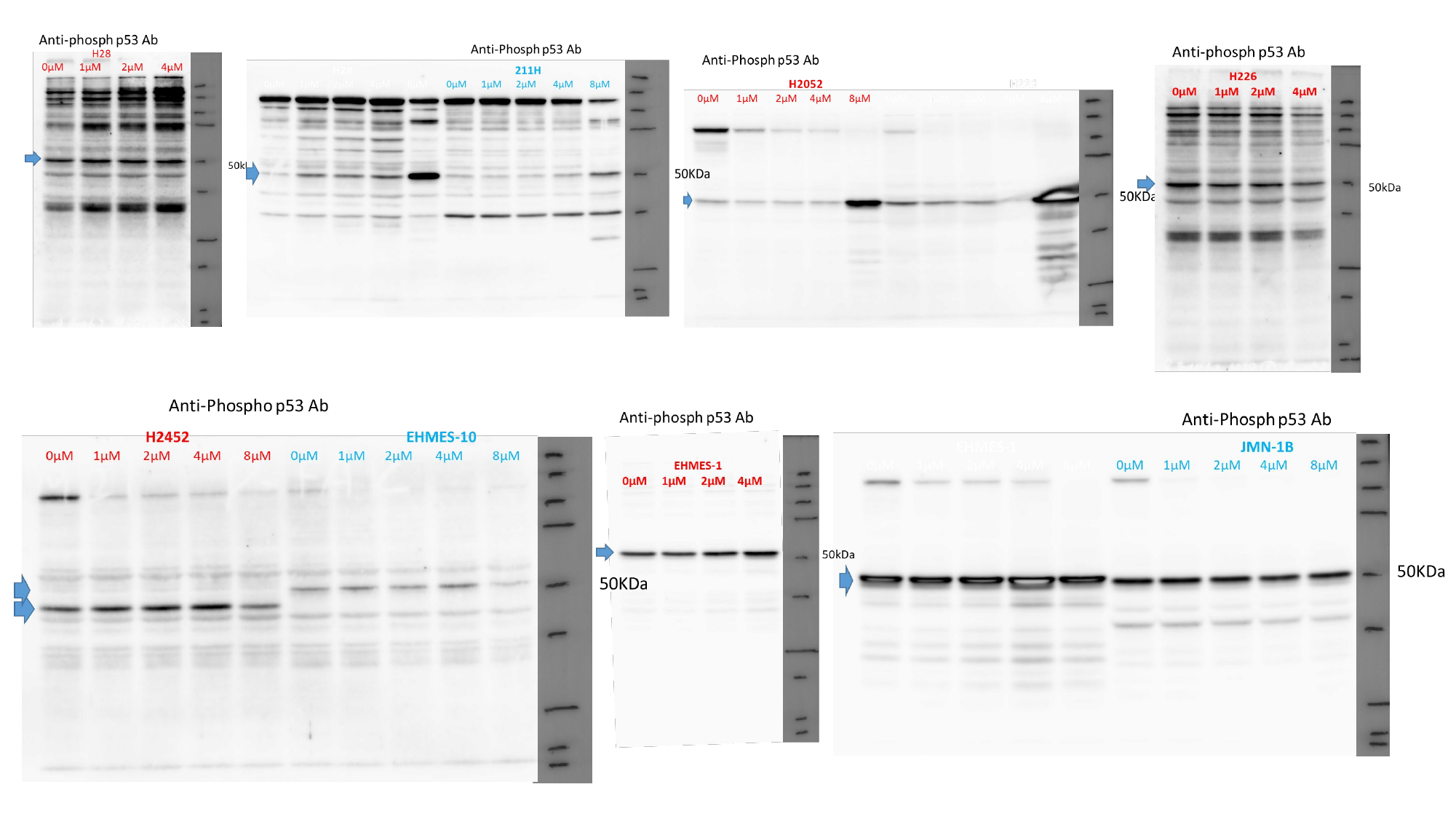

Supplement: S6 Fig — Original blots which were used for Fig 2B phosphorylated p53 expression. Arrows indicate the target molecules (NCI-H2452 had a truncated p53). The name of cells was shown in the abbreviations. We did not use a photo of 8 μM defactinib treatments. In some of the blots, we used the same blot to detect others molecules without stripping the blot and consequently showed the target molecules by the arrows. (PPTX) [file pone.0343551.s006.pptx]

## Slide 1
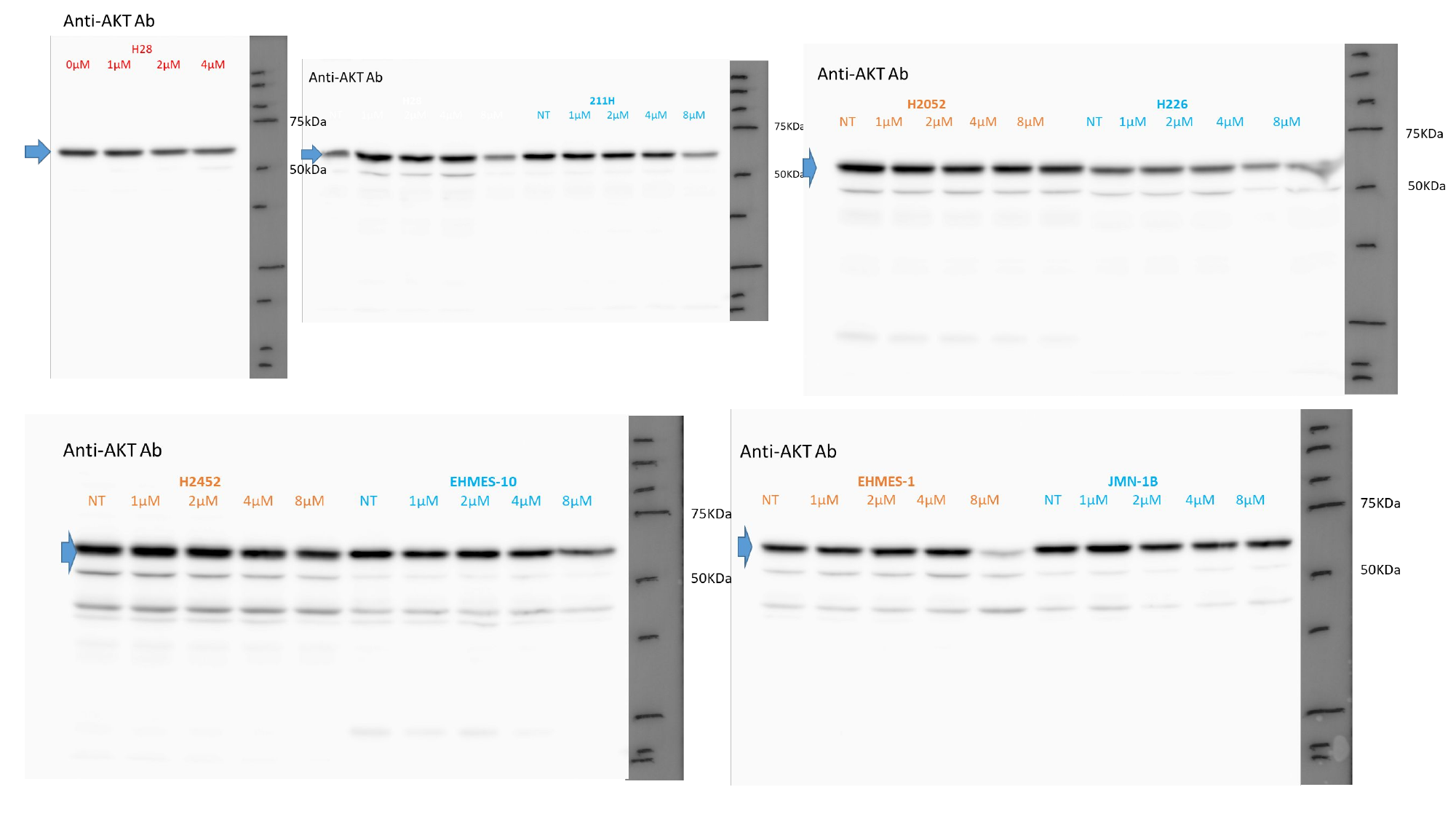

Supplement: S7 Fig — Original blots which were used for Fig 2B AKT expression. Arrows indicate the target molecules. The name of cells was shown in the abbreviations. We did not use a photo of 8 μM defactinib treatments. (PPTX) [file pone.0343551.s007.pptx]

## Slide 1
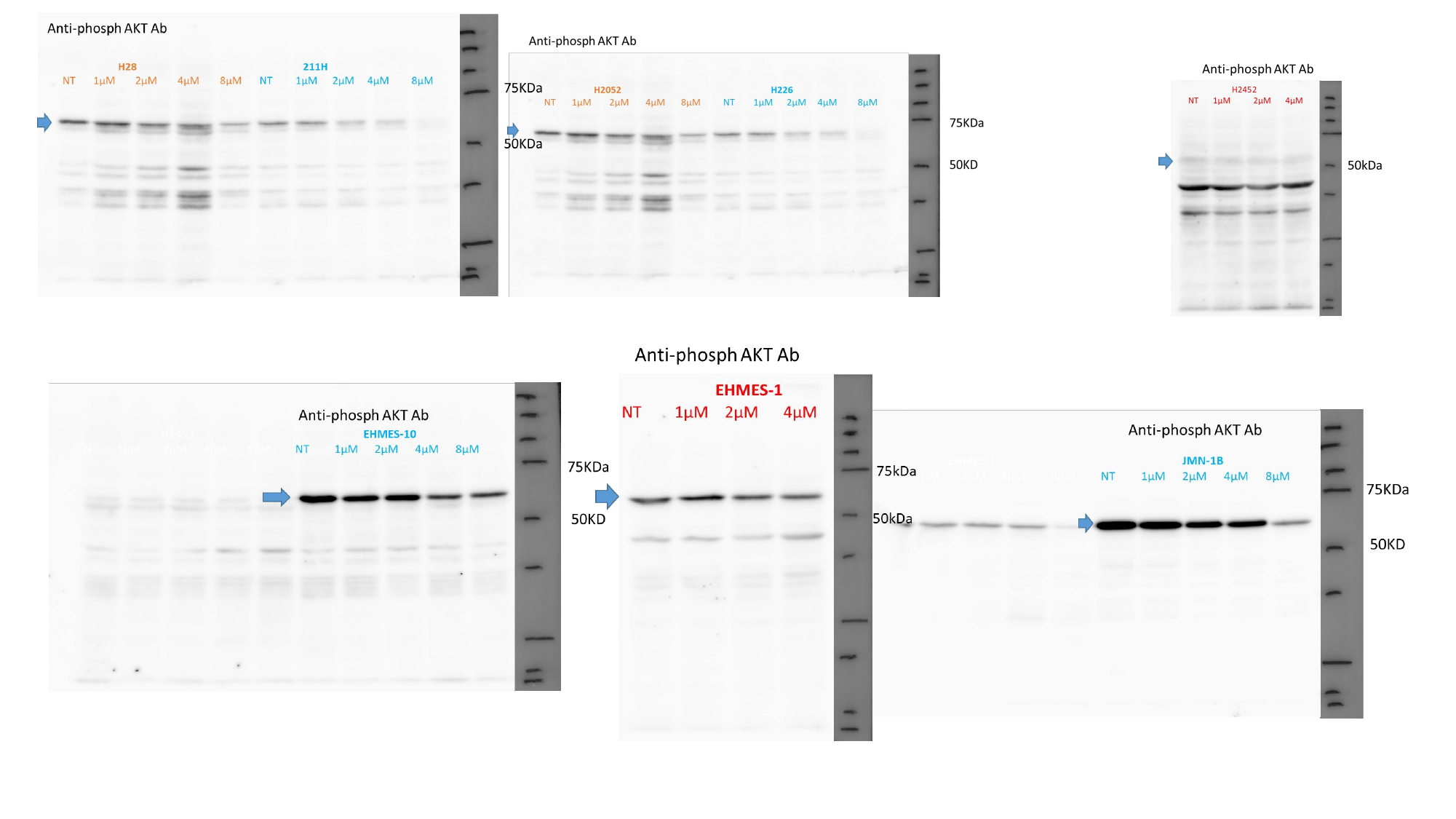

Supplement: S8 Fig — Original blots which were used for Fig 2B phosphorylated AKT expression. Arrows indicate the target molecules. The name of cells was shown in the abbreviations. We did not use a photo of 8 μM defactinib treatments. In some of the blots, we used the same blot to detect others molecules without stripping the blot and consequently showed the target molecules by the arrows. (PPTX) [file pone.0343551.s008.pptx]

## Slide 1
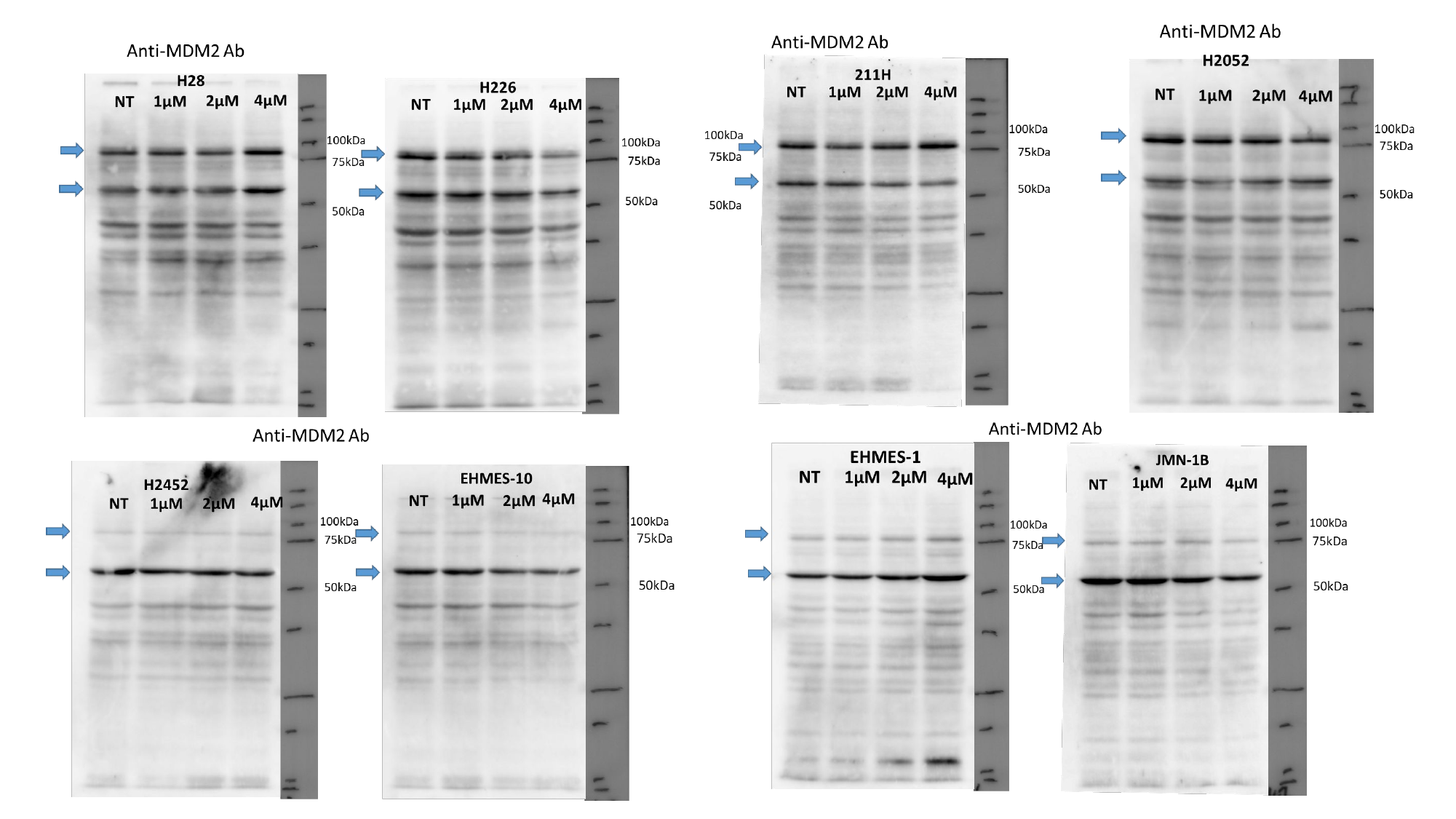

Supplement: S9 Fig — Original blots which were used for Fig 2B MDM2 expression. Arrows indicate the target molecules (both 90 kDa and 60 kDa molecules). The name of cells was shown in the abbreviations. (PPTX) [file pone.0343551.s009.pptx]

## Slide 1
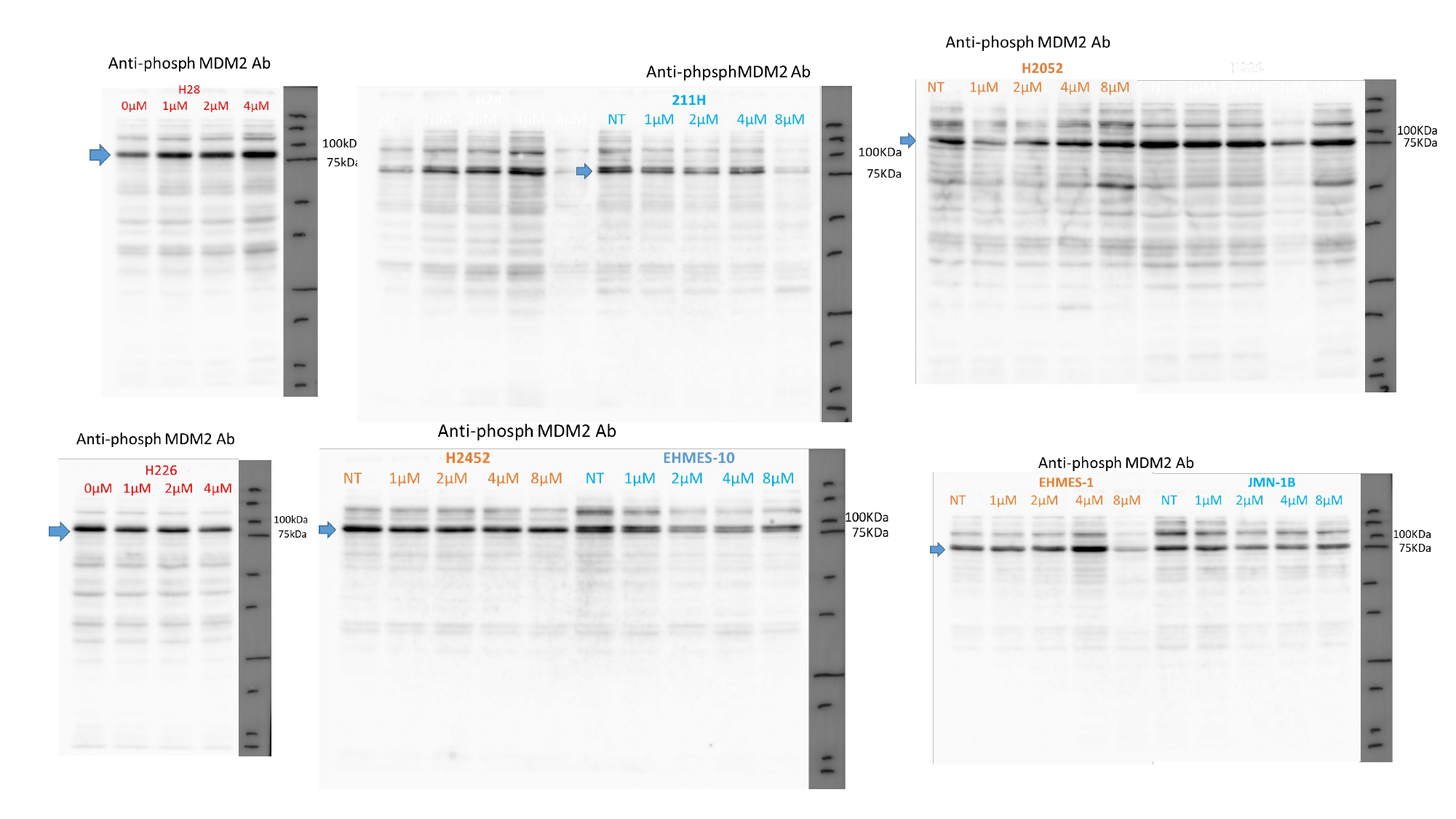

Supplement: S10 Fig — Original blots which were used for Fig 2B phosphorylated MDM2 expression. Arrows indicate the target molecules (90 kDa). The name of cells was shown in the abbreviations. We did not use a photo of 8 μM defactinib treatments. (PPTX) [file pone.0343551.s010.pptx]

## Slide 1
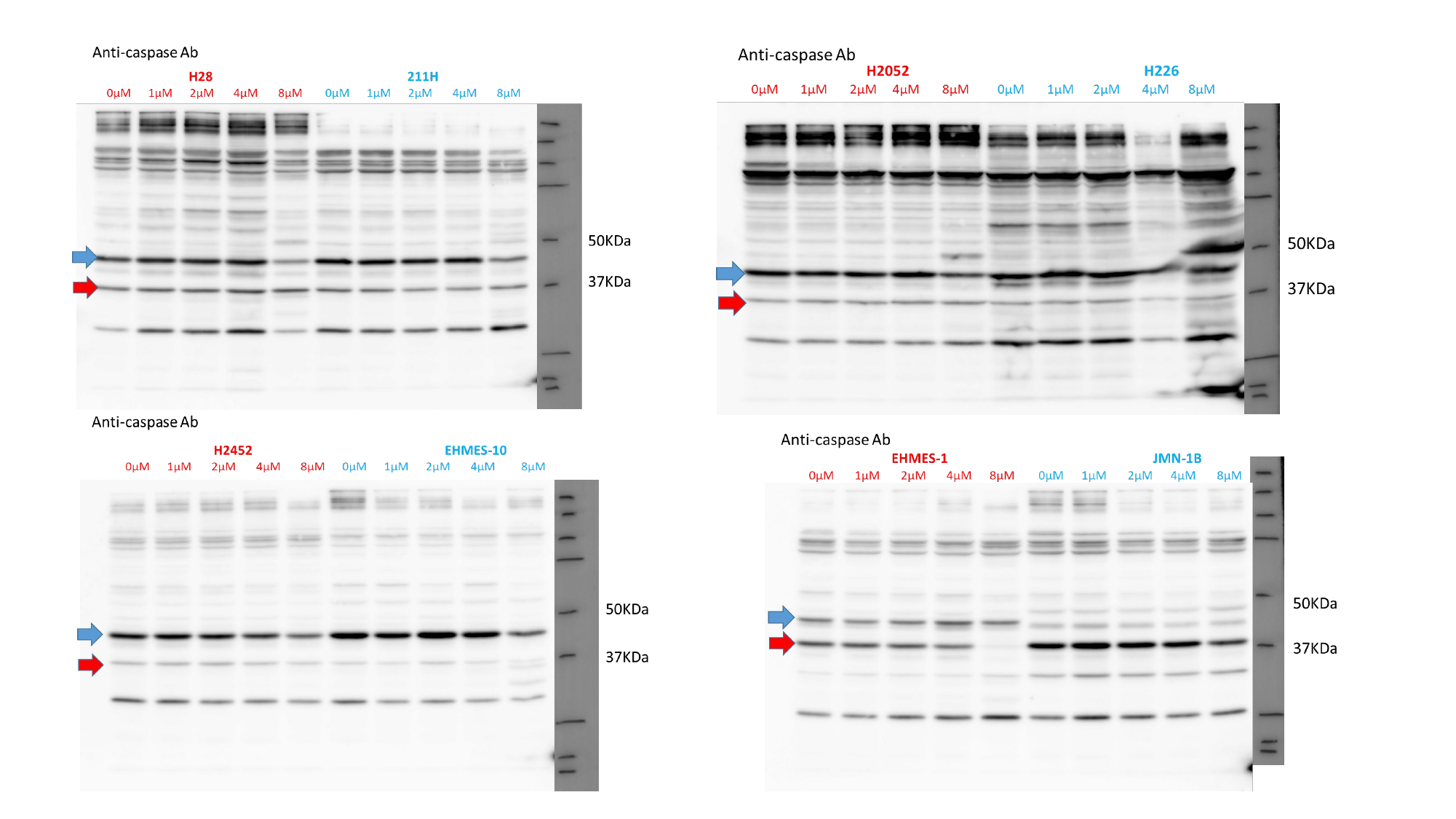

Supplement: S11 Fig — Original blots which were used for Fig 2B caspase-9 and the cleaved caspase-9 expressions. The antibody detected the cleaved form. Arrows indicate the target molecules (both original and cleaved molecules). The name of cells was shown in the abbreviations. We did not use a photo of 8 μM defactinib treatments. In some of the blots, we used the same blot to detect others molecules without stripping the blot and consequently showed the target molecules by the arrows. (PPTX) [file pone.0343551.s011.pptx]

## Slide 1
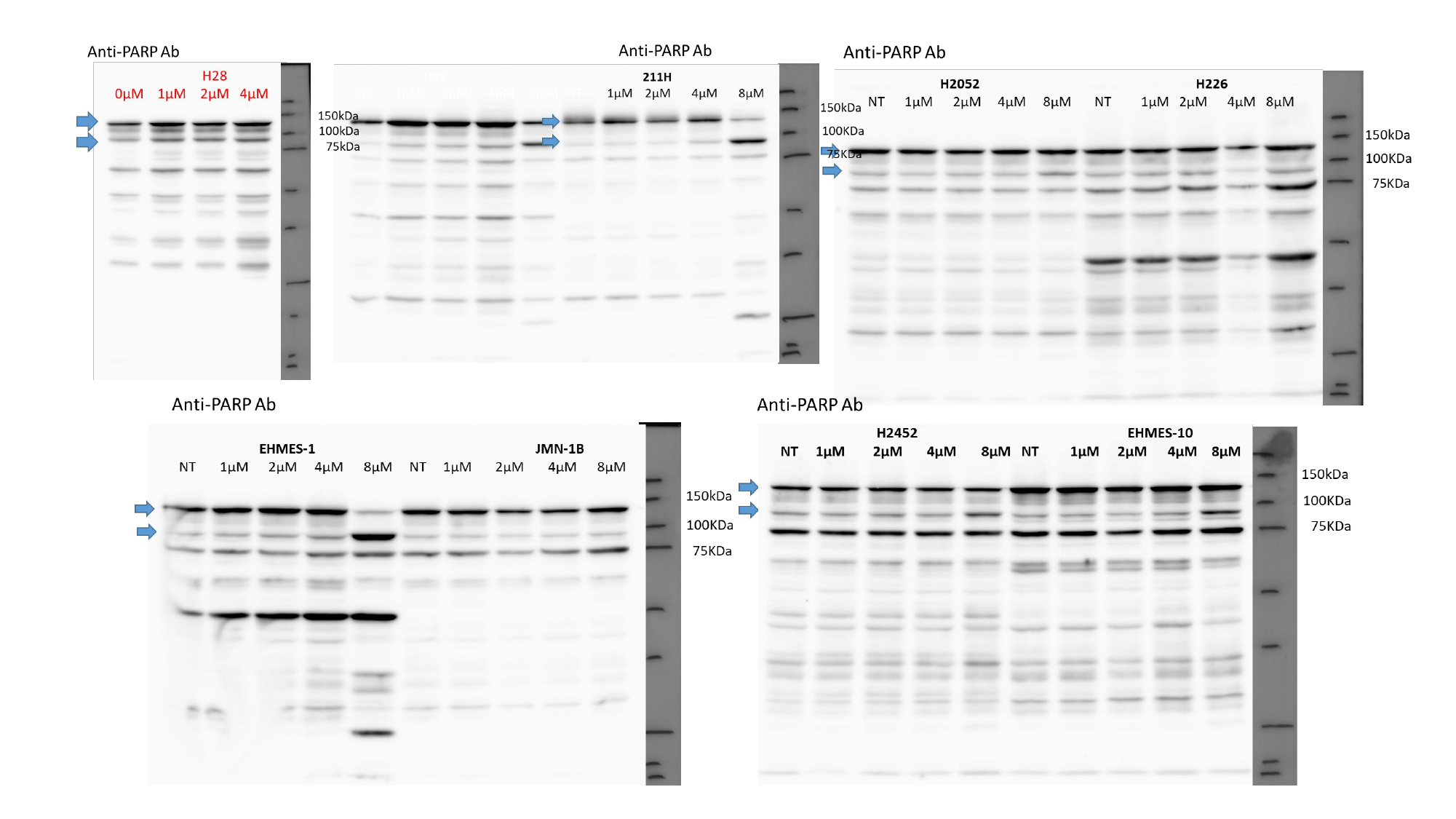

Supplement: S12 Fig — Original blots which were used for Fig 2B PARP and the cleaved PARP expressions. The antibody detected the cleaved form. Arrows indicate the target molecules (both original and cleaved molecules). The name of cells was shown in the abbreviations. We did not use a photo of 8 μM defactinib treatments. In some of the blots, we used the same blot to detect others molecules without stripping the blot and consequently showed the target molecules by the arrows. (PPTX) [file pone.0343551.s012.pptx]

## Slide 1
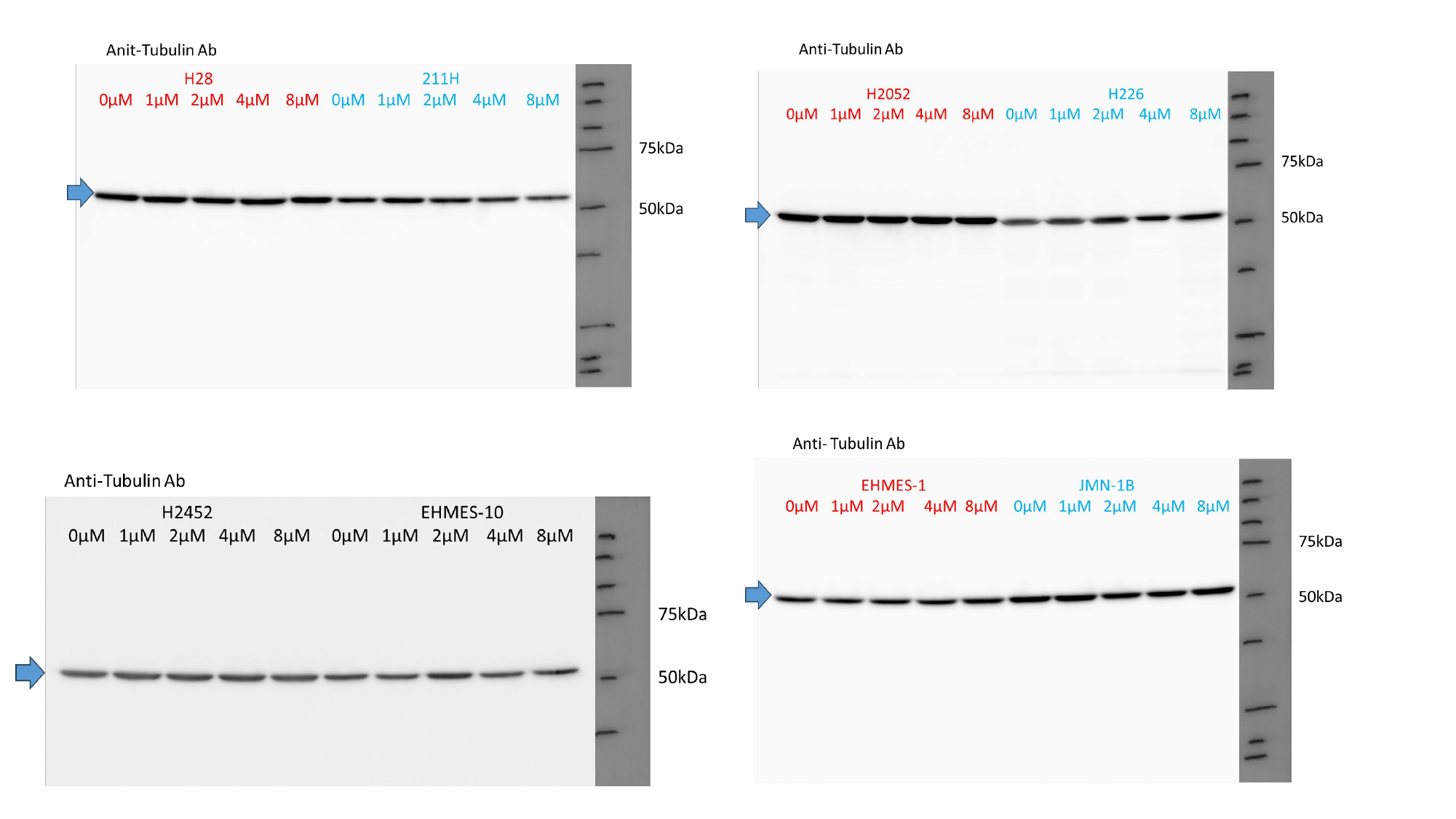

Supplement: S13 Fig — Original blots which were used for Fig 2B tubulin expression. Arrows indicate the target molecules. The name of cells was shown in the abbreviations. We did not use a photo of 8 μM defactinib treatments. (PPTX) [file pone.0343551.s013.pptx]

## Slide 1
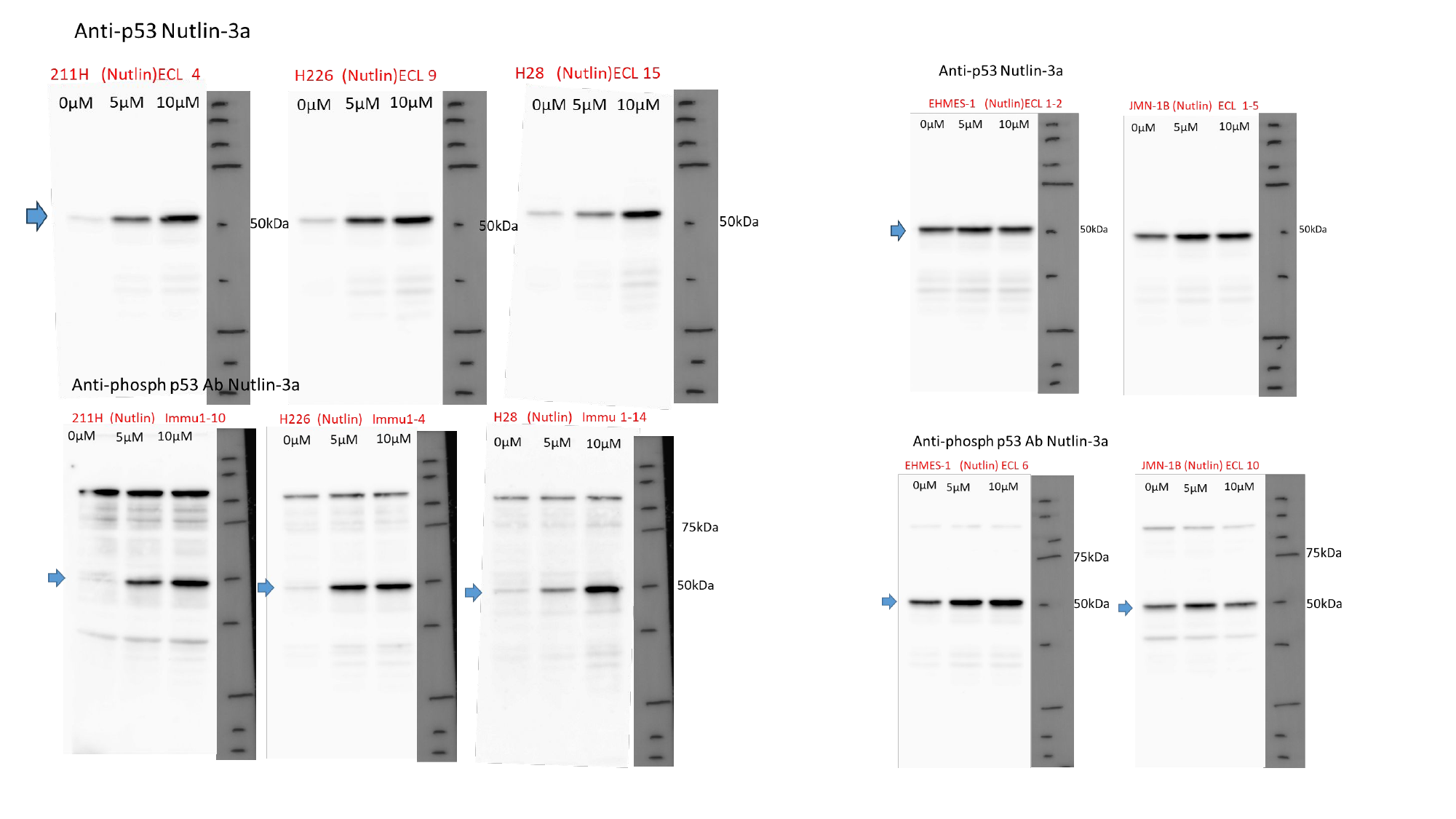

Supplement: S14 Fig — Original blots which were used for Fig 3B p53 and phosphorylated p53 expressions. Arrows indicate the target molecules. The name of cells was shown in the abbreviations. In some of the blots, we used the same blot to detect others molecules without stripping the blot and consequently showed the target molecules by the arrows. (PPTX) [file pone.0343551.s014.pptx]

## Slide 1
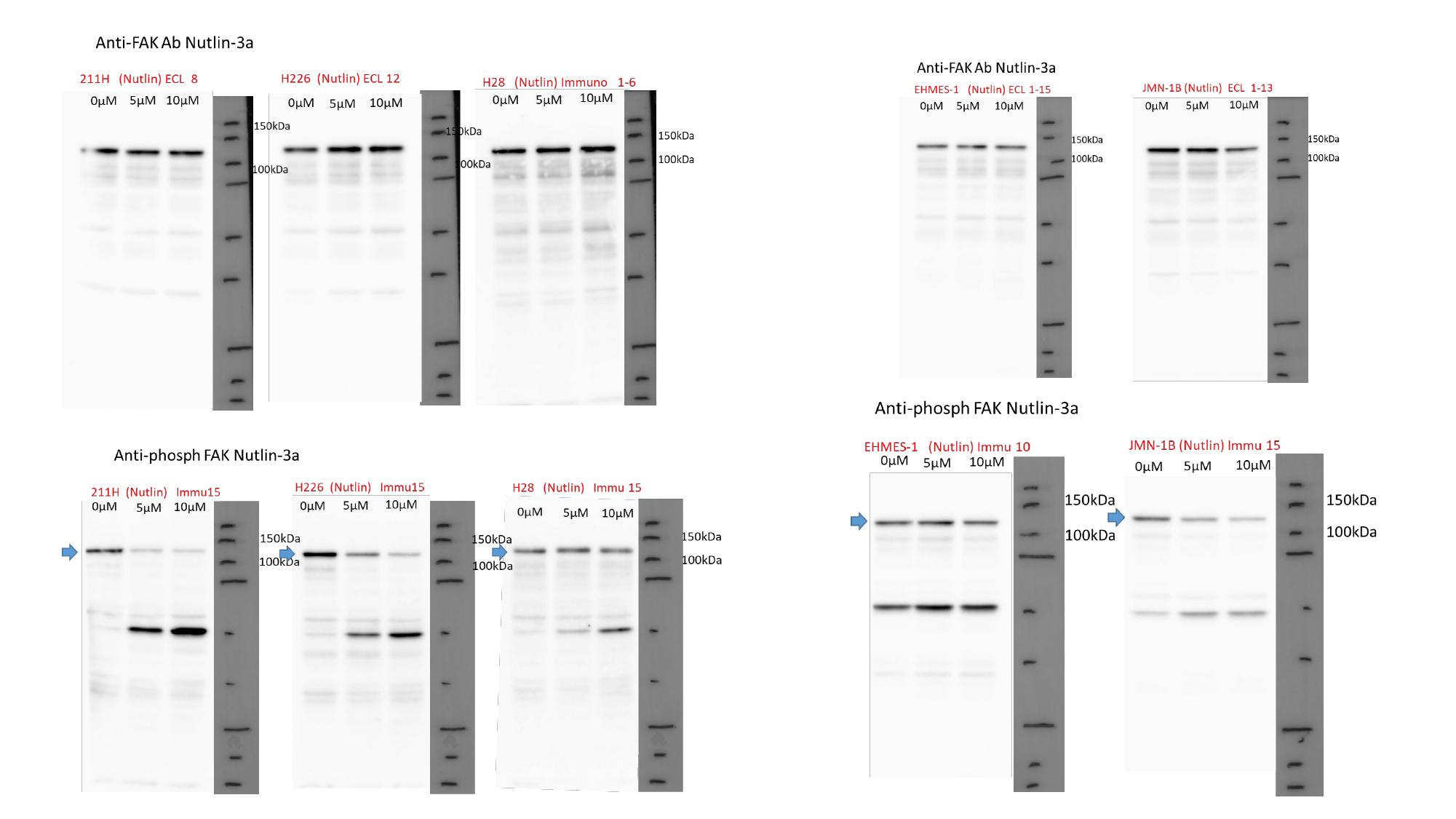

Supplement: S15 Fig — Original blots which were used for Fig 3B FAK and phosphorylated FAK expressions. Arrows indicate the target molecules. The name of cells was shown in the abbreviations. In some of the blots, we used the same blot to detect others molecules without stripping the blot and consequently showed the target molecules by the arrows. (PPTX) [file pone.0343551.s015.pptx]

## Slide 1
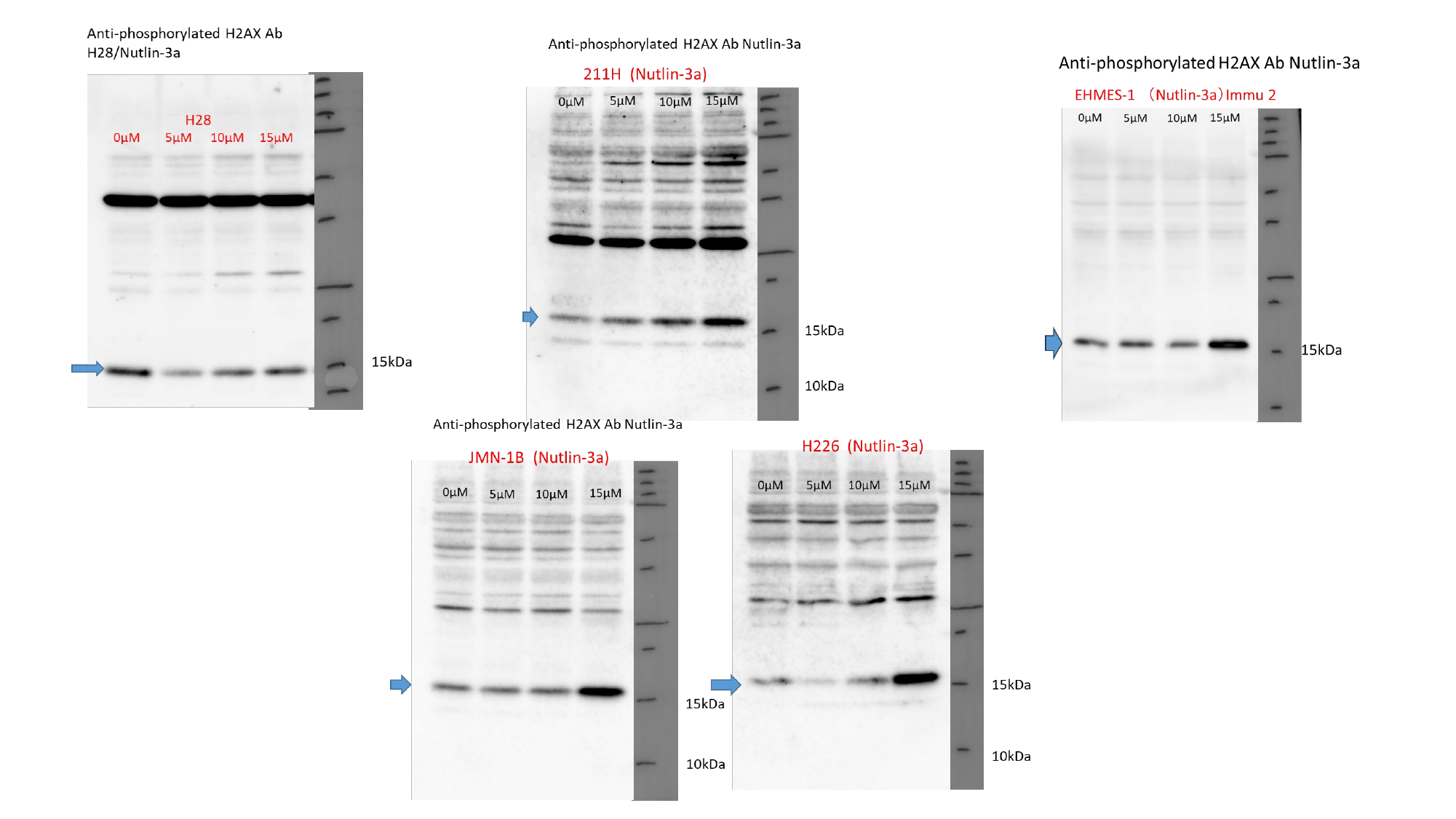

Supplement: S16 Fig — Original blots which were used for Fig 3B p-H2AX expression. Arrows indicate the target molecules. The name of cells was shown in the abbreviations. We did not use a photo of 15 μM nutlin-3a treatments. In some of the blots, we used the same blot to detect others molecules without stripping the blot and consequently showed the target molecules by the arrows. (PPTX) [file pone.0343551.s016.pptx]

## Slide 1
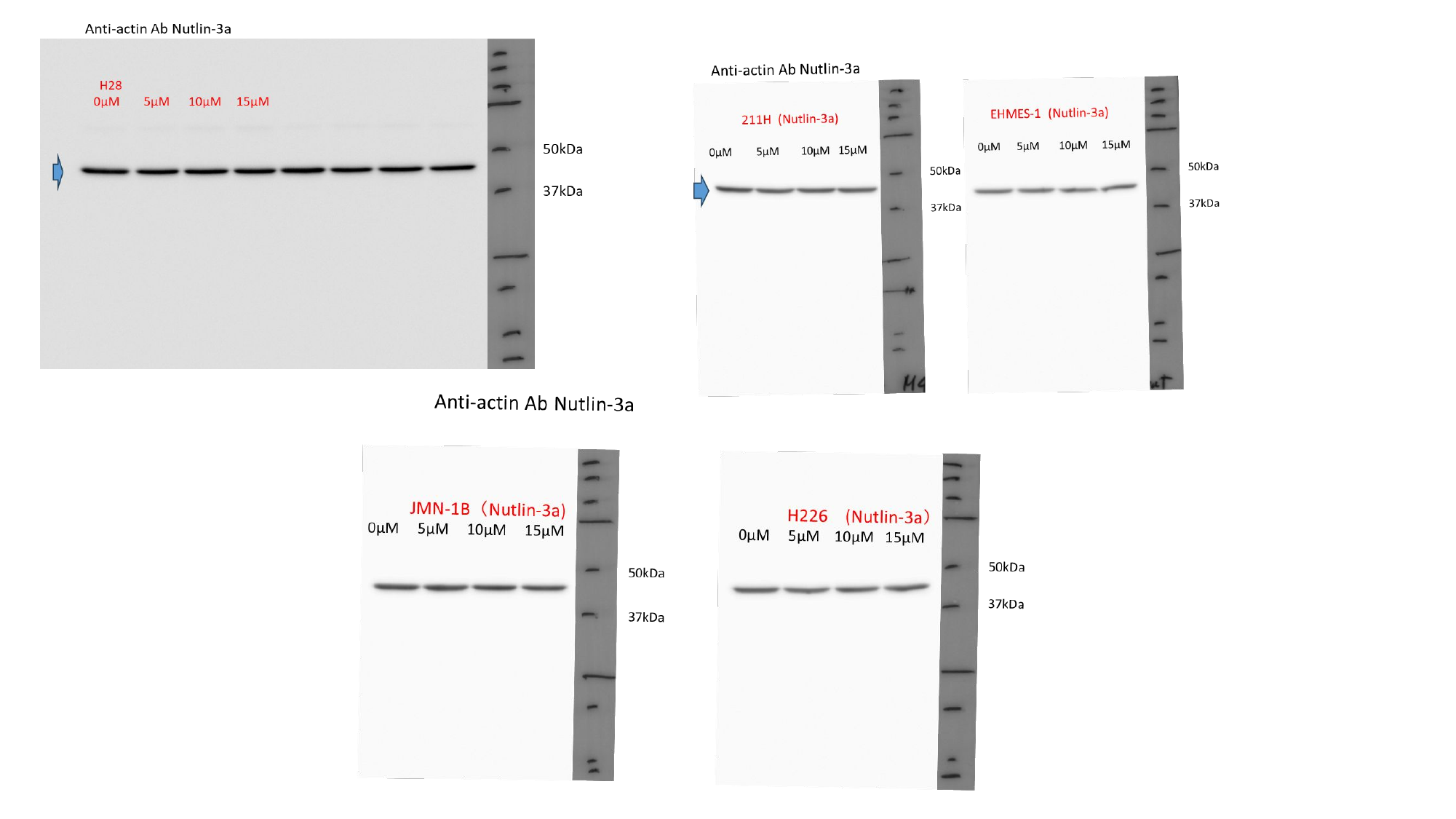

Supplement: S17 Fig — Original blots which were used for Fig 3B actin expression. Arrows indicate the target molecules. The name of cells was shown in the abbreviations. We did not use a photo of 15 μM nutlin-3a treatments. (PPTX) [file pone.0343551.s017.pptx]

## Slide 1
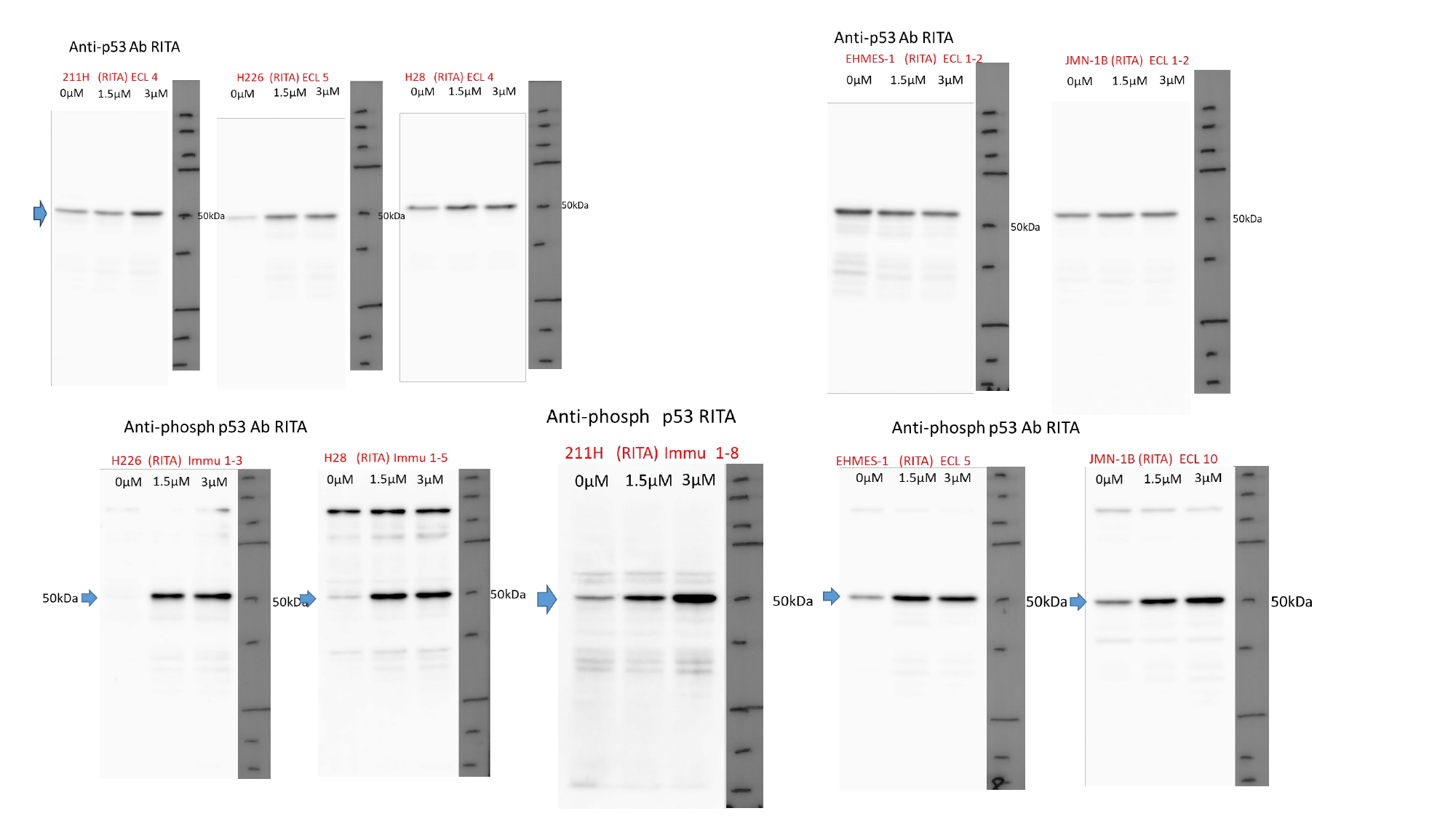

Supplement: S18 Fig — Original blots which were used for Fig 4B p53 and phosphorylated p53 expressions. Arrows indicate the target molecules. The name of cells was shown in the abbreviations. In some of the blots, we used the same blot to detect others molecules without stripping the blot and consequently showed the target molecules by the arrows. (PPTX) [file pone.0343551.s018.pptx]

## Slide 1
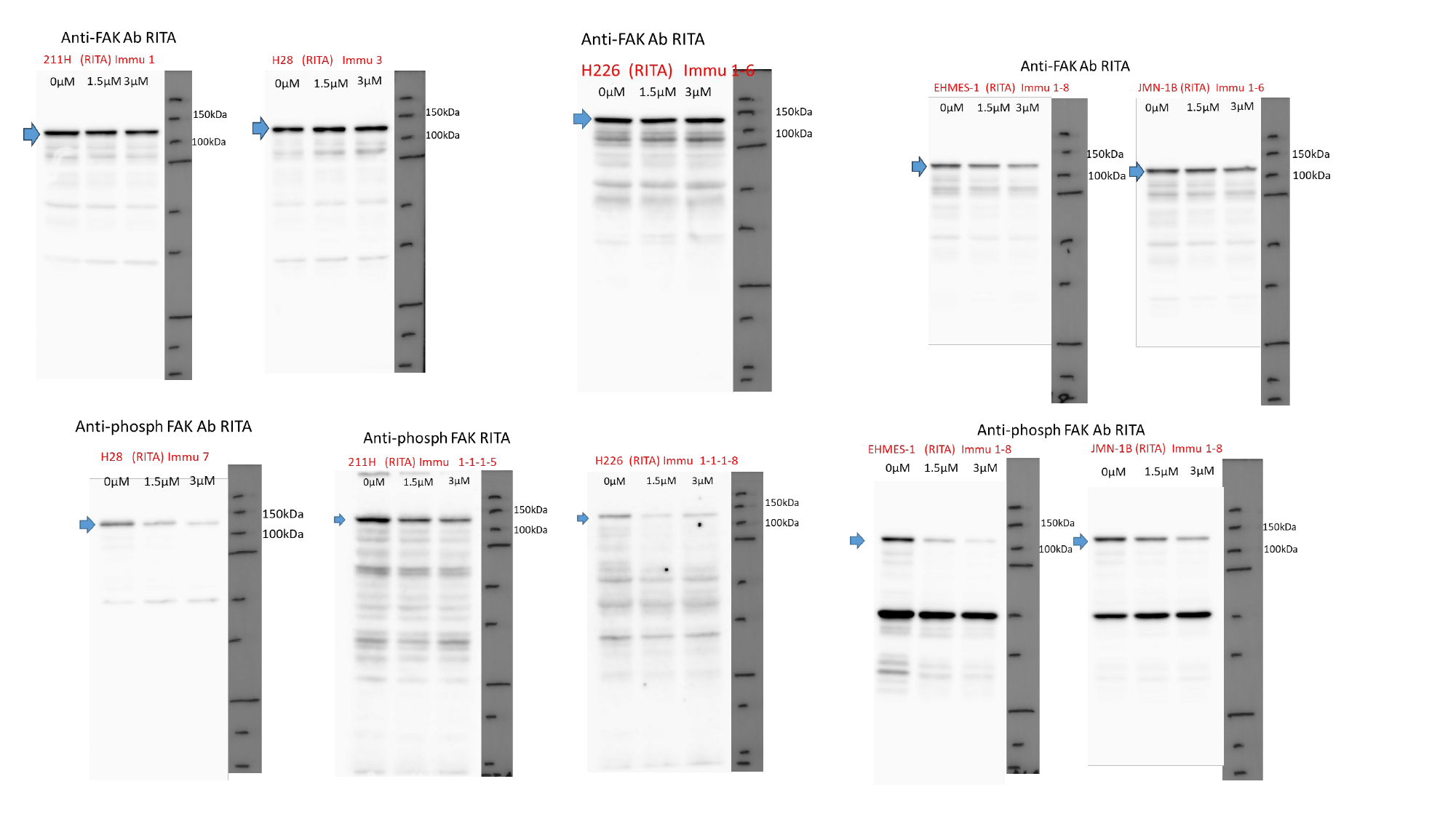

Supplement: S19 Fig — Original blots which were used for Fig 4B FAK and phosphorylated FAK expressions. Arrows indicate the target molecules. The name of cells was shown in the abbreviations. In some of the blots, we used the same blot to detect others molecules without stripping the blot and consequently showed the target molecules by the arrows. (PPTX) [file pone.0343551.s019.pptx]

## Slide 1
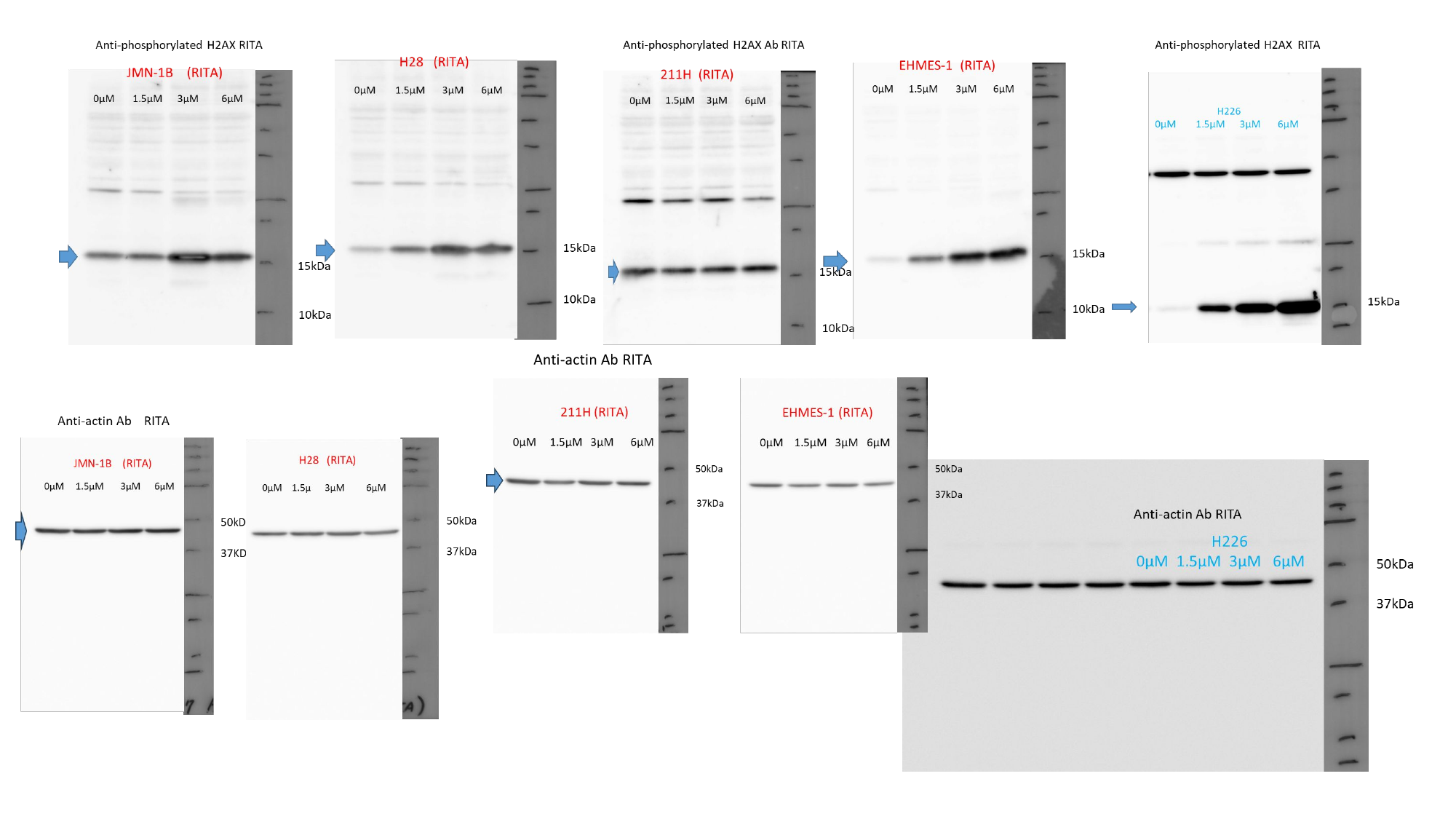

Supplement: S20 Fig — Original blots which were used for Fig 4B p-H2AX and actin expressions. Arrows indicate the target molecules. The name of cells was shown in the abbreviations. We did not use a photo of 6 μM RITA treatments. In some of the blots, we used the same blot to detect others molecules without stripping the blot and consequently showed the target molecules by the arrows. (PPTX) [file pone.0343551.s020.pptx]

## Slide 1
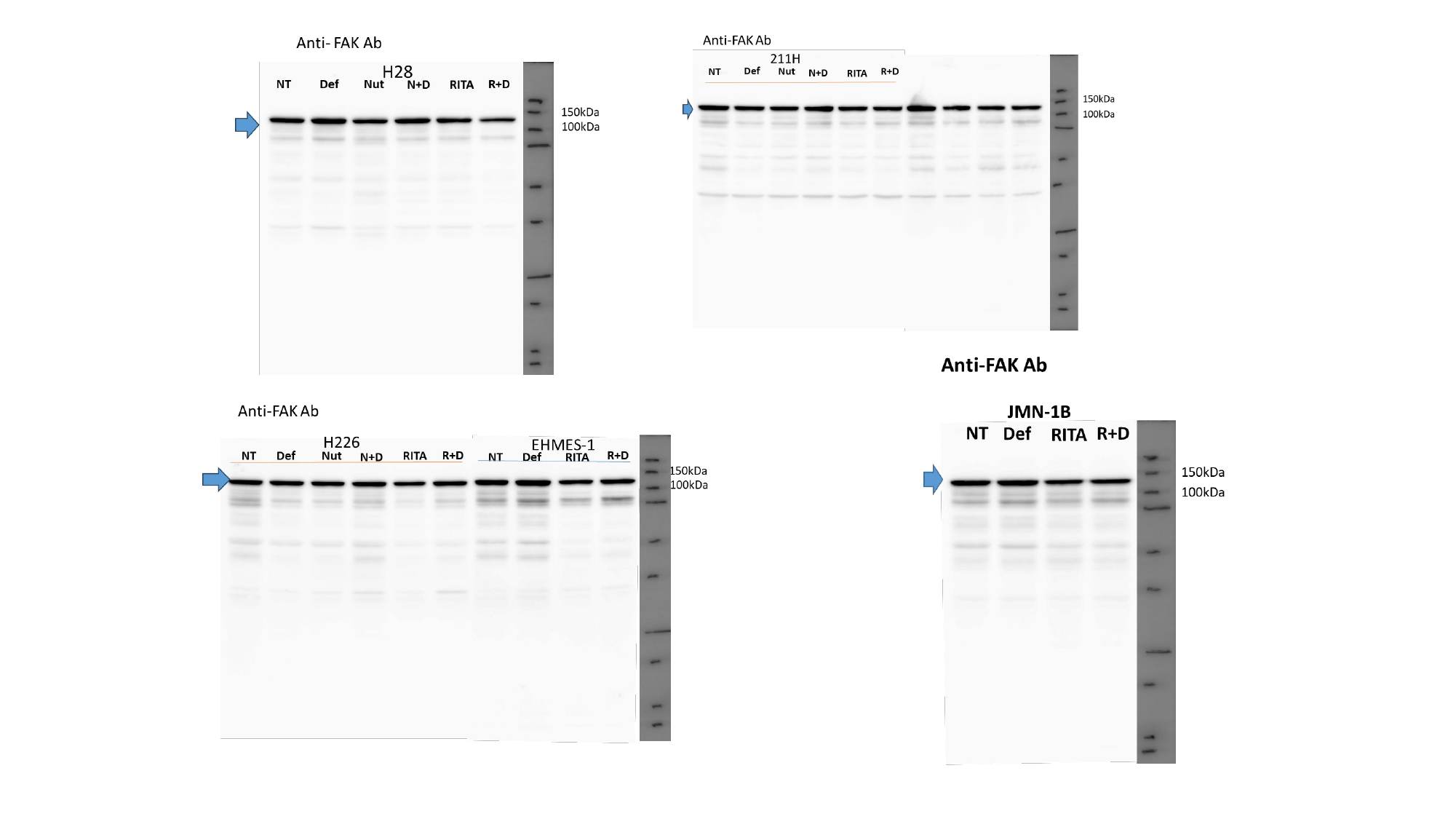

Supplement: S21 Fig — Original blots which were used for Fig 7 FAK expression. Arrows indicate the target molecules. The name of cells was shown in the abbreviations. Abbreviations. NT: no treatment shown as (-) in Fig 7, Def: defactinib, Nut: nutlin-3a, N + D: nutlin-3a+defactinib, R + D: RITA+defactinib. (PPTX) [file pone.0343551.s021.pptx]

## Slide 1
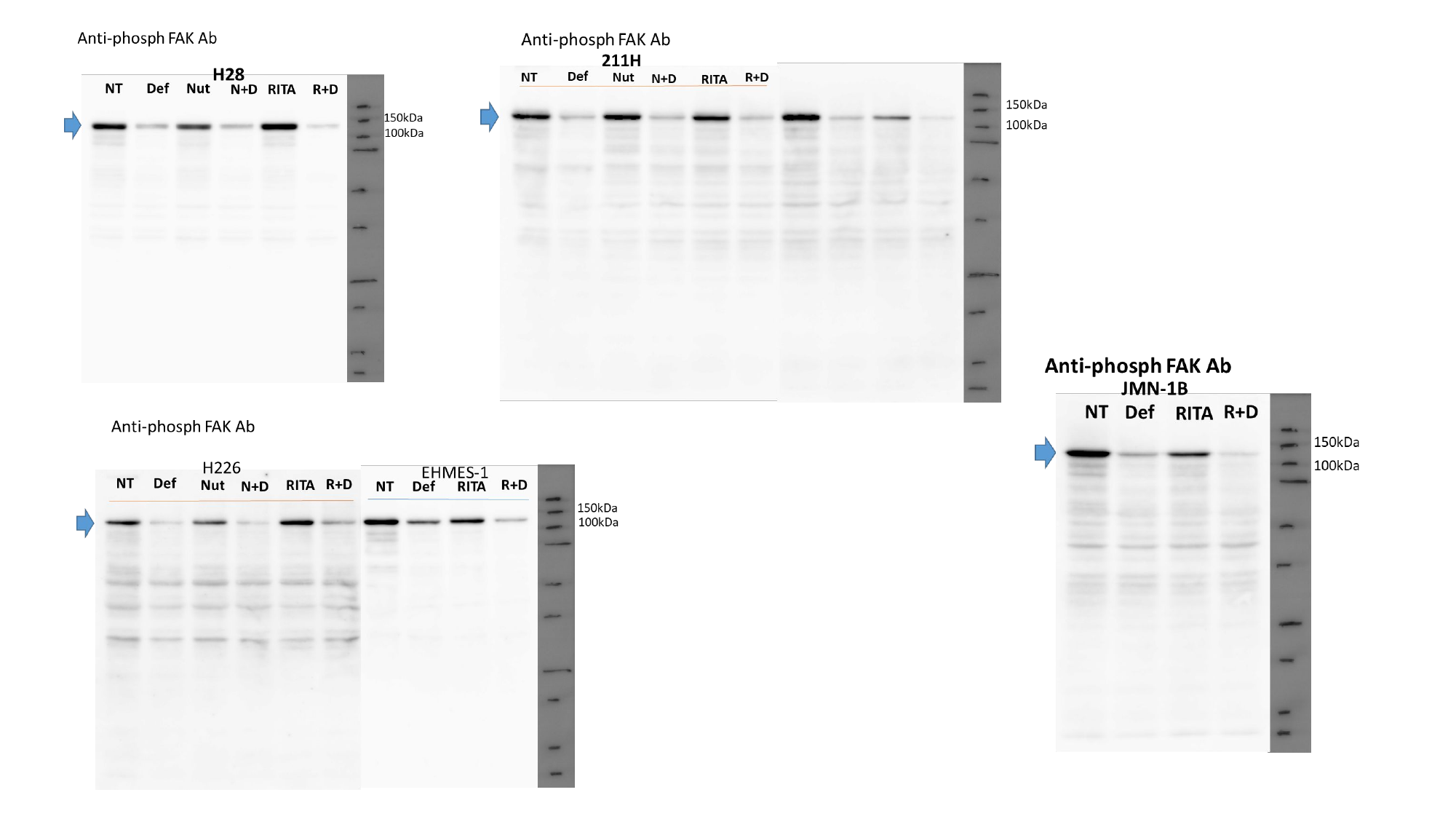

Supplement: S22 Fig — Original blots which were used for Fig 7 phosphorylated FAK expression. Arrows indicate the target molecules. The name of cells was shown in the abbreviations. Abbreviations used for treatment were shown in S21 Fig. (PPTX) [file pone.0343551.s022.pptx]

## Slide 1
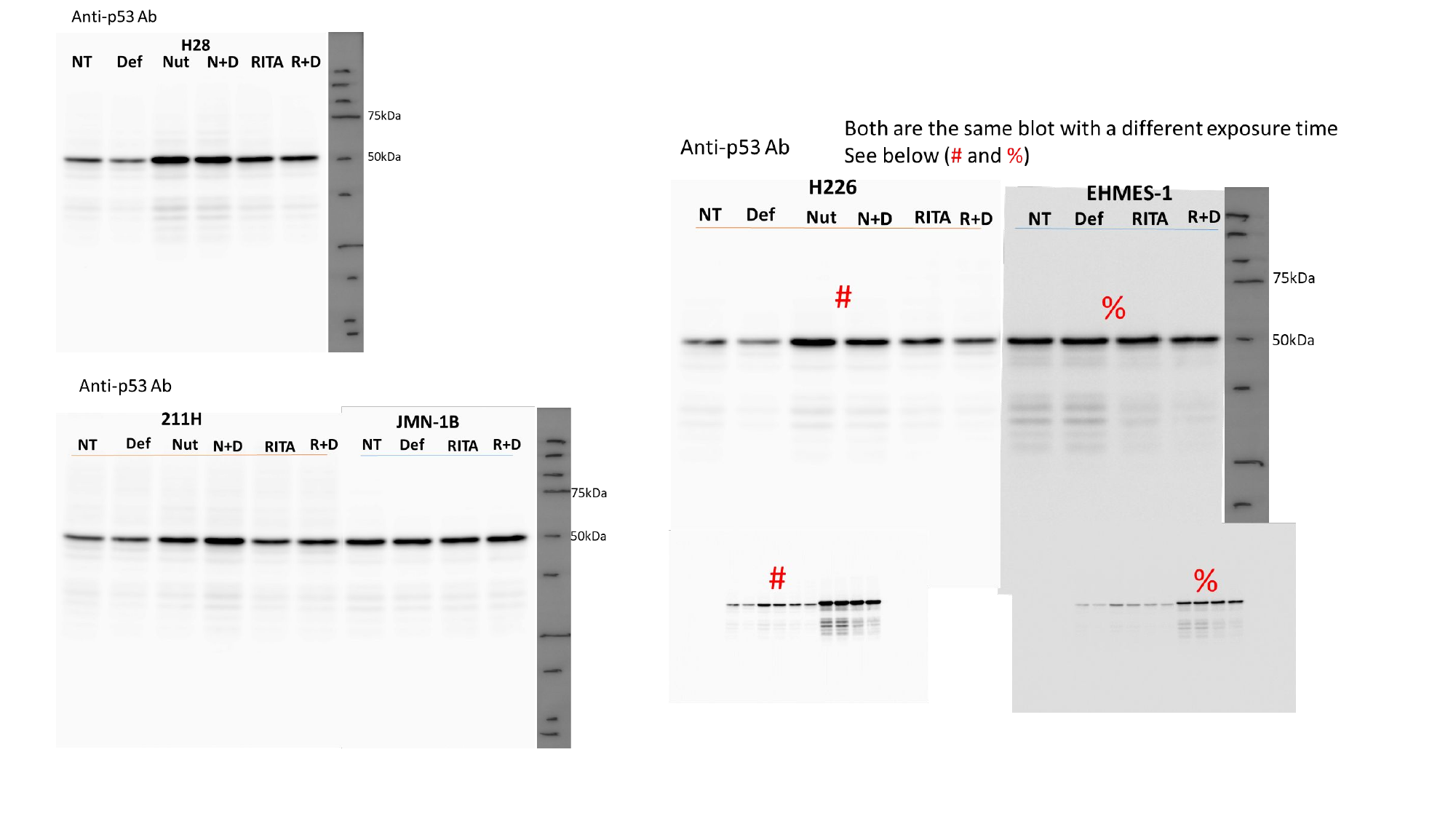

Supplement: S23 Fig — Original blots which were used for Fig 7 p53 expression. Arrows indicate the target molecules. The name of cells was shown in the abbreviations. Abbreviations used for treatment were shown in S21 Fig. (PPTX) [file pone.0343551.s023.pptx]

## Slide 1
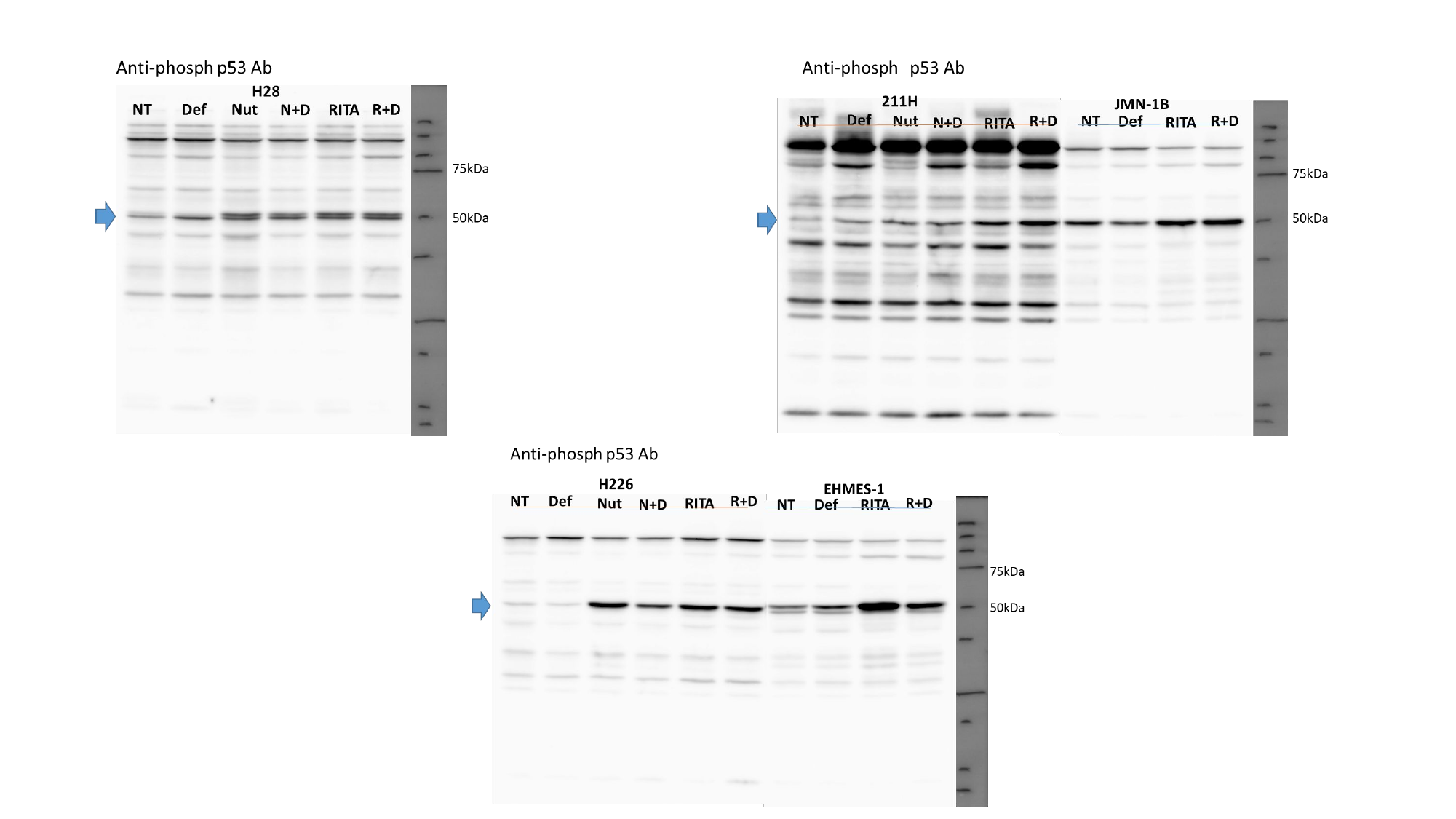

Supplement: S24 Fig — Original blots which were used for Fig 7 phosphorylated p53 expression. Arrows indicate the target molecules. The name of cells was shown in the abbreviations. Abbreviations used for treatment were shown in S21 Fig. In some of the blots, we used the same blot to detect others molecules without stripping the blot and consequently showed the target molecules by the arrows. (PPTX) [file pone.0343551.s024.pptx]

## Slide 1
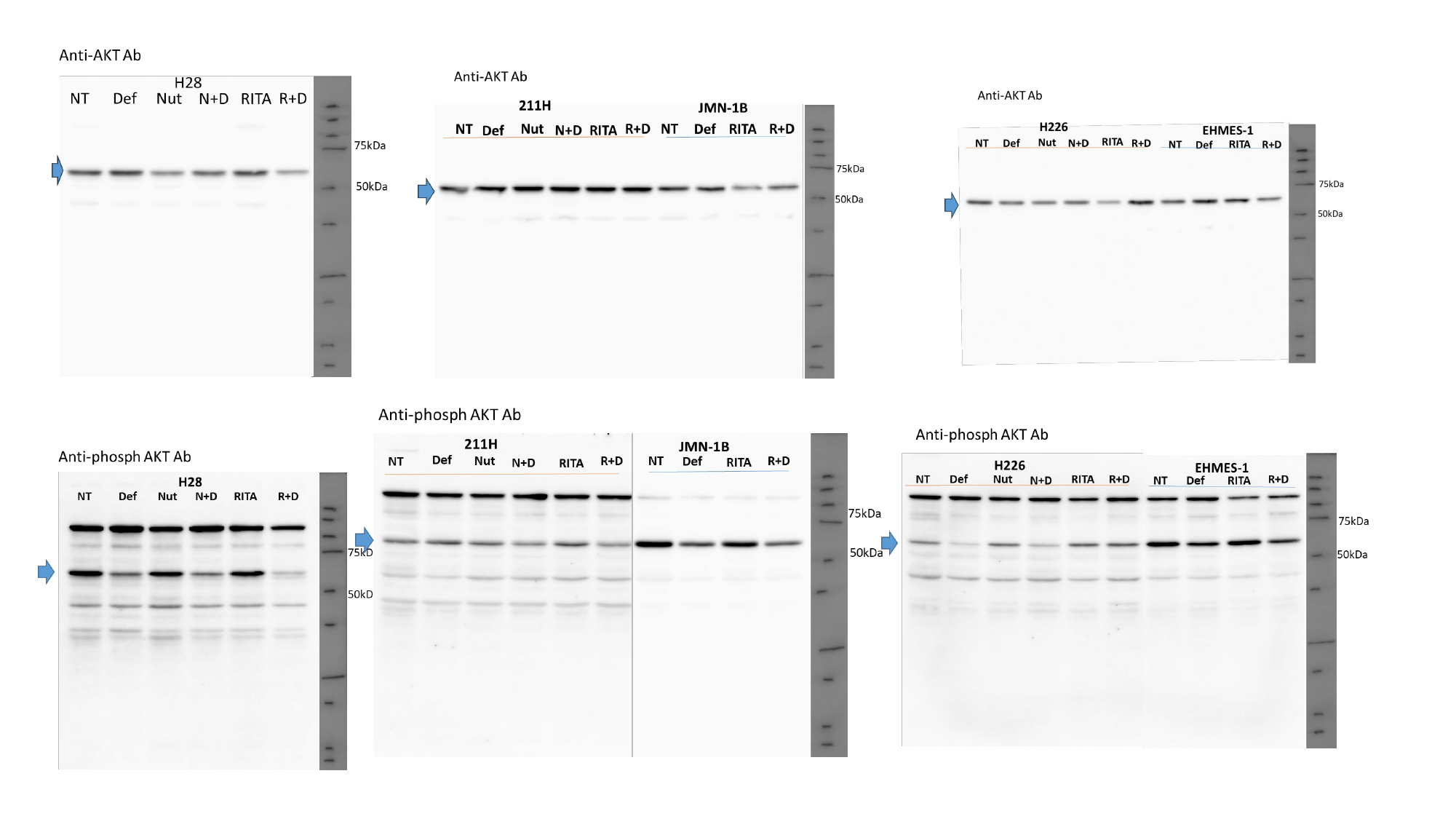

Supplement: S25 Fig — Original blots which were used for Fig 7 AKT and phosphorylated AKT expressions. Arrows indicate the target molecules. The name of cells was shown in the abbreviations. Abbreviations used for treatment were shown in S21 Fig. In some of the blots, we used the same blot to detect others molecules without stripping the blot and consequently showed the target molecules by the arrows. (PPTX) [file pone.0343551.s025.pptx]

## Slide 1
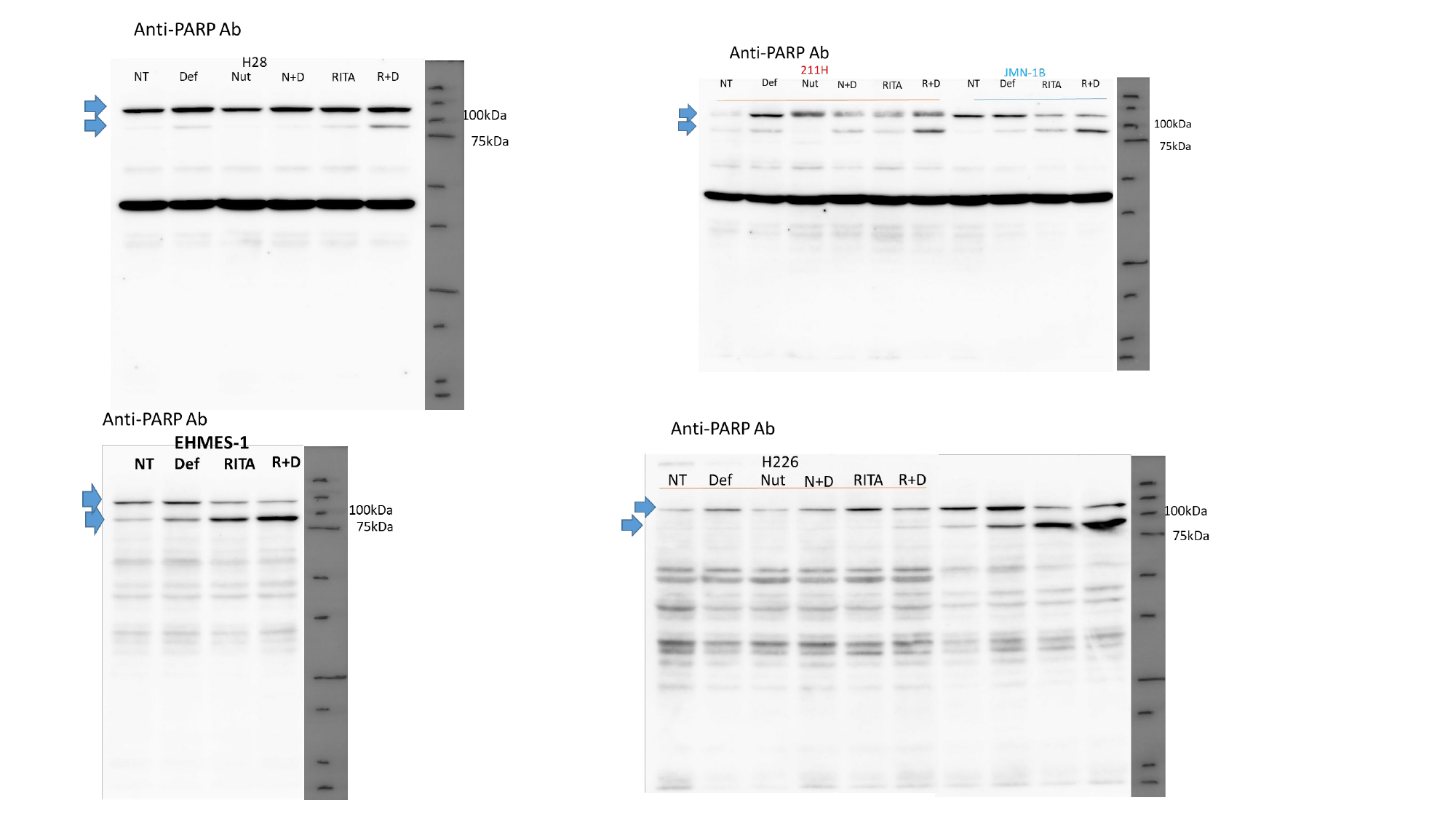

Supplement: S26 Fig — Original blots which were used for Fig 7 PARP and cleaved PARP expressions. The antibody detected the cleaved form. Arrows indicate the target molecules (both original and cleaved molecules). The name of cells was shown in the abbreviations. Abbreviations used for treatment were shown in S21 Fig. In some of the blots, we used the same blot to detect others molecules without stripping the blot and consequently showed the target molecules by the arrows. (PPTX) [file pone.0343551.s026.pptx]

## Slide 1
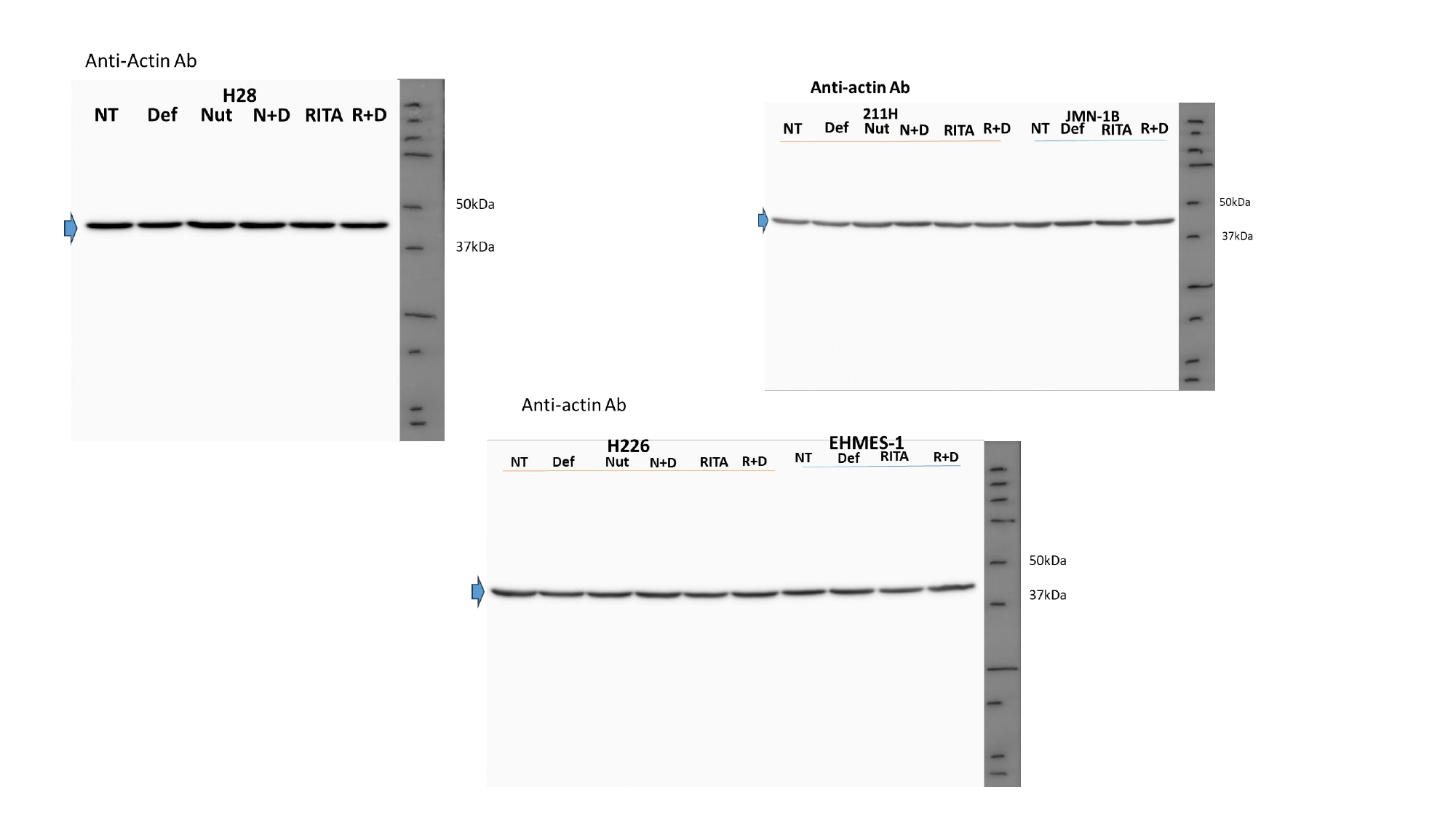

Supplement: S27 Fig — Original blots which were used for Fig 7 actin expression. Arrows indicate the target molecules. The name of cells was shown in the abbreviations. Abbreviations used for treatment were shown in S21 Fig. (PPTX) [file pone.0343551.s027.pptx]
