# Supplementary material for: Strategic selection of MDM2 inhibitors enhances the efficacy of FAK inhibition in mesothelioma based on TP53 genotype
Source: PLoS One. 2026 Feb 23;21(2):e0343551. doi: 10.1371/journal.pone.0343551 (PMC12928570; doi:10.1371/journal.pone.0343551)
Supplement: S2 Fig — Original blots which were used for Fig 1. Arrows indicate the target molecules. The name of cells was shown in abbreviations. H28: NCI-H28, 211H: MSTO-211H, H2052: NCI-H2052, H226: NCI-H226, H2452: NCI-H2452. EH-10: EHMES-10, EH-1: EHMES-1. (PDF) [file pone.0343551.s002.pdf]

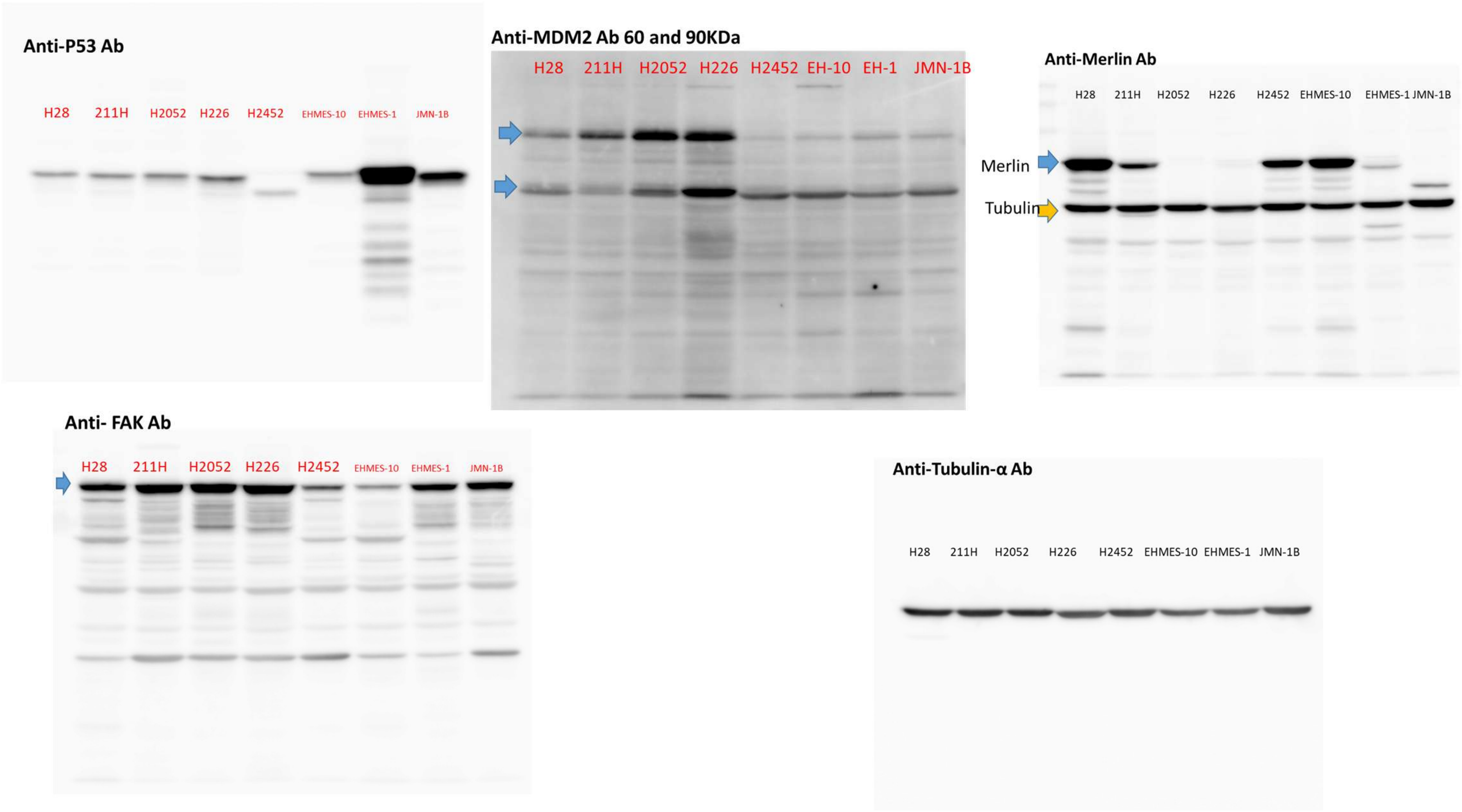
Anti-P53 Ab
H28 211H H2052 H226 H2452 EHMES-10 EHMES-1 JMN-1B
Anti-MDM2 Ab 60 and 90KDa
H28 211H H2052 H226 H2452 EH-10 EH-1 JMN-1B
Anti-Merlin Ab
H28 211H H2052 H226 H2452 EHMES-10 EHMES-1 JMN-1B
Merlin
Tubulin
Anti- FAK Ab
H28 211H H2052 H226 H2452 EHMES-10 EHMES-1 JMN-1B
Anti-Tubulin-α Ab
H28 211H H2052 H226 H2452 EHMES-10 EHMES-1 JMN-1B
