# Supplementary material for: Strategic selection of MDM2 inhibitors enhances the efficacy of FAK inhibition in mesothelioma based on TP53 genotype
Source: PLoS One. 2026 Feb 23;21(2):e0343551. doi: 10.1371/journal.pone.0343551 (PMC12928570; doi:10.1371/journal.pone.0343551)
Supplement: S1 Table — (DOCX) [file pone.0343551.s028.docx]

Supplementary Table 1

| (1) | NCI-H28 | MSTO-211H | NCI-H2052 | NCI-H226 | NCI-H2452 |
| --- | --- | --- | --- | --- | --- |
| Histology | Epitheloid | Biphasic | Sarcomatoid | Epitheloid | Epitheloid |
| ATCC-ID | HTB-128 | CRL-2081 | CRL-5915 | CRL-5826 | CRL-5946 |
| RRID | CVCL_1275 | CVCL_1430 | CVCL_1500 | CVCL_1545 | CVCL_1558 |
| *TP53* genotype | Wild-type | Wild-type | Wild-type | Wild-type | Wild-type |
| Protein level | Wild-type | Wild-type | Wild-type | Wild-type | Truncated |

| (2) | EHMES-10 | EHMES-1 | JMN-1B | Met-5A |
| --- | --- | --- | --- | --- |
| Histology | Unknown | Unknown | Biphasic | Pleural mesothelium |
| ATCC-ID | None | None | None | CRL-9444 |
| RRID | None | None | None | CVCL_3740 |
| *TP53* genotype | Wild-type | Mutated  (R273S) | Mutated  (G245S) | Wild-type |
| Protein level | Wild-type | Wild-type | Wild-type | Wild-type |
