# Supplementary material for: Strategic selection of MDM2 inhibitors enhances the efficacy of FAK inhibition in mesothelioma based on TP53 genotype
Source: PLoS One. 2026 Feb 23;21(2):e0343551. doi: 10.1371/journal.pone.0343551 (PMC12928570; doi:10.1371/journal.pone.0343551)
Supplement: S2 Table — Dilution of primary antibody and information of secondary antibody used in the study. (DOCX) [file pone.0343551.s029.docx]

Supplementary Table 2

| Antibody | Dilution | Secondary antibody |
| --- | --- | --- |
| Merlin | 1:1000 | HRP-linked ant-rabbit IgG  (#561, Cell Signaling) |
| FAK | 1:1000 | HRP-linked ant-rabbit IgG  (#561, Cell Signaling) |
| phosphorylated FAK | 1:1000 | HRP-linked ant-rabbit IgG  (#561, Cell Signaling) |
| p53 | 1:3000 | HRP-linked anti-mouse IgG  (#564, Cell Signaling) |
| Phosphorylated p53 | 1:1000 | HRP-linked anti-rabbit IgG  (#561, Cell Signaling) |
| AKT | 1:1000 | HRP-linked anti-rabbit IgG  (#559, Cell Signaling) |
| Phosphorylated AKT | 1:1000 | HRP-linked anti-rabbit IgG  (#559, Cell Signaling) |
| MDM2 | 1:200 | HRP-linked anti-mouse IgG  (#564, Cell Signaling) |
| Phosphorylated MDM2 | 1:1000 | HRP-linked anti-rabbit IgG  (#596, Cell Signaling) |
| Capase-9 | 1:1000 | HRP-linked anti-rabbit IgG  (#491, Cell Signaling) |
| PARP | 1:1000 | HRP-linked anti-rabbit IgG  (#596, Cell Signaling) |
| Tubulin | 1:3000 | HRP-linked anti-mouse IgG  (#564, Cell Signaling) |
| Phosphorylated H2AX | 1:500 | HRP-linked anti-mouse IgG  (#564, Cell Signaling) |
| Actin | 1:1000 | HRP-linked anti-rabbit IgG  (#617, Cell Signaling) |
