# Supplementary material for: Strategic selection of MDM2 inhibitors enhances the efficacy of FAK inhibition in mesothelioma based on TP53 genotype
Source: PLoS One. 2026 Feb 23;21(2):e0343551. doi: 10.1371/journal.pone.0343551 (PMC12928570; doi:10.1371/journal.pone.0343551)
Supplement: S3 Table — Expression of the molecules in Fig 1 was quantified with ImageJ software (NIH, Bethesda, MD, USA). The intensity of target protein bands was normalized to the intensity of tubulin as a loading control. Respective protein expression levels of NCI-H28 were used as a standard (expressed as 1.00). (DOCX) [file pone.0343551.s030.docx]

Supplementary Table 3 (for Figure 1)

|  | NCI-H28 | MSTO-211H | NCI-H2052 | NCI-H226 | NCI-H2452 | EHMES-10 | EHMES-1 | JMN-1B |
| --- | --- | --- | --- | --- | --- | --- | --- | --- |
| p53 | 1.00 | 1.35 | 1.82 | 2.60 | 0.70 | 2.84 | 21.51 | 6.61 |
| MDM2  90 kDa | 1.00 | 1.77 | 2.90 | 3.34 | 0.48 | 0.57 | 0.82 | 0.49 |
| MDM2  60 kDa | 1.00 | 0.93 | 1.65 | 3.32 | 1.72 | 2.34 | 1.82 | 1.33 |
| MERLIN | 1.00 | 0.23 | 0.01 | 0.02 | 0.55 | 1.15 | 0.10 | <0.01 |
| FAK | 1.00 | 1.43 | 1.54 | 1.76 | 0.53 | 0.42 | 1.51 | 1.22 |
