# Supplementary material for: Strategic selection of MDM2 inhibitors enhances the efficacy of FAK inhibition in mesothelioma based on TP53 genotype
Source: PLoS One. 2026 Feb 23;21(2):e0343551. doi: 10.1371/journal.pone.0343551 (PMC12928570; doi:10.1371/journal.pone.0343551)
Supplement: S4 Table — Expression of the molecules in Fig 2B was quantified with ImageJ software (NIH, Bethesda, MD, USA). The intensity of target protein bands was normalized to the intensity of tubulin as a loading control. Respective protein expression levels of untreated cells were used as a standard (expressed as 1.00). (DOCX) [file pone.0343551.s031.docx]

Supplementary Table 4 (for Figure 2B)

| (1) | NCI-H28 | | | | MSTO-211H | | | |
| --- | --- | --- | --- | --- | --- | --- | --- | --- |
| Defactinib (μM) | (-) | 1 | 2 | 4 | (-) | 1 | 2 | 4 |
| FAK | 1.00 | 1.24 | 1.20 | 1.08 | 1.00 | 0.84 | 0.79 | 0.68 |
| P-FAK | 1.00 | 0.69 | 0.50 | 0.40 | 1.00 | 0.14 | 0.15 | 0.15 |
| p53 | 1.00 | 3.00 | 2.89 | 3.76 | 1.00 | 0.67 | 0.84 | 1.79 |
| P-p53 | 1.00 | 1.38 | 1.50 | 1.52 | 1.00 | 0.63 | 0.64 | 1.22 |
| AKT | 1.00 | 0.92 | 0.80 | 0.80 | 1.00 | 0.80 | 0.87 | 0.89 |
| p-AKT | 1.00 | 0.98 | 0.75 | 0.72 | 1.00 | 0.83 | 0.54 | 0.50 |
| MDM2  90 kDa | 1.00 | 0.91 | 0.85 | 1.00 | 1.00 | 0.73 | 0.90 | 1.16 |
| MDM2  60 kDa | 1.00 | 0.95 | 1.00 | 1.06 | 1.00 | 0.71 | 0.71 | 0.57 |
| P-MDM2  90 kDa | 1.00 | 1.55 | 1.61 | 2.11 | 1.00 | 0.64 | 0.68 | 0.87 |
| Caspase-9 | 1.00 | 1.27 | 1.40 | 1.54 | 1.00 | 0.86 | 0.87 | 1.08 |
| Cleaved-Caapase-9 | 1.00 | 1.18 | 1.33 | 1.51 | 1.00 | 0.79 | 0.76 | 0.84 |
| PARP | 1.00 | 1.51 | 1.16 | 1.52 | 1.00 | 1.01 | 0.83 | 1.13 |
| Cleaved  PARP | 1.00 | 1.52 | 1.45 | 1.55 | 1.00 | 0.94 | 0.72 | 1.38 |

| (2) | NCI-H2052 | | | | NCI-H226 | | | |
| --- | --- | --- | --- | --- | --- | --- | --- | --- |
| Defactinib (μM) | (-) | 1 | 2 | 4 | (-) | 1 | 2 | 4 |
| FAK | 1.00 | 0.97 | 0.83 | 0.84 | 1.00 | 1.02 | 0.89 | 0.65 |
| P-FAK | 1.00 | 0.47 | 0.20 | 0.15 | 1.00 | 0.23 | 0.28 | 0.26 |
| p53 | 1.00 | 1.05 | 1.88 | 3.35 | 1.00 | 0.92 | 0.83 | 0.39 |
| P-p53 | 1.00 | 0.56 | 0.64 | 0.83 | 1.00 | 0.66 | 0.63 | 0.48 |
| AKT | 1.00 | 0.78 | 0.69 | 0.68 | 1.00 | 0.90 | 0.78 | 0.54 |
| p-AKT | 1.00 | 0.61 | 0.49 | 0.49 | 1.00 | 0.85 | 0.74 | 0.34 |
| MDM2  90 kDa | 1.00 | 0.79 | 0.83 | 0.86 | 1.00 | 0.83 | 0.73 | 0.79 |
| MDM2  60 kDa | 1.00 | 0.74 | 0.89 | 0.95 | 1.00 | 0.85 | 0.70 | 0.77 |
| P-MDM2  90 kDa | 1.00 | 0.46 | 0.60 | 0.90 | 1.00 | 0.74 | 0.66 | 0.92 |
| Caspase-9 | 1.00 | 0.86 | 0.90 | 1.01 | 1.00 | 1.08 | 0.98 | 0.74 |
| Cleaved-Caapase-9 | 1.00 | 1.18 | 1.22 | 1.21 | 1.00 | 0.97 | 0.91 | 0.41 |
| PARP | 1.00 | 0.79 | 0.76 | 0.88 | 1.00 | 0.85 | 0.84 | 0.50 |
| Cleaved  PARP | 1.00 | 0.69 | 0.84 | 0.79 | 1.00 | 1.01 | 0.99 | 0.32 |

| (3) | NCI-H2452 | | | | EHMES-10 | | | |
| --- | --- | --- | --- | --- | --- | --- | --- | --- |
| Defactinib (μM) | (-) | 1 | 2 | 4 | (-) | 1 | 2 | 4 |
| FAK | 1.00 | 1.17 | 1.16 | 1.02 | 1.00 | 0.69 | 0.70 | 0.71 |
| P-FAK | 1.00 | 0.61 | 0.70 | 0.74 | 1.00 | 0.66 | 0.45 | 0.51 |
| p53 | 1.00 | 1.09 | 1.41 | 1.20 | 1.00 | 1.42 | 1.05 | 1.78 |
| P-p53 | 1.00 | 1.07 | 1.23 | 1.38 | 1.00 | 0.93 | 0.57 | 0.94 |
| AKT | 1.00 | 0.98 | 0.94 | 0.75 | 1.00 | 0.93 | 0.77 | 0.90 |
| p-AKT | 1.00 | 0.94 | 0.85 | 0.54 | 1.00 | 0.75 | 0.58 | 0.40 |
| MDM2  90 kDa | 1.00 | 1.41 | 1.20 | 1.14 | 1.00 | 1.04 | 0.80 | 0.94 |
| MDM2  60 kDa | 1.00 | 0.85 | 0.93 | 0.79 | 1.00 | 1.00 | 0.75 | 0.75 |
| P-MDM2  90 kDa | 1.00 | 0.71 | 0.74 | 0.78 | 1.00 | 0.88 | 0.38 | 0.56 |
| Caspase-9 | 1.00 | 0.91 | 0.86 | 0.71 | 1.00 | 0.87 | 0.96 | 0.91 |
| Cleaved-Caapase-9 | 1.00 | 0.91 | 0.96 | 0.72 | 1.00 | 0.70 | 0.99 | 0.77 |
| PARP | 1.00 | 0.86 | 0.89 | 0.66 | 1.00 | 1.03 | 0.55 | 0.95 |
| Cleaved  PARP | 1.00 | 0.70 | 0.80 | 0.60 | 1.00 | 1.18 | 0.59 | 1.16 |

| (4) | EHMES-1 | | | | JMN-1B | | | |
| --- | --- | --- | --- | --- | --- | --- | --- | --- |
| Defactinib (μM) | (-) | 1 | 2 | 4 | (-) | 1 | 2 | 4 |
| FAK | 1.00 | 0.94 | 1.01 | 0.98 | 1.00 | 1.20 | 1.09 | 1.03 |
| P-FAK | 1.00 | 0.56 | 0.57 | 0.50 | 1.00 | 0.19 | 0.15 | 0.10 |
| p53 | 1.00 | 1.12 | 1.17 | 1.28 | 1.00 | 0.98 | 0.80 | 0.74 |
| P-p53 | 1.00 | 0.82 | 1.13 | 1.42 | 1.00 | 0.96 | 1.04 | 0.90 |
| AKT | 1.00 | 0.99 | 1.05 | 1.07 | 1.00 | 1.14 | 0.92 | 0.88 |
| p-AKT | 1.00 | 1.31 | 0.87 | 0.84 | 1.00 | 0.82 | 0.59 | 0.54 |
| MDM2  90 kDa | 1.00 | 1.07 | 1.25 | 1.33 | 1.00 | 1.03 | 1.22 | 0.88 |
| MDM2  60 kDa | 1.00 | 1.02 | 1.12 | 1.28 | 1.00 | 0.96 | 0.80 | 0.83 |
| P-MDM2  90 kDa | 1.00 | 1.04 | 1.11 | 2.11 | 1.00 | 0.86 | 0.71 | 0.66 |
| Caspase-9 | 1.00 | 0.86 | 0.99 | 1.45 | 1.00 | 1.22 | 0.97 | 0.73 |
| Cleaved-Caapase-9 | 1.00 | 0.97 | 0.91 | 0.79 | 1.00 | 1.23 | 1.21 | 1.07 |
| PARP | 1.00 | 1.37 | 1.52 | 1.43 | 1.00 | 0.91 | 0.66 | 0.72 |
| Cleaved  PARP | 1.00 | 1.10 | 1.56 | 1.75 | 1.00 | 0.91 | 0.57 | 0.69 |
