# Supplementary material for: Strategic selection of MDM2 inhibitors enhances the efficacy of FAK inhibition in mesothelioma based on TP53 genotype
Source: PLoS One. 2026 Feb 23;21(2):e0343551. doi: 10.1371/journal.pone.0343551 (PMC12928570; doi:10.1371/journal.pone.0343551)
Supplement: S5 Table — Expression of the molecules in Fig 3B was quantified with ImageJ software (NIH, Bethesda, MD, USA). The intensity of target protein bands was normalized to the intensity of actin as a loading control. Respective protein expression levels of untreated cells were used as a standard (expressed as 1.00). (DOCX) [file pone.0343551.s032.docx]

Supplementary Table 5 (for Figure 3B)

| (1) | NCI-H28 | | | MSTO-211H | | | NCI-H226 | | |
| --- | --- | --- | --- | --- | --- | --- | --- | --- | --- |
| Nultin-3a (μM) | (-) | 5 | 10 | (-) | 5 | 10 | (-) | 5 | 10 |
| p53 | 1.00 | 3.61 | 9.52 | 1.00 | 10.06 | 17.43 | 1.00 | 6.06 | 8.09 |
| P-p53 | 1.00 | 4.24 | 11.57 | 1.00 | 6.37 | 9.87 | 1.00 | 15.11 | 15.14 |
| FAK | 1.00 | 1.33 | 1.48 | 1.00 | 1.26 | 1.29 | 1.00 | 1.51 | 1.30 |
| P-FAK | 1.00 | 1.27 | 1.03 | 1.00 | 0.28 | 0.22 | 1.00 | 0.47 | 0.21 |
| P-H2AX | 1.00 | 0.43 | 0.63 | 1.00 | 1.14 | 1.69 | 1.00 | 0.34 | 1.45 |

| (2) | EHMES-1 | | | JMN-1B | | |
| --- | --- | --- | --- | --- | --- | --- |
| Nultin-3a (μM) | (-) | 5 | 10 | (-) | 5 | 10 |
| p53 | 1.00 | 1.31 | 1.21 | 1.00 | 1.65 | 1.62 |
| P-p53 | 1.00 | 1.70 | 1.91 | 1.00 | 1.35 | 1.00 |
| FAK | 1.00 | 1.10 | 1.11 | 1.00 | 0.88 | 0.60 |
| P-FAK | 1.00 | 1.23 | 0.93 | 1.00 | 0.51 | 0.34 |
| P-H2AX | 1.00 | 1.10 | 0.84 | 1.00 | 1.02 | 1.27 |
