# Supplementary material for: Strategic selection of MDM2 inhibitors enhances the efficacy of FAK inhibition in mesothelioma based on TP53 genotype
Source: PLoS One. 2026 Feb 23;21(2):e0343551. doi: 10.1371/journal.pone.0343551 (PMC12928570; doi:10.1371/journal.pone.0343551)
Supplement: S6 Table — Expression of the molecules in Fig 4B was quantified with ImageJ software (NIH, Bethesda, MD, USA). The intensity of target protein bands was normalized to the intensity of actin as a loading control. Respective protein expression levels of untreated cells were used as a standard (expressed as 1.00). (DOCX) [file pone.0343551.s033.docx]

Supplementary Table 6 (for Figure 4B)

| (1) | NCI-H28 | | | MSTO-211H | | | NCI-H226 | | |
| --- | --- | --- | --- | --- | --- | --- | --- | --- | --- |
| RITA (μM) | (-) | 1.5 | 3 | (-) | 1.5 | 3 | (-) | 1.5 | 3 |
| p53 | 1.00 | 1.81 | 1.63 | 1.00 | 1.67 | 2.30 | 1.00 | 4.78 | 4.51 |
| P-p53 | 1.00 | 9.58 | 8.56 | 1.00 | 3.86 | 5.33 | 1.00 | 27.78 | 30.73 |
| FAK | 1.00 | 1.28 | 1.17 | 1.00 | 1.19 | 0.88 | 1.00 | 1.04 | 1.10 |
| P-FAK | 1.00 | 0.51 | 0.23 | 1.00 | 0.89 | 0.56 | 1.00 | 0.35 | 0.52 |
| P-H2AX | 1.00 | 2.98 | 5.07 | 1.00 | 1.27 | 1.31 | 1.00 | 26.14 | 41.78 |

| (2) | EHMES-1 | | | JMN-1B | | |
| --- | --- | --- | --- | --- | --- | --- |
| RITA (μM) | (-) | 1.5 | 3 | (-) | 1.5 | 3 |
| p53 | 1.00 | 1.03 | 0.78 | 1.00 | 1.15 | 1.18 |
| P-p53 | 1.00 | 4.52 | 3.48 | 1.00 | 2.01 | 2.42 |
| FAK | 1.00 | 1.04 | 0.64 | 1.00 | 0.82 | 0.65 |
| P-FAK | 1.00 | 0.29 | 0.09 | 1.00 | 0.62 | 0.35 |
| P-H2AX | 1.00 | 9.28 | 16.89 | 1.00 | 1.09 | 2.32 |
