# Supplementary material for: Strategic selection of MDM2 inhibitors enhances the efficacy of FAK inhibition in mesothelioma based on TP53 genotype
Source: PLoS One. 2026 Feb 23;21(2):e0343551. doi: 10.1371/journal.pone.0343551 (PMC12928570; doi:10.1371/journal.pone.0343551)
Supplement: S7 Table — Expression of the molecules in Fig 7 was quantified with ImageJ software (NIH, Bethesda, MD, USA). The intensity of target protein bands was normalized to the intensity of actin as a loading control. Respective protein expression levels of untreated cells were used as a standard (expressed as 1.00). (DOCX) [file pone.0343551.s034.docx]

Supplementary Table 7 (for Figure 7)

| (1) | NCI-H28 | | | | | | MSTO-211H | | | | | |
| --- | --- | --- | --- | --- | --- | --- | --- | --- | --- | --- | --- | --- |
| Treatment | (-) | Def | Nut | Nut+Def | RITA | RITA+Def | (-) | Def | Nut | Nut+Def | RITA | RITA+Def |
| FAK | 1.00 | 1.32 | 0.85 | 1.04 | 0.98 | 0.79 | 1.00 | 0.84 | 0.64 | 0.74 | 0.67 | 0.68 |
| P-FAK | 1.00 | 0.23 | 0.65 | 0.21 | 1.28 | 0.10 | 1.00 | 0.20 | 0.75 | 0.18 | 0.74 | 0.19 |
| p53 | 1.00 | 0.87 | 1.99 | 1.98 | 1.71 | 1.53 | 1.00 | 0.98 | 1.29 | 1.67 | 1.05 | 1.31 |
| P-p53 | 1.00 | 2.05 | 2.78 | 2.39 | 3.04 | 3.29 | 1.00 | 1.70 | 1.65 | 1.86 | 3.10 | 4.53 |
| AKT | 1.00 | 1.28 | 0.58 | 0.84 | 1.16 | 0.48 | 1.00 | 1.25 | 1.11 | 1.10 | 1.06 | 1.09 |
| P-AKT | 1.00 | 0.61 | 0.77 | 0.41 | 0.85 | 0.19 | 1.00 | 1.14 | 0.81 | 0.59 | 0.92 | 0.72 |
| PARP | 1.00 | 1.36 | 0.77 | 1.13 | 1.15 | 1.32 | 1.00 | 7.22 | 5.46 | 2.34 | 2.62 | 4.09 |
| Cleaved PARP | 1.00 | 2.12 | 0.58 | 0.96 | 2.00 | 6.48 | 1.00 | 1.94 | 0.53 | 1.31 | 1.06 | 4.55 |

| (2) | NCI-H226 | | | | | |
| --- | --- | --- | --- | --- | --- | --- |
| Treatment | (-) | Def | Nut | Nut+Def | RITA | RITA+Def |
| FAK | 1.00 | 1.16 | 1.04 | 1.05 | 0.94 | 1.02 |
| P-FAK | 1.00 | 0.11 | 0.81 | 0.11 | 1.65 | 0.45 |
| p53 | 1.00 | 0.90 | 2.59 | 2.00 | 1.78 | 1.34 |
| P-p53 | 1.00 | 0.87 | 10.07 | 5.60 | 9.99 | 8.64 |
| AKT | 1.00 | 0.92 | 0.78 | 0.84 | 0.51 | 1.35 |
| P-AKT | 1.00 | 0.44 | 1.06 | 0.51 | 1.21 | 1.27 |
| PARP | 1.00 | 3.32 | 1.16 | 2.40 | 5.40 | 2.62 |
| Cleaved PARP | 1.00 | 1.15 | 0.82 | 0.88 | 0.86 | 1.31 |

| (3) | EHMES-1 | | | | JMN-1B | | | |
| --- | --- | --- | --- | --- | --- | --- | --- | --- |
| Treatment | (-) | Def | RITA | RITA+Def | (-) | Def | RITA | RITA+Def |
| FAK | 1.00 | 1.17 | 1.01 | 0.92 | 1.00 | 1.08 | 0.92 | 0.93 |
| P-FAK | 1.00 | 0.43 | 0.87 | 0.16 | 1.00 | 0.25 | 0.54 | 0.11 |
| p53 | 1.00 | 1.09 | 1.26 | 0.98 | 1.00 | 0.97 | 0.98 | 1.01 |
| P-p53 | 1.00 | 1.43 | 3.60 | 2.04 | 1.00 | 0.72 | 1.41 | 1.53 |
| AKT | 1.00 | 1.46 | 1.54 | 0.79 | 1.00 | 0.92 | 0.56 | 0.73 |
| P-AKT | 1.00 | 0.79 | 1.22 | 0.64 | 1.00 | 0.52 | 0.80 | 0.44 |
| PARP | 1.00 | 1.24 | 0.82 | 0.59 | 1.00 | 1.02 | 0.44 | 0.44 |
| Cleaved PARP | 1.00 | 1.80 | 3.78 | 4.28 | 1.00 | 2.96 | 7.09 | 21.10 |
