# Supplementary material for: Strategic selection of MDM2 inhibitors enhances the efficacy of FAK inhibition in mesothelioma based on TP53 genotype
Source: PLoS One. 2026 Feb 23;21(2):e0343551. doi: 10.1371/journal.pone.0343551 (PMC12928570; doi:10.1371/journal.pone.0343551)
Supplement: Attachment — Submitted filename: Comments for reviewers 2025-11-5.docx [file pone.0343551.s036.docx]

Replies to Reviewers

**(A) Comments from Reviewer #1**

(1) NCI-H226 is used as a mesothelioma cell line. The website of ATCC shows that this line exhibits epithelial morphology that was isolated from the lungs of a male with squamous cell carcinoma. The website of cBioPortal also shows that the type of this line is lung squamous cell carcinoma. The authors should confirm that what type of cell line NCI-H226 is.

**[Reply]**

Thank you very much for your comment. The ATCC website also showed that this cell line was mesothelioma in addition to lung carcinoma. As mentioned by the reviewer, cBioPortal showed that the cell line was classified as lung carcinoma. The Biohippo and the Cellosaurus websites however mentioned that NCI-H226 was mesothelioma cell line (pleural epithelioid mesothelioma). The confusion or the ambiguity came from several reasons. For example, the cells were derived from pleural effusion of a lung cancer patient and histology of the two cancer types was overlapped. Nevertheless, genetic markers supported that NCI-H226 cells were rather mesothelioma. NCI-H226 cells showed wild-type *TP53* genotype, deletion of the INK4A/ARF region and mutated *NF2* gene (according to Cancer Cell Line Encyclopedia, Catalogue of Somatic Mutations in Cancer, and Cancer Dependency Map). Moreover, NCI-H226 cells strongly positive for mesothelin which was a characteristic protein marker of mesothelium and mesothelioma, but non-small cell lung cancer was negative for mesothelin. While some studies classified the NCI-H226 cell line as lung carcinoma based on sources like ATCC, many others used it as a model for mesothelioma. Given its genetic profile and mesothelin expression, we consider it more appropriate to regard NCI-H226 as a mesothelioma cell line, in line with the majority of previous publications.

(2) The authors concluded that a combination of defactinib and the MDM2 inhibitors in general produced synergistic or additive effects to inhibit growth of mesothelioma. However, as they describe, these effects were different depending on MDM2 inhibitors (nultin-3a or RITA) and cell lines tested. This study lacks impact.

**[Reply]**

Thank you for your comment. We understand the concern that the combinatory effects varied and were to some extent dependent on the type of MDM2 inhibitors and the mesothelioma cell lines used, which may reduce the overall impact of the study. Initially, we considered focusing on nutlin-3a in wild-type p53 cells to simplify our conclusions. However, we expanded the study to include RITA and mutated p53 cells to explore the broader applicability of the combination. While both nutlin-3a and RITA inhibit the MDM2-p53 interaction, RITA also induces DNA damage, which can elevate p53 levels independently. The combinatory effects were therefore influenced by the inhibitors. Our results showed that both MDM2 inhibitor and defactinib produced synergistic or additive inhibitory effects in most of wild-type p53 cells except for NCI-H226 cells treated at a low RITA concentration. In mutated p53 cells treated with nutlin-3a, the combinatory effects were antagonistic. In the case of RITA-treated cells, the combination produced synergistic effects in irrespective of the *TP53* genotype except NCI-226 cells as mention above and H2452 cells with truncated p53.

Furthermore, the effects of MDM2 inhibitors on mutated p53 is influenced by the p53 structure and the MDM2-mediated effects could be altered in mutated p53 cells. Even if the MDM2 inhibitors increases expression levels of mutated p53, the up-regulation would rather support for cell survival but not growth inhibition. The combinatory effects in mutated p53 cells could be therefore different from those in wild-type p53 cells. Nevertheless, the combination of RITA and defactinib achieved synergistic effects in mutated p53 cells. On the other hand, NCI-H2452 cells with truncated p53 and EHMES-10 cells with resistance to nutrin-3a and RITA showed different responses from wild-type p53 cells. The extended experiments thus revealed that the combinatory effects were influenced by MDM2 inhibitors and cells used although the combination produced synergistic or additive effects in most of the cases. We have revised the manuscript to clarify our conclusions and better reflect the scope and limitations of the study. Please see the revised part on page 4-5 line 67-71, on page 8 line 110-111 in unmarked manuscript, and Discussion in marked manuscript.

(3) The part of “Discussion” is redundant.

**[Reply]**

Thank you very much for your comment. We have revised the Discussion section to enhance clarity and eliminate redundancy. Please refer to the revised parts on page 22-27 in marked manuscript.

**(B) Comments from Reviewer #2**

(1) The FAK inhibitor defactinib and the MDM2 inhibitor nutlin-3a were at the micromolar scale, however, the small molecular inhibitor drugs are usually at 10-20 nanomole scale. How about the future drug development?

**[Reply]**

Thank you very much for your insightful point. As the reviewer pointed out, small-molecule inhibitor drugs used in clinical settings typically exhibit efficacy at nanomolar concentrations, whereas the compounds used in our study were applied at micromolar levels. The present study is preclinical and intended to demonstrate proof-of-concept to explore a strategy for up-regulating endogenous wild-type p53 levels. While some molecular-targeted agents are effective at micromolar concentrations, such doses may increase off-target risks in clinical applications. Future drug development should therefore aim for high target affinity binding, extended half-life and enhanced tissue distribution to improve clinical feasibilities. We added a statement regarding this possible future direction in Discussion of the revised manuscript (See on page 27 line 433-436 with references, No 38 and 39 in unmarked manuscript).

(2) The combination effects were mutation and cell line specific. How to combine the findings of this study with the subgroup mesothelioma patients?

**[Reply]**

Thank you very much for your valuable comment. The reviewer referred to the histological subtypes of mesothelioma-epithelioid, sarcomatoid, and biphasic-as well as their associated genetic alterations. In general, the epithelioid subtype tends to retain wild-type *TP53* genotype, whereas sarcomatoid and biphasic types are more likely to harbor *TP53* mutations; consequently, the epithelioid subtype is more susceptible to combination therapy aimed at up-regulating wild-type p53. On the other hand, RITA increases susceptibility to the combination even in cells with mutated p53. Based on our findings, we suggest that the combination of nutlin-3a and defactinib is effective for the epithelioid subtype, while the combination of RITA and defactinib is more suitable for sarcomatoid and biphasic types. We discussed these possible relationships between histological subtypes and combination effects in the revised manuscript (page 26-27 line 422-427 in unmarked manuscript). In addition, sensitivity to defactinib was not linked to *TP53* mutation status (page 27 line 427 in unmarked manuscript).

(3) The immune check point inhibitors have made great success in the treatment of mesothelioma patients. How about the influence of both FAK and MDM2 inhibitors to the expression level of PD-L1 in mesothelioma cells?

**[Reply]**

Thank you very much for highlighting the importance of immune checkpoint inhibitors in mesothelioma treatment. We fully agree with the reviewer’s point. Although PD-L1 expression has been widely studied, its predictive value in mesothelioma patients receiving immune checkpoint inhibitors remains inconclusive at this moment as far as we are aware. Nevertheless, FAK inhibition may reduce PD-L1 expression and p53 is known to down-regulates PD-L1 expression. We therefore believe that a potential combination of FAK inhibitors, MDM2 inhibitors and immune check point inhibitors represents an interesting research direction. We however presume that investigating PD-L1 expression following treatment with FAK and MDM2 inhibitors will be difficult to include in this study since it may fall outside the scope of the present study. Such analyses may be the next investigation because it requires separate, dedicated studies. We hope that the future studies, possibly including in vivo models or patient-derived samples, will clarify the impact of these inhibitors on PD-L1 expression and their potential synergy with immune checkpoint therapies. We appreciate the reviewer’s suggestion and have included this point in the revised Discussion section on page 27 line 428-433 with references in unmarked manuscript.

(4) The antiangiogenic therapy has been proved valuable in relieving pleural effusion. How about the influence of both FAK and MDM2 inhibitors to the expression level of VEGF in mesothelioma cells?

**[Reply]**

Thank you very much for your comment. As the reviewer mentioned, recent studies indicated that inhibition of angiogenesis was effective, particularly when combined with chemotherapy and immunotherapy. Nevertheless, the clinical value of VEGF expression as a predictive marker remains unclear at this time. On the other hand, FAK is involved in VEGF regulation and angiogenesis, and p53 up-regulation can negatively regulate VEGF transcription. These findings suggest that FAK and MDM2 inhibitors can potentially enhance efficacy of the antiangiogenesis therapy. We however believe that analyzing the VEGF expression following the treatments is important but may fall beyond the scope of the current work at this moment. We consider this a promising direction for future research and have mentioned the potential combination with antiangiogenesis therapy in revised Discussion section (page 27 line 428-433 and references in unmarked manuscript).

(5) There is no proper statistics in the figures and figure legends. All the WB results should have statistics of gray values.

**[Reply]**

We appreciate the reviewer’s comment regarding statistical analysis of the Western blot data. In our study, we performed densitometric quantification using ImageJ and normalized each band to the corresponding loading control. We think at this moment that it is unfortunately not feasible to repeat all the analyses from the original materials due to the large number of samples. We conducted multiple Western blot experiments to obtain clear and consistent data, and selected representative results for presentation in the figures. We therefore are confident in the accuracy and reproducibility of the data. While we acknowledge that statistical testing across biological replicates is ideal, the scope and limitations of our dataset make this approach challenging. We have clarified this point in the revised manuscript and added a note in the figure legend (Figure 1, 2B, 3B, 4B, 7B) to explain the methodology. We hope this explanation addresses the reviewer’s concern and respectfully request understanding given the constraints of our study.
