# Supplementary material for: Strategic selection of MDM2 inhibitors enhances the efficacy of FAK inhibition in mesothelioma based on TP53 genotype
Source: PLoS One. 2026 Feb 23;21(2):e0343551. doi: 10.1371/journal.pone.0343551 (PMC12928570; doi:10.1371/journal.pone.0343551)
Supplement: Attachment — Submitted filename: Comments for reviewers 2026-2-4.docx [file pone.0343551.s037.docx]

Replies to Reviewer #3

**Major concerns**

Immunonblotting is not easy methodology, and I think authors did a good job, showing all blots, full scan in supplement. But there are major issues with MDM2, gH2AX, dirty antibodies and comparison issues. Control for antibodies. Also, authors state they conducted multiple immunoblot experiments but show only representative results. Response to reviewer 2 is to some extent inadequate "it is unfortunately not feasible to repeat all the analyses". They claim confidence in accuracy and reproducibility without providing statistics. This becomes more of an issue given concerns below. In general a better approach is statistical analysis of western blots from multiple independent experiments, time and dose response. Add blot statistics from ≥3 independent experiments. Quantify blots with error bars and statistical tests where effect is limited or vague/unclear. Clarify blotting methodology approach and add time course and dose experiments (not just 24 hrs). I understand the issue of repeating many experiments and that some are repeated within the paper from figure to figure. However, regardless of this there are major issues remaining.

**[Reply]**

We appreciate the reviewer’s constructive comments regarding our immunoblotting methodology. We have addressed the specific concerns regarding MDM2 and γH2AX (p-H2AX) in the subsequent itemized responses as below. Here, we clarify the rationale for our choice of treatment duration and dosage.

For defactinib, a concentration of 8 µM resulted in excessive cell death, which interfered with the detection of clear FAK bands. Furthermore, while the treatment beyond 24 hrs effectively induced FAK dephosphorylation, prolonged exposure led to significant cytotoxicity. Consequently, we selected a concentration range of 1–4 µM and a 24-hrs incubation period to demonstrate FAK inhibition without compromising cell viability.

Regarding nutlin-3a and RITA, the concentrations used in Figures 3B (nutlin-3a; 0, 5 and 10 μM) and 4B (RITA; 0, 1.5, 3 μM) were optimized to ensure p53 induction without significant toxicity. Since p53 expression levels remained consistent for 24 hrs after the defactinib treatment and across 24, 48, and 72 hrs of the nutlin-3a/RITA treatment, we standardized the incubation period to 24 hrs. This duration was also applied to the combination treatments to maximize p53 stability while minimizing defactinib-induced cell death, thereby ensuring the reliability of the immunoblotting results. We therefore conducted preliminary experiments and selected the optimal condition regarding treatment time and dose.

**Comment (1)** 1 There is a major issue with MDM2 (figure 2), as it is shown in main figure, it looks like MDM2 and p-MDM2 bands are similar in size and overlap, but they are not looking at the full scan the bands don’t fully match. It is not possible to deduce what we are looking at or that the MDM2 bands are what they are. Also no size markers for the main figure. In supplement is clear non-modified MDM2 bands do not correspond to sizes of p-MDM2. And, it is not clear that this is actually MDM2 to begin with. What is the evidence that p90, p60 bands as shown is a actually MDM2?

**[Reply]**

Thank you for pointing out the inconsistencies regarding the MDM2 and p-MDM2 blots. We realized our mistakes of p-MDM blots in Figure 2B and Supplementary Figure 10 (the corresponding original full blot) and noticed labeling errors in both of the figures.

Specifically, the bands previously labeled as 90 kDa and 60 kDa in Supplementary Figure 10 were incorrect. Upon re-examination of molecular weight markers and additional sample blots (see below), the band previously indicated as 90 kDa was a non-specific signal appearing above 100 kDa, while the band previously labeled as 60 kDa is the authentic p-MDM2 at 90 kDa. Authentic 60 kDa p-MDM2 bands were quite low in the intensity with almost background levels because 60 kDa MDM-2 molecules lost the phosphorylation site at Ser 166 and thereby the anti-p-MDM2 antibody used in the study could not detect 60 kDa p-MDM2. We then removed the 60 kDa p-MDM2 arrows in the revised Figure 2B and Supplementary Figure 10.

We have up-loaded new Figure 1, 2, Supplementary Figure 10 and Supplementary Table 4 to address your concerns.

**(a)** new Figure 2B & Supplementary Figure 10: We have replaced the p-MDM2 blots with the correct 90 kDa bands and added molecular weight markers as requested. We have also revised Supplementary Figure 10 legend (line 737 in page 45). The identification of the 90 kDa band as MDM2/p-MDM2 is based on the manufacturer's datasheet (Santa Cruz Biotechnology and Cell Signaling) and its alignment with the protein standards.

**(b)** new Supplementary Table 4: We have updated the data. We removed the non-specific (previously "90 kDa") data and provided the intensity data for the authentic 90 kDa p-MDM2 (previously "60 kDa"). Despite these labeling corrections, the trend of p-MDM2 levels in response to defactinib treatment remained consistent with our original findings; the intensity data of non-specific (previously “90 kDa”) bands were similar to those of authentic 90 kDa p-MDM2 (previously "60 kDa"). The overall conclusion of the study thereby remains unchanged.

**(c)** Size markers*:* We added the size markers in MDM2 and p-MDM2 blots of revised Figure 2B and also in Figure 1 to ensure clarity.

We apologize for the confusion caused by the initial mislabeling.

**Comment (2)** 2 Second major issue concerns comparability of basal gH2AX (figure 3 versus figure 4, respective cell line untreated condition). The level of gH2AX in untreated cells (respective cells) varies a lot between experiments for the same cell line. Presumably this could be to different exposure times,between lines/experiments though this does not seem to be an issue for p53. This makes it very difficult to judge and compare gH2AX. Besides, the antibody is dirty, many bands.

**[Reply A]**

Thank you for your comment. The reviewer is correct that the level of p-H2AX in untreated cells appeared different between Figure 3B and 4B even for the same cell line. This discrepancy is due to different exposure times, which were optimized to capture the specific effects of each inhibitor. We needed to show whether nutlin-3a or RITA induced DNA damage, and consequently we had to use a different exposure time depending on the property of the inhibitor.

**(a)** Figure 3B (nutlin-3a): All the cells showed relatively high expression levels even in untreated cells. Since nutlin-3a did not induce DNA damage, the p-H2AX signals were inherently weak across all the samples. We intentionally exposed the filters for a long period until the basic signals in untreated cells became clearly detectable in order to confirm whether the western blotting was technically successful and to demonstrate the lack of p-H2AX induction, A short exposure showed no p-H2AX signal in all the lanes, which made it difficult to judge whether the western blots were properly conducted.

**(b)** Figure 4B (RITA): In contrast, RITA is a potent inducer of DNA damage. We used a shorter exposure time to prevent the strong signals in RITA-treated cells from reaching saturation. Consequently, the basal levels in untreated cells (with the exception of MSTO-211H) appeared undetectable under these conditions. MSTO-211H cells showed less sensitivity to RITA-induced DNA damage compared with other cells. To visualize their response, we applied a longer exposure specifically to this cell line. We therefore added sentences in Figure 3B and 4B legends as follows to explain more in detail. We also revised Results section to explain results of RITA-treated MSTO-211H cells.

**(a)** Figure 3 legend (lines 640-642 in page 40)

For phosphorylated H2AX, the blots were exposed for a longer period to ensure the detection of basal signals in untreated cells. The basal intensity therefore differed from that of the RITA experiments in Figure 4B where shorter exposures were used.

**(b)** Figure 4 Legend (lines 651-652 in page 40)

For phosphorylated H2AX of MSTO-211H cells, the blot was exposed for a longer period to ensure the detection of basal signals in untreated cells.

**(c)** Results **(**line 274-275 in page 17)

(Before the revision)

All the RITA-treated cells showed decreased FAK phosphorylation levels and increased phosphorylation of H2AX.

(After the revision)

All the RITA-treated cells showed decreased FAK phosphorylation and increased H2AX phosphorylation levels, with the exception of MSTO-211H cells exhibiting a minor H2AX increase.

**[Reply B]**

Regarding the "dirty" appearance and multiple bands mentioned by the reviewer, this was also a result of the extended exposure times required for the nutlin-3a experiments (as seen in Supplementary Figure 16, which is for Figure 3B p-H2AX). The high background and non-specific bands emerged only under prolonged exposure to visualize the weak basal signals. For nutlin-3a treatment, we needed to expose the filters for a long period to obtain the signal in untreated cells since nutlin-3a did not induce DNA damage. In contrast, the blots in Supplementary Figure 20 (RITA treatment, used for Figure 4B p-H2AX) showed a cleaner background due to the shorter exposure time which enabled us to detect the DNA damage signal. We used the same antibody lot from BioLegend for all experiments, and we would like to maintain that the extra bands were an artifact of overexposure rather than a lack of antibody specificity.

**Comment (3)** 3.Third major issue, how come there is such increase in p-p53 upon Nutlin treatment same almost as with RITA? It looks quite a lot, though corresponding to total levels. Figure 3 vs 4. This nutlin effect on p53-p is not really that apparent in figure 7 e g in NCI H28, this really contracts within the paper. Similar to gH2AX.

**[Reply]**

Thank you for this critical observation regarding the p-p53 levels. We understand the reviewer's concern about the apparent discrepancy between Figure 3B and Figure 7. The reviewer mentioned that p-p53 in the wild-type *TP53* cells treated with nutlin-3a significantly increased as much as that in the cells treated with RITA, and questioned that the nutlin-3a-mediated up-regulation of p-p53 was not significant in Figure 7. The difference in p-p53 induction levels between these figures is primarily due to the different concentrations of nutlin-3a used in each experiment, as detailed below.

**(a)** Concentrations: In Figure 3B, we aimed to demonstrate the maximum biological effect of nutlin-3a as a single agent. In contrast, for the combination studies in Figure 7, we intentionally used nutlin-3a at concentrations lower than the IC_50_ values in growth inhibitory study (4 μM of nutlin-3a for NCI-H28, 1 μM for MSTO-211H and 2 μM for NCI-H226). All the nutlin-3a concentrations used in Figure 7 were thus lower than those in Figure 3B. Please see the Figure 7 legend in line 671-674 page 41-42, which mentioned the concentrations in detail. We also explained the reason for different concentrations used in Figure 7 in the legend in page 669-671 in page 41. In that sense, the situation is the same as p-H2AX in Figure 3B and 4B, which used different exposure times.

**(b)** Rationale for Figure 7: We selected these lower doses to avoid a "ceiling effect," where a high dose of nutlin-3a alone might saturate the p53 response, thereby masking any synergistic or additive effects when combined with defactinib. Consequently, the up-regulation of p-p53 in Figure 7 appears less pronounced than in Figure 3B.

**(c)** Mechanism of up-regulation: While nutlin-3a increases p53 levels by inhibiting MDM2-mediated degradation, RITA can further enhance p53 phosphorylation through the induction of DNA damage and subsequent kinase activation. Furthermore, many factors influence the augmentation levels of p53 in cells treated with MDM2 inhibitors such as stability and affinity of the inhibitors, and susceptibility to ubiquitination in respective cells. The expression levels of p-p53 are therefore the outcomes of these factors involved. As shown in Supplementary Tables 5 (nutlin-3a) and 6 (RITA), the phosphorylated p53 levels varies depending on the cells and the inhibitor used. Phosphorylated p53 levels mediated by nutlin-3a were not always similar to those by RITA.

**Comment (4)** 4 Drugs and cell lines. IC50 values in 1-6 micromolar range is not so good actually, effects vary dramatically between cell lines (though expected). EHMES-10 resistant to both MDM2 inhibitors it looks like and NCI-H226 shows unusual responses in my opinion. Variability limits generalizability and clinical translation.

**[Reply]**

Thank you very much for your thoughtful comment regarding the drug sensitivities and clinical translation. We agree that the observed variability and the micromolar-range IC_50_ values are critical points.

**(a)** Variability and *TP53* genotype: A key finding of this study is that sensitivity of nutlin-3a was dependent on the *TP53* genotype. We showed that wild-type *TP53* cells were sensitive, whereas mutated or non-functional p53 cells (NCI-H2452, Met-5A cells) were resistant (line 234-236 in page 15). This suggests that the INK4A/ARF deletion with wild-type *TP53*, often found in the majority of mesothelioma, could serve as a biomarker for MDM2 inhibitor sensitivity. We presume that identifying such "resistant" and "sensitive" profiles is not a limitation but rather a step toward patient stratification and personalized medicine. In contrast, sensitivity to defectinib was not linked with the *TP53* genotype and sensitivity range of defectinib (Figure 2A) was not as great as that of nutlin-3a (in Figure 3A). Furthermore, sensitivity to RITA was not linked with the *TP53* genotype but can be influenced by induction of DNA damage responses. Drug sensitivity is influenced by many factors such as genetic alterations and modifications of the target proteins; consequently, the sensitivity will be variable depending on properties of inhibitors and target cells. This study indicated that the sensitivity was subjected the agents and cells tested although the *TP53* genotype can play a role in the sensitivity.

**(b)** EHMES-10 NCI-H226 cells: As the reviewer noted, EHMES-10 cells exhibited resistance to both inhibitors despite being wild-type *TP53*. We mentioned this in the Results section and suggested that a defect in downstream p53 pathways (e.g., impaired p21 induction) may result in reduced cell cycle arrest and contribute to this phenotype. We modified the sentence to explain more (lines 240–242 in page 15). Regarding NCI-H226, its response to nutlin-3a was relatively consistent with other wild-type *TP53* cells; however, we acknowledge that differences in the RITA sensitivity probably due to some kinds of protein modification levels or other compensatory pathways, both of which might influence DNA damage responses and inhibition of MDM2 activity.

**(c)** Clinical translation: We acknowledge that the potency of the inhibitors used in this study will be lower than that of current small-molecule inhibitors that operate in the nanomolar range. We agree that high-dose applications increase the risk of off-target effects. This study however serves as a preclinical proof-of-concept to explore the synergy between p53 stabilization and FAK inhibition. These findings provide a rationale for developing next-generation agents with higher affinity and better pharmacological profiles. We revised the Discussion section and explained more (line 417-421 and line 422-423 in page 26). We also added reference 40 for idasanutlin which was mentioned by the reviewer.

**(d)** Future drug development: We would like to contend that "negative" results or variability are informative. Our data suggest that while this combination is promising for epithelioid type mesothelioma with wild-type *TP53* genotype, different strategies will be required for sarcomatoid or biphasic types which often have mutated *TP53* genotype (line 407-411 in page 25-26). We believe that variability of drug sensitivity will not be negative in terms of clinical applications but rather stimulate further investigations. We mentioned these in Discussion as above.

**Comment (5)** 5 Direct comparisons difficult since only one exposure duration tested.

**[Reply]**

Thank you very much for your valuable comment. The reviewer mentioned that western blot data were difficult to judge since only one exposed duration.

**(a)** Optimal exposure duration: The single exposure duration presented for each blot was selected after preliminary experiments across several time points and drug doses. A low concentration and short drug exposure time did not show any significant changes, whereas a high concentration and longer drug exposure time resulted in cell death which did not reflect molecular changes. The chosen condition was determined to provide the optimal signal-to-noise ratio, allowing for clear visualization of the specific molecular changes we intended to illustrate (please also see our Reply to Major concerns above). Shorter exposures did not show significant changes, while longer exposures saturated signals or introduced excessive background.

**(b)** Reproducibility: Although we presented a single representative exposure, the experiments were repeated several times (typically 2-3 times as for a single blot from the same sample) to ensure the reliability and reproducibility of the results. We selected the most representative blot for presentation in the figures.

**(c)** Statistical analysis: While we agree that robust statistical analysis often requires more than three independent replicates, the trends observed in this study were consistent across all the experiments. We included the semi-quantitative intensity data in Supplementary Tables 4-7 to provide full transparency and facilitate the reviewers' judgment on the expression level changes. In addition, we are not confident whether PLOS ONE always requires three independent replicates to show statistical analysis even in such multiple western blot experiments.

We added a sentence and clarified in the Materials and Methods section of the revised manuscript that the exposure times were optimized for each specific experiment and the representative blots were shown (line 166-167 in page 11). We believe this approach ensures that our findings were clearly presented.

**Comment (6)** 6 Figure 7: Complex dosing scheme makes interpretation difficult

**[Reply]**

Thank you very much for your comment. We understand that the variety of drug concentrations used in the combination experiments in Figure 7 may appear complex. As explained in the manuscript, the concentrations of defactinib and MDM2 inhibitors were carefully optimized for each cell line to evaluate their synergistic or additive effects effectively. We revised a sentence to explained well (line 301-304 in page 19).

**(a)** Avoid ceiling effects: We selected concentrations lower than the IC_50_ values of each single agent. A high concentration of either drug alone could induce strong growth inhibition and significant molecular changes, masking the additional impact of the combination treatment.

**(b)** Cell type optimization: Since each mesothelioma cell line exhibits a different sensitivity profile (Figures 2A, 3A and 4A), uniform concentration across all lines would not allow for a fair assessment of the combination's efficacy. We listed the exact concentrations used for each cell line to ensure transparency in Figure 7 legend (line 671-674 in page 41-42) and added a sentence to explain the optimization. “We selected concentrations lower than the IC_50_ values of each single agent to detect the molecular changes induced by the combination.” (line 669-671 in page 41).

**Comment (7)** 7 Discussion is still somewhat redundant despite revisions.

**[Reply]**

Thank you very much for your comment. We revised the Discussion part. Please look at the highlighted version and the revised clean/unmarked version.

**Comment (8)** 8 Abstract conclusion is very vague just says "effects depend on genotype and inhibitor type"

**[Reply]**

Thank you very much for your comment. We revised the part as follows.

(Before the revision)

These results suggested that FAK and MDM2 inhibition suppressed cell growth of mesothelioma, but the combinatory effects depended on *TP53* genotype and the type of MDM2 inhibitors used.

(After the revision)

These results suggest that the therapeutic success of combined FAK and MDM2 inhibition in mesothelioma depends on the precise matching of MDM2 inhibitors with the *TP53* genotypes, and highlight the need for genotype-based selection of MDM2 inhibitors.

**Comment** **(9)** 9 The title is not really what the data shows, it is a bit confusing.

**[Reply]**

Thank you very much for your comment. We agree that the original title was a bit confusing and did not accurately reflect outcomes of our study. We changed the title as follows.

“Strategic selection of MDM2 inhibitors enhances the efficacy of FAK inhibition in mesothelioma based on *TP53* genotype”
