# Supplementary material for: Correction: Strategic selection of MDM2 inhibitors enhances the efficacy of FAK inhibition in mesothelioma based on TP53 genotype
Source: PLoS One. 2026 Oct 26;21(10):e0361362. doi: 10.1371/journal.pone.0361362 (PMC13704855; doi:10.1371/journal.pone.0361362)
Supplement: Supplementary file 2 [file pone.0361362.s005.pdf]

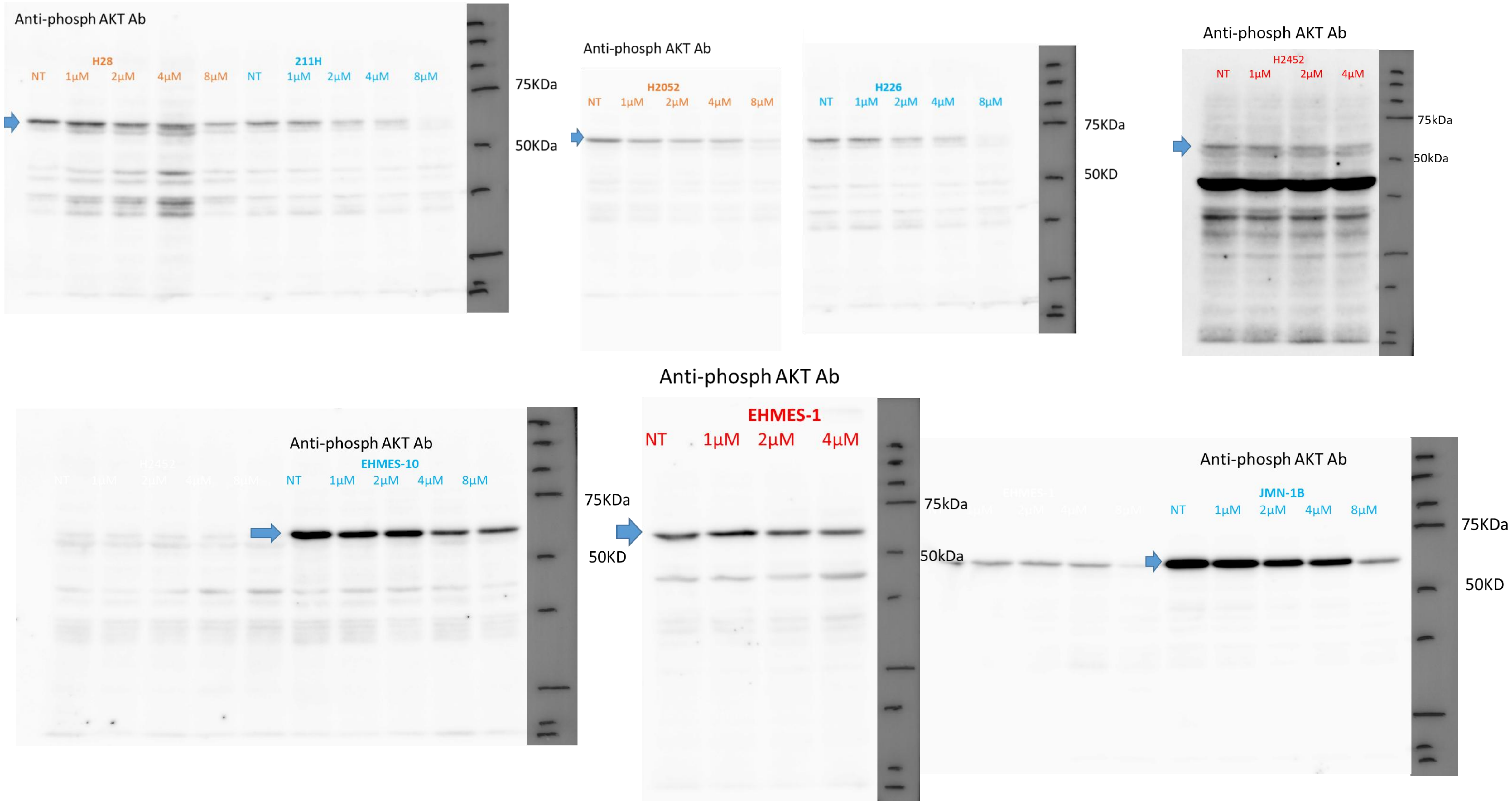
Anti-phosph AKT Ab
H28
NT 1μM 2μM 4μM 8μM
211H
NT 1μM 2μM 4μM 8μM
75KDa
50KDa
Anti-phosph AKT Ab
H2052
NT 1μM 2μM 4μM 8μM
H226
NT 1μM 2μM 4μM 8μM
75KDa
50KD
Anti-phosph AKT Ab
H2452
NT 1μM 2μM 4μM
75kDa
50kDa
Anti-phosph AKT Ab
EHMES-10
NT 1μM 2μM 4μM 8μM
75KDa
50KD
Anti-phosph AKT Ab
EHMES-1
NT 1μM 2μM 4μM
75KDa
50kDa
Anti-phosph AKT Ab
JMN-1B
NT 1μM 2μM 4μM 8μM
75KDa
50KD
