# Supplementary material for: Correction: Strategic selection of MDM2 inhibitors enhances the efficacy of FAK inhibition in mesothelioma based on TP53 genotype
Source: PLoS One. 2026 Oct 26;21(10):e0361362. doi: 10.1371/journal.pone.0361362 (PMC13704855; doi:10.1371/journal.pone.0361362)
Supplement: Supplementary file 3 [file pone.0361362.s009.pdf]

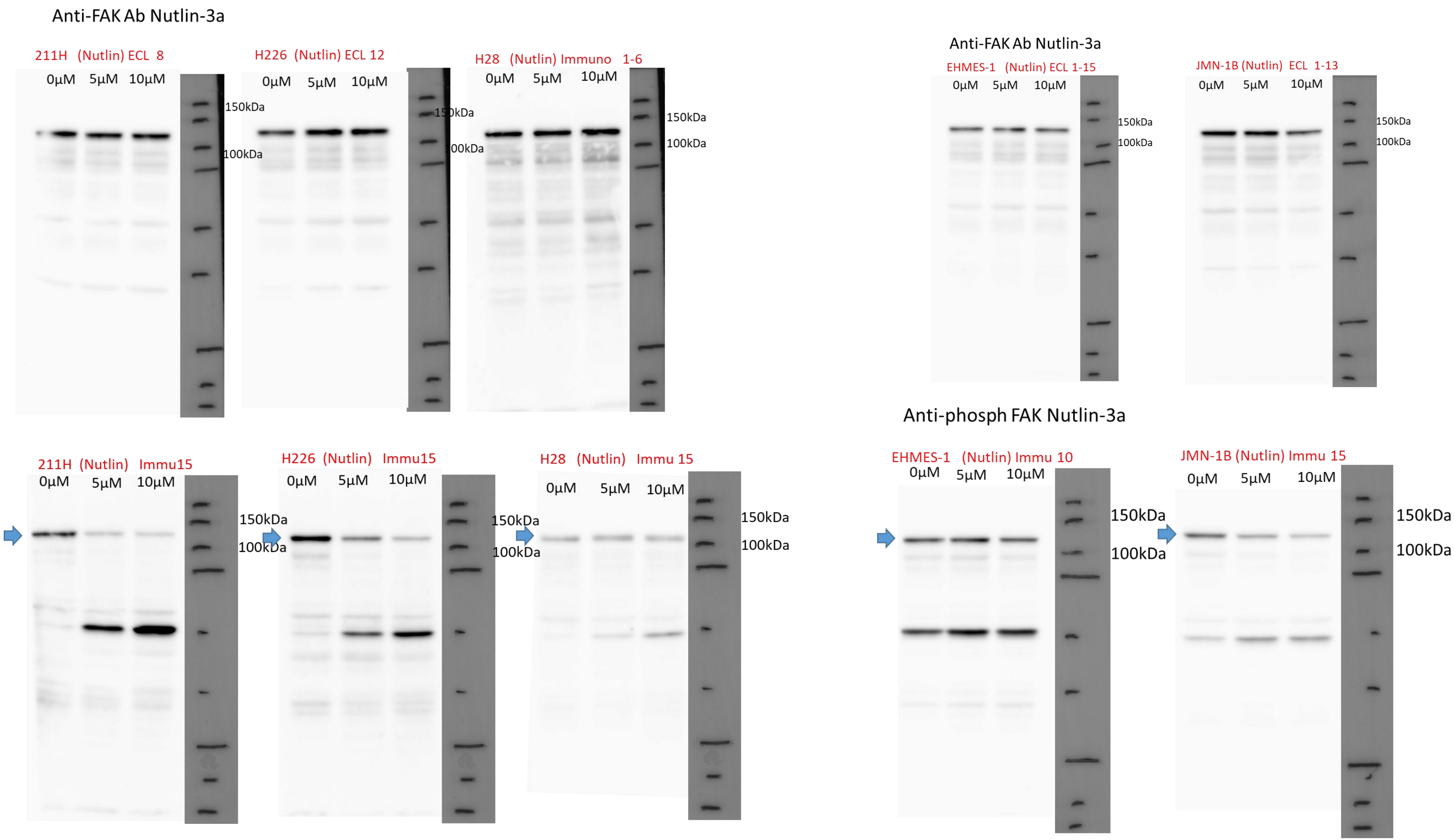
Anti-FAK Ab Nutlin-3a
211H (Nutlin) ECL 8
0µM 5µM 10µM
150kDa
100kDa
H226 (Nutlin) ECL 12
0µM 5µM 10µM
150kDa
100kDa
H28 (Nutlin) Immuno 1-6
0µM 5µM 10µM
150kDa
100kDa
Anti-FAK Ab Nutlin-3a
EHMES-1 (Nutlin) ECL 1-15
0µM 5µM 10µM
150kDa
100kDa
JMN-1B (Nutlin) ECL 1-13
0µM 5µM 10µM
150kDa
100kDa
211H (Nutlin) Immu15
0µM 5µM 10µM
150kDa
100kDa
H226 (Nutlin) Immu15
0µM 5µM 10µM
150kDa
100kDa
H28 (Nutlin) Immu 15
0µM 5µM 10µM
150kDa
100kDa
Anti-phosph FAK Nutlin-3a
EHMES-1 (Nutlin) Immu 10
0µM 5µM 10µM
150kDa
100kDa
JMN-1B (Nutlin) Immu 15
0µM 5µM 10µM
150kDa
100kDa
