# Supplementary material for: Correction: Strategic selection of MDM2 inhibitors enhances the efficacy of FAK inhibition in mesothelioma based on TP53 genotype
Source: PLoS One. 2026 Oct 26;21(10):e0361362. doi: 10.1371/journal.pone.0361362 (PMC13704855; doi:10.1371/journal.pone.0361362)

2018.06.08 Exp64 WB Phospho-FAK (Tyr397) Cell Signaling
125kDa 9%Gel ImmunoStarで検出 Membrane ２-2 Nutlin-3a

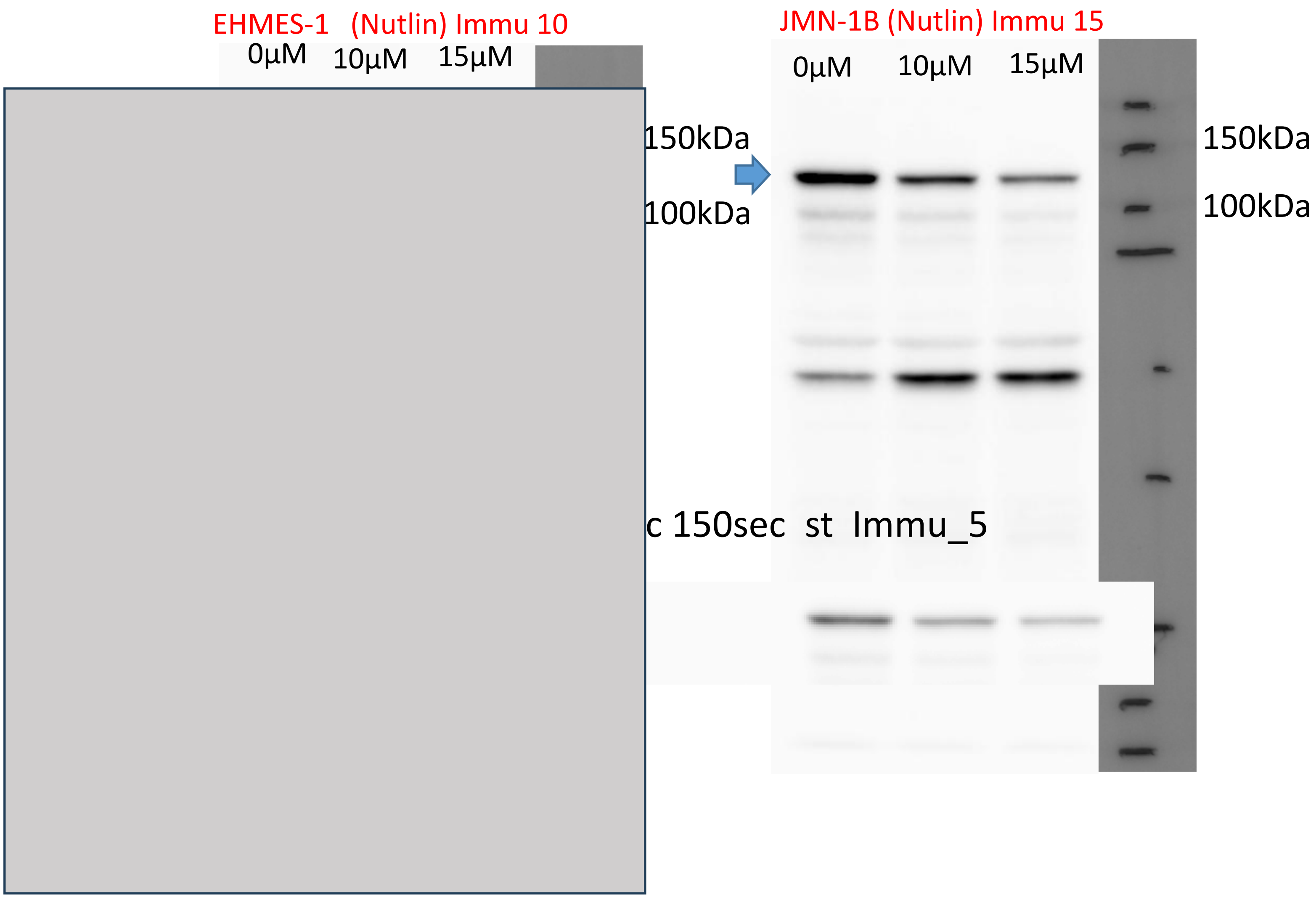

2018.06.08 Exp64 WB Phospho-FAK (Tyr397) Cell Signaling
125kDa 9%Gel ImmunoStarで検出 Membrane 4-2 RITA

EHMES-1 (RITA) Immu 1-8

0μM 1.5μM 3μM

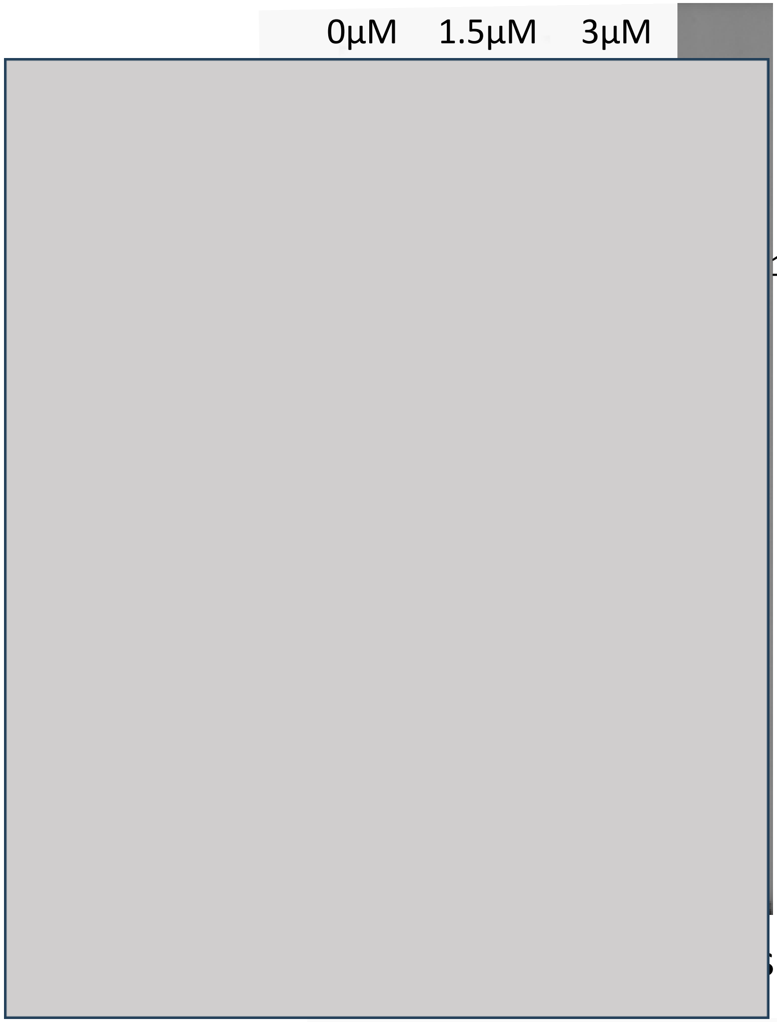

150kDa
100kDa

JMN-1B (RITA) Immu 1-8

0μM 1.5μM 3μM

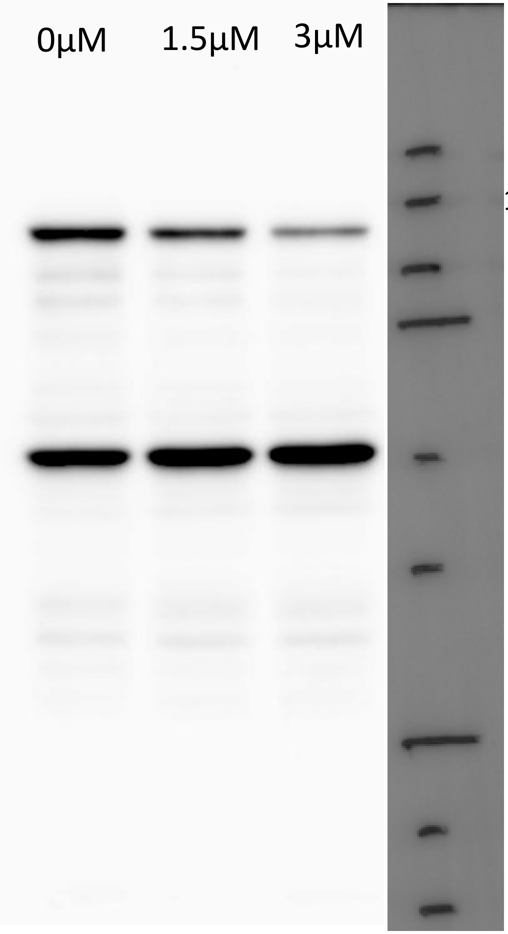

150kDa
100kDa

[illegible]ec 450sec st Immu _1_5

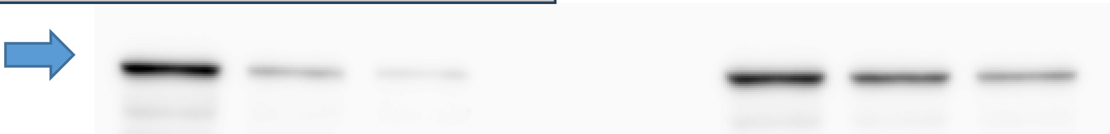

Supplement: Supplementary file 4 [file pone.0361362.s001.zip › 5 Query 1 No3 JMN-Nutlin+RITA.pdf]
