# Supplementary material for: Correction: Strategic selection of MDM2 inhibitors enhances the efficacy of FAK inhibition in mesothelioma based on TP53 genotype
Source: PLoS One. 2026 Oct 26;21(10):e0361362. doi: 10.1371/journal.pone.0361362 (PMC13704855; doi:10.1371/journal.pone.0361362)

2018.04.24 Exp54 追加実験 WB
β- Actin (13E5) Cell Signaling 45kDa 10%Gell ECLで
検出 Membrane 9 RITA

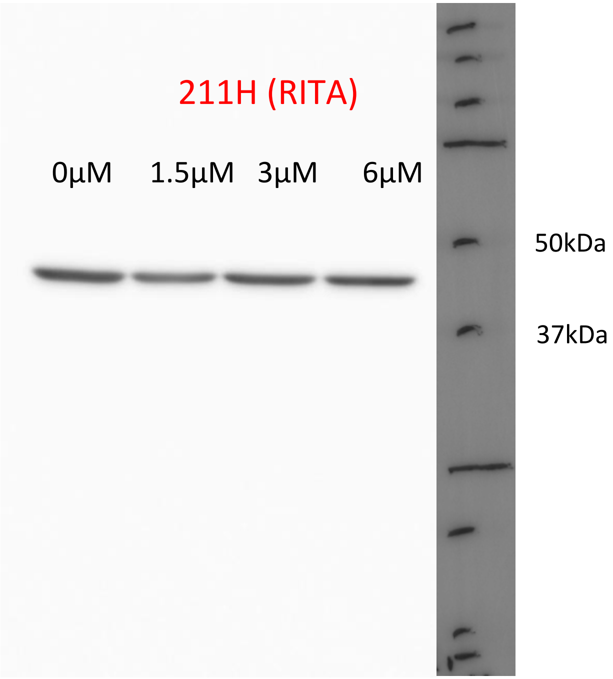

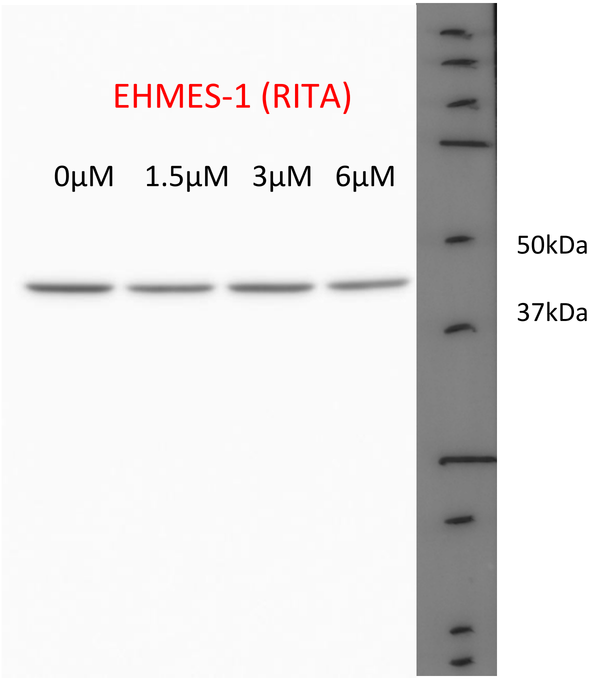

Supplement: Supplementary file 5 [file pone.0361362.s002.zip › 7 Query 1 No4-2 211H+EH-1 summary.pdf]
