# Supplementary material for: Correction: Strategic selection of MDM2 inhibitors enhances the efficacy of FAK inhibition in mesothelioma based on TP53 genotype
Source: PLoS One. 2026 Oct 26;21(10):e0361362. doi: 10.1371/journal.pone.0361362 (PMC13704855; doi:10.1371/journal.pone.0361362)

2017.12.05
Exp59　WB Defactinibの効果　　FAK / P-FAK 発現
FAK (Tyr397)　Cell Signaling　125KDa　10%Gel　ECLで
検出　Membrane　20-2　(Membrane 20 (H2452, EHMES-10)
をそのまま使用)

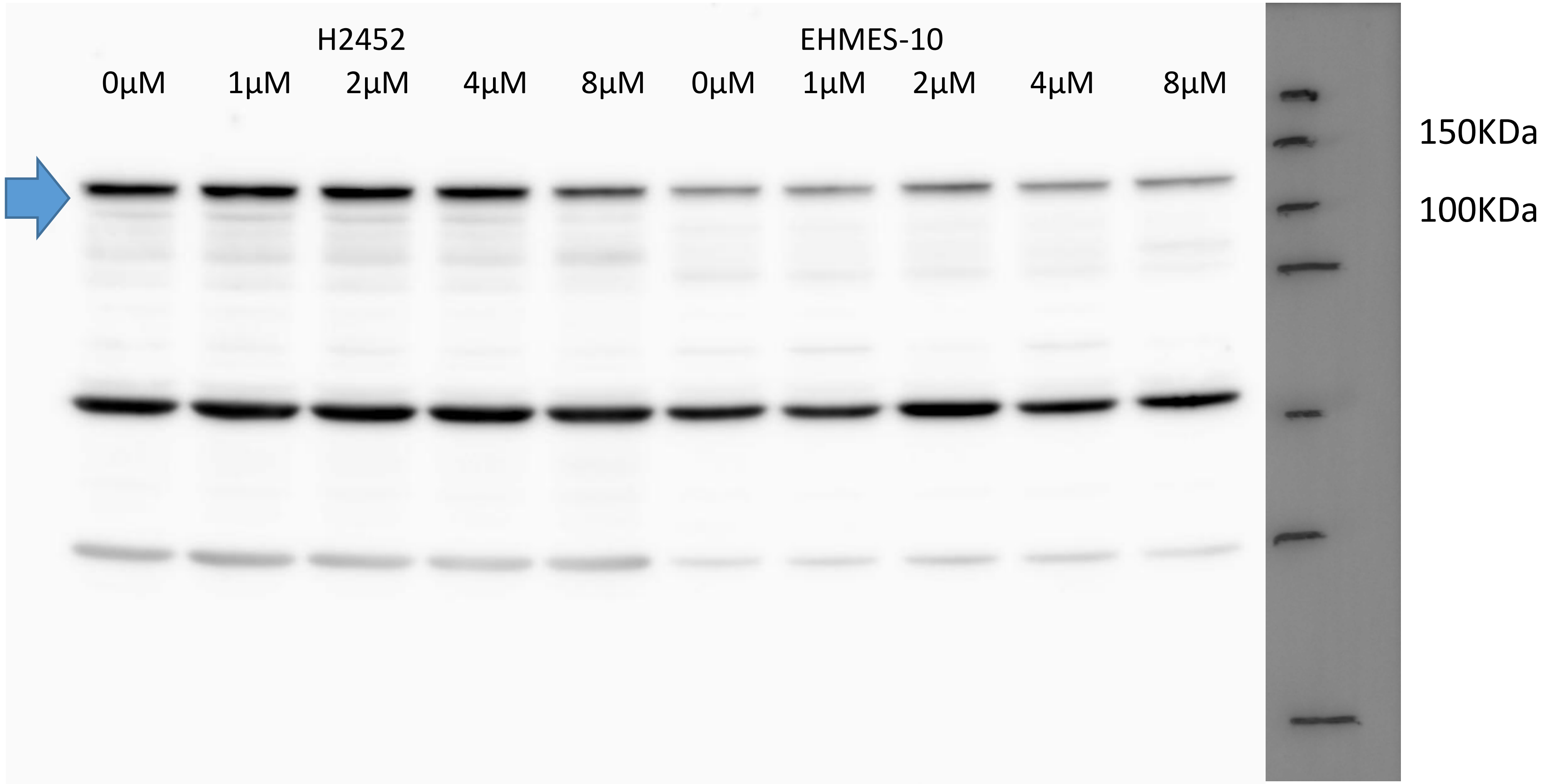

Supplement: Supplementary file 6 [file pone.0361362.s003.zip › 9 Query 2 No1+2 H2452+EH10 right.pdf]
