# Supplementary material for: Correction: Strategic selection of MDM2 inhibitors enhances the efficacy of FAK inhibition in mesothelioma based on TP53 genotype
Source: PLoS One. 2026 Oct 26;21(10):e0361362. doi: 10.1371/journal.pone.0361362 (PMC13704855; doi:10.1371/journal.pone.0361362)
Supplement: Supplementary file 6 [file pone.0361362.s003.zip › 10 Supple Fig 3 (Figure 2B FAK) Revised.pdf]

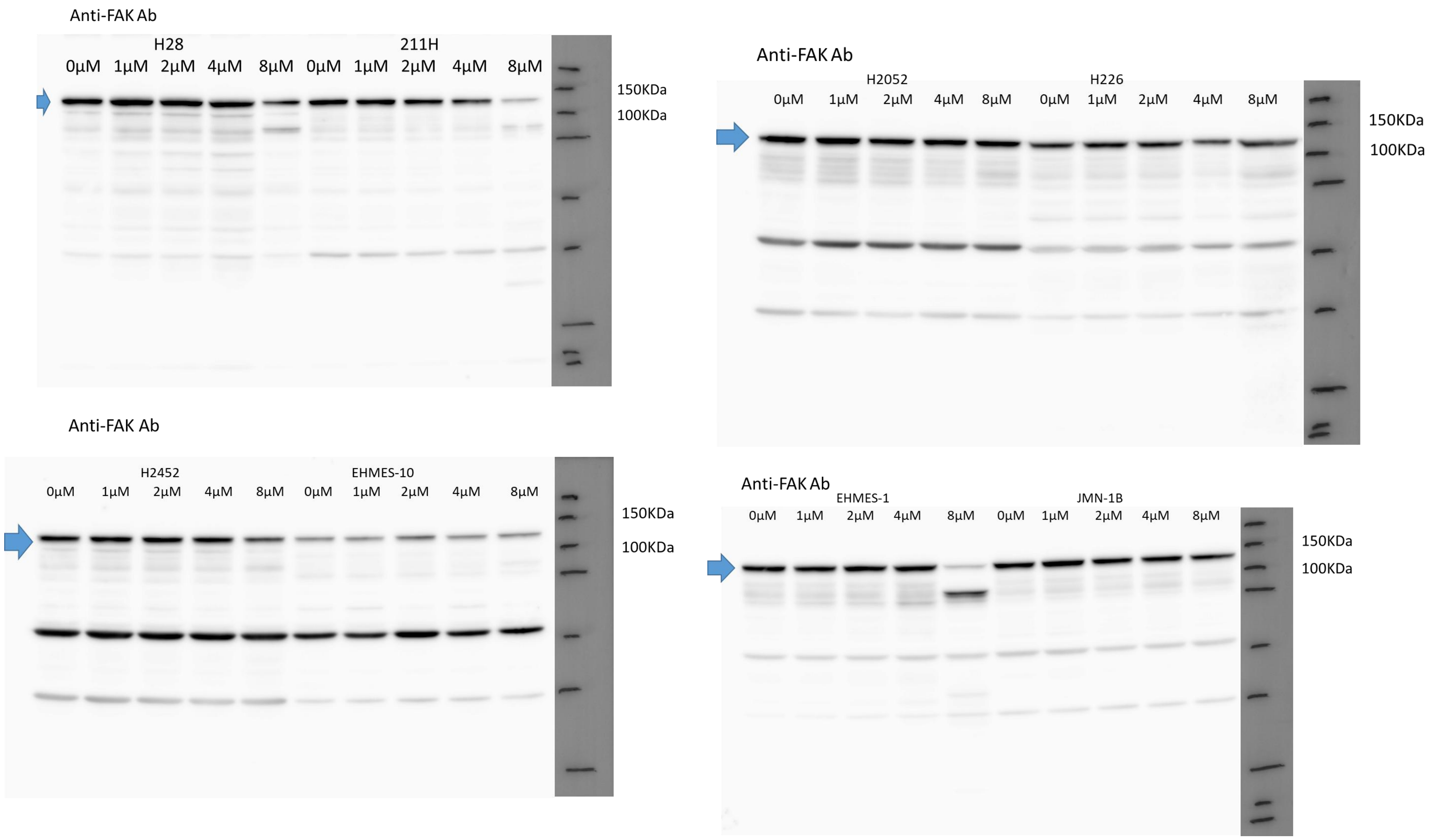
Anti-FAK Ab
H28
211H
0μM 1μM 2μM 4μM 8μM 0μM 1μM 2μM 4μM 8μM
150KDa
100KDa
Anti-FAK Ab
H2052
H226
0μM 1μM 2μM 4μM 8μM 0μM 1μM 2μM 4μM 8μM
150KDa
100KDa
Anti-FAK Ab
H2452
EHMES-10
0μM 1μM 2μM 4μM 8μM 0μM 1μM 2μM 4μM 8μM
150KDa
100KDa
Anti-FAK Ab
EHMES-1
JMN-1B
0μM 1μM 2μM 4μM 8μM 0μM 1μM 2μM 4μM 8μM
150KDa
100KDa
