# Supplementary material for: Correction: Strategic selection of MDM2 inhibitors enhances the efficacy of FAK inhibition in mesothelioma based on TP53 genotype
Source: PLoS One. 2026 Oct 26;21(10):e0361362. doi: 10.1371/journal.pone.0361362 (PMC13704855; doi:10.1371/journal.pone.0361362)

Anti-p53 Ab

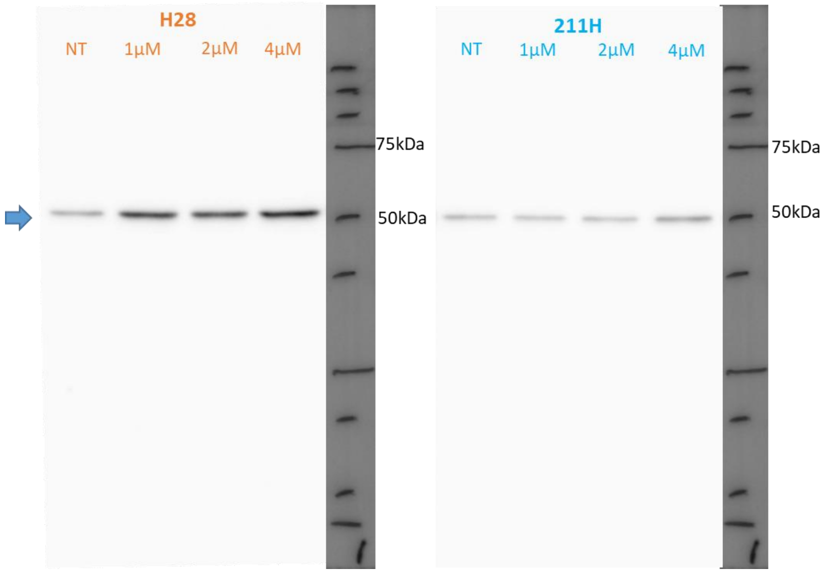

Anti-p53 Ab

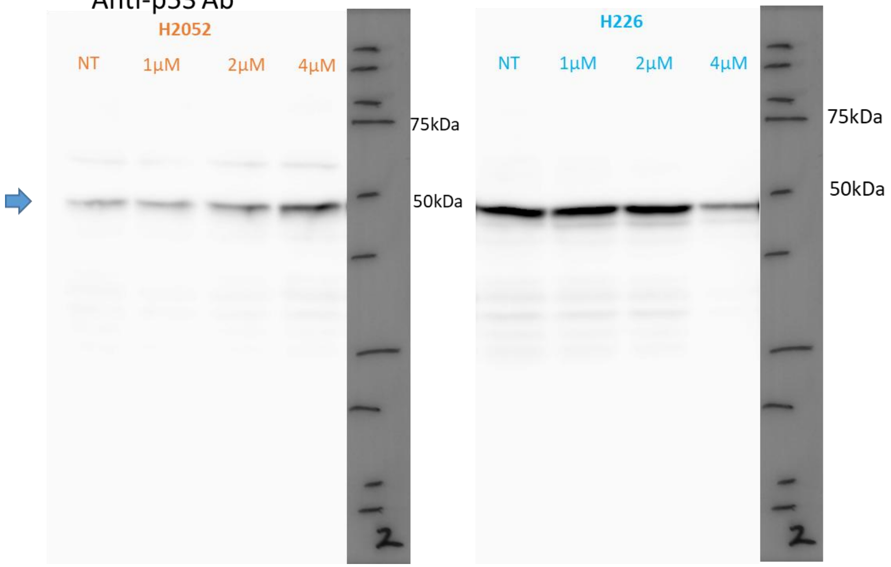

Anti-p53 Ab

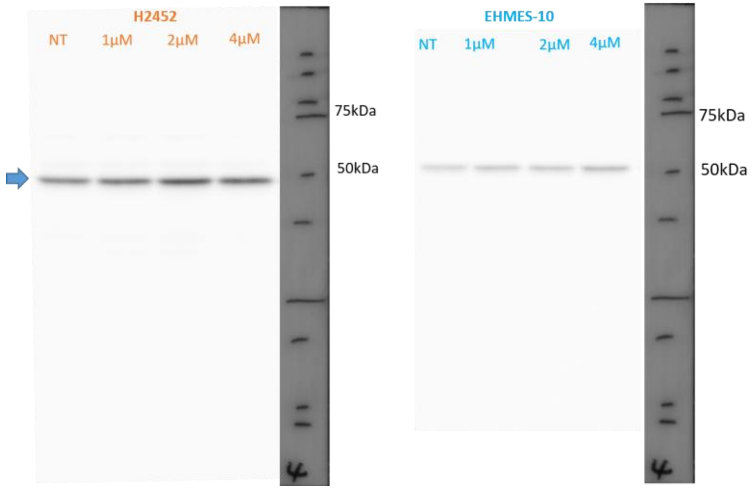

Anti-p53 Ab

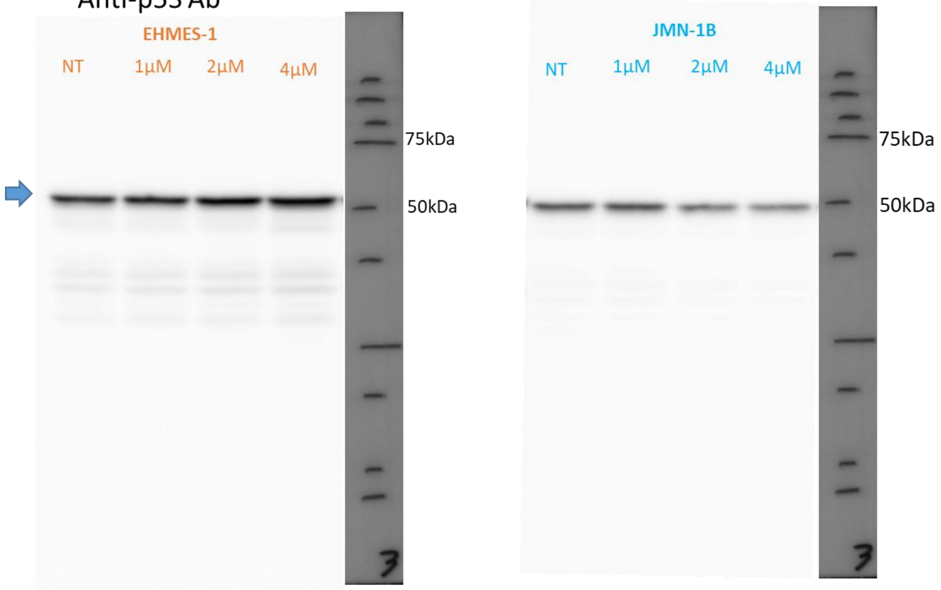

Supplement: Supplementary file 7 [file pone.0361362.s006.zip › 13 Supple Fig 5 (Figure 2B p53) Revised.pdf]
