# Supplementary material for: Correction: Strategic selection of MDM2 inhibitors enhances the efficacy of FAK inhibition in mesothelioma based on TP53 genotype
Source: PLoS One. 2026 Oct 26;21(10):e0361362. doi: 10.1371/journal.pone.0361362 (PMC13704855; doi:10.1371/journal.pone.0361362)

H28 (Nei san's data Exp 4-2)			
Defactinib濃度	%AV	%SE	IC50
0	100.00	0.00	4.809
0.5	97.12	2.20	
2	78.66	0.98	
4	53.90	0.96	
8	22.18	0.66	

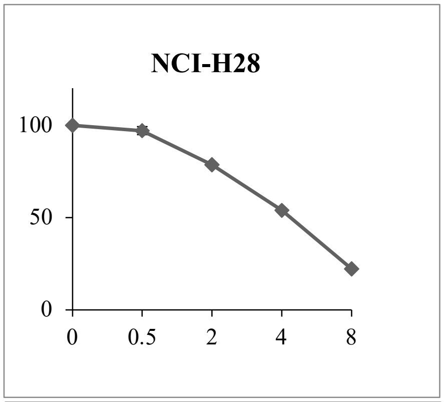

211H (Nei's data Exp 5)

Defactinib濃度	%AV	%SE	IC50
0	100.00	2.11	3.762
0.5	100.00	3.91	
2	91.73	1.81	
4	41.72	2.61	
8	13.30	1.12	

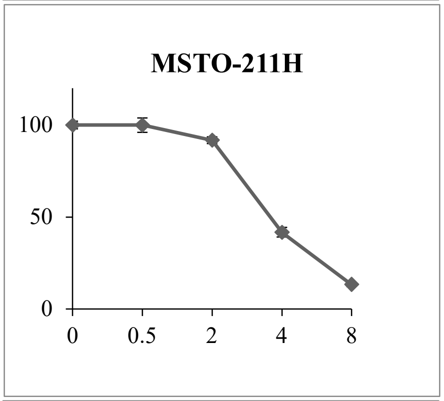

H226 (Nei san's data Exp 22)			
Defactinib濃度	%AV	%SE	IC50
0	100.00	5.34	2.054
0.5	90.41	0.84	
2	47.95	0.55	
4	13.99	0.16	
8	8.58	0.12	

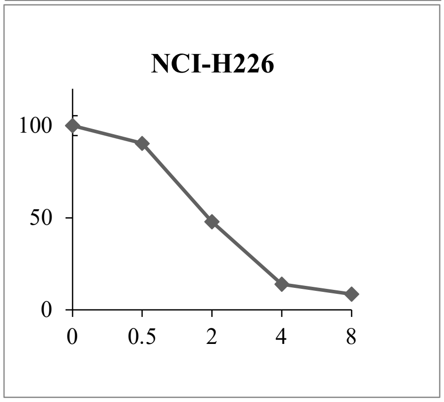

H2052 (Nei san's data Exp 7)			
Defactinib濃度	%AV	%SE	IC50
0	100.00	2.39	4.788
0.5	90.53	4.11	
2	79.47	4.01	
4	55.58	0.09	
8	20.39	0.63	

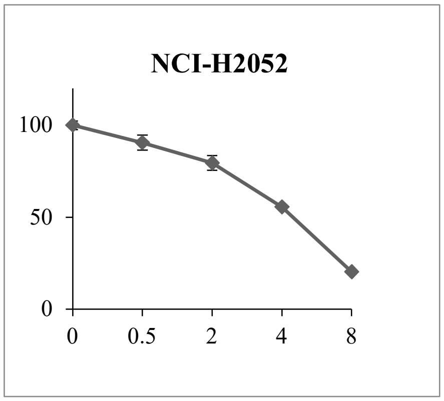

JMN1B (Nei san's data Exp 4-2)			
Defactinib濃度	%AV	%SE	IC50
0	100.00	0.00	5.083
0.5	100.47	2.14	
2	82.97	3.13	

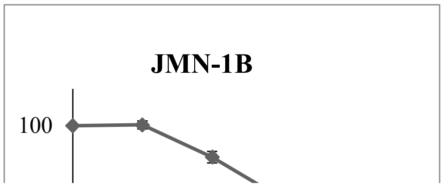

4	60.47	0.79	
8	20.09	0.58	

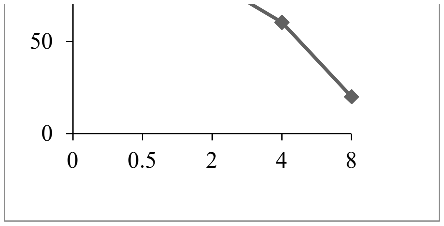

EHMES-1 (Nei san's data Exp 10)

Defactinib濃度	%AV	%SE	IC50
0	100.00	12.55	
0.5	87.77	6.34	
2	43.15	1.05	1.975
4	17.95	1.00	
8	6.86	0.91	

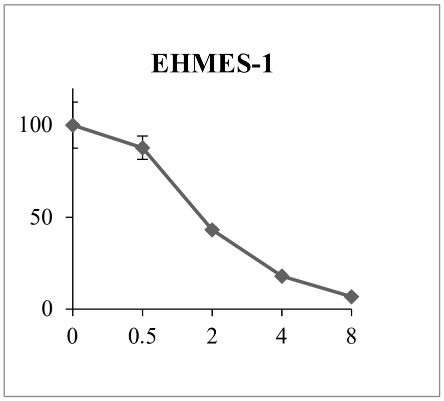

EHMES-10 (Nei san's data Exp 50)

Defactinib濃度	%AV	%SE	IC50
0	100.00	3.72	
0.5	94.93	0.35	
4	72.96	4.36	7.9
8	47.40	3.44	
16	14.80	1.48	

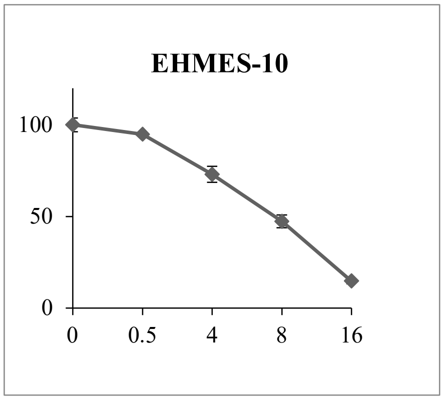

H2452 (Nei san's data Exp 4-2)

Defactinib濃度	%AV	%SE	IC50
0	100.00	0.00	
0.5	91.26	2.88	
2	69.83	1.20	7.46
8	44.11	0.66	
16	15.01	0.57	

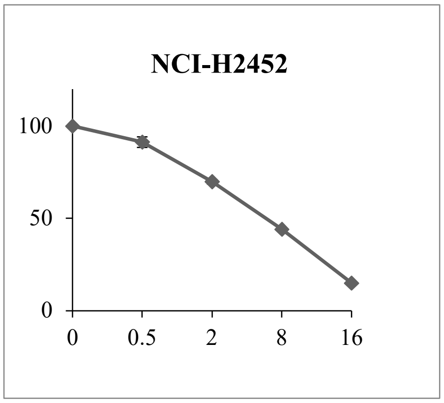

Met5A (Nei san's data Exp 4-2)

Defactinib濃度	%AV	%SE	IC50
0	100.00	0.00	
0.5	98.34	4.02	
2	75.74	3.94	3.559
4	38.58	1.51	
8	17.94	0.32	

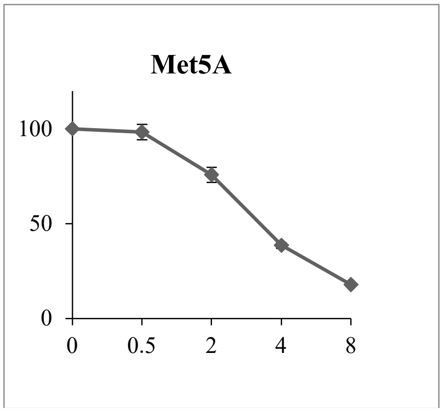

Supplement: Supplementary file 9 [file pone.0361362.s010.zip › 20 Figure 2A Summary.pdf]
