# Supplementary material for: Correction: Strategic selection of MDM2 inhibitors enhances the efficacy of FAK inhibition in mesothelioma based on TP53 genotype
Source: PLoS One. 2026 Oct 26;21(10):e0361362. doi: 10.1371/journal.pone.0361362 (PMC13704855; doi:10.1371/journal.pone.0361362)

H28 (Nei san's data Exp 21)

Nutlin濃度	%AV	%SE	IC50
0	100.00	2.10	
1	96.91	0.18	
2	85.17	1.94	6.717
4	70.07	1.09	
8	39.22	0.39	

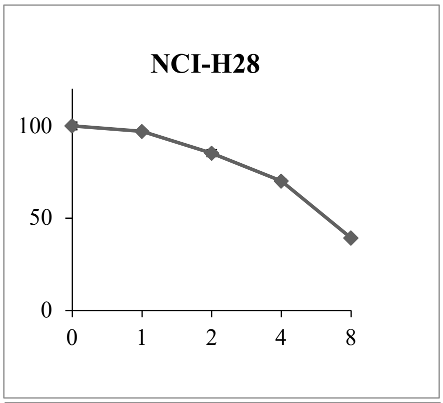

211H（Kozono san's data Exp 9）

Nutlin濃度	%AV	%SE	IC50
0	100.00	1.82	
1	80.96	2.00	
2	52.63	0.68	2.346
4	20.60	0.86	
8	5.88	0.20	

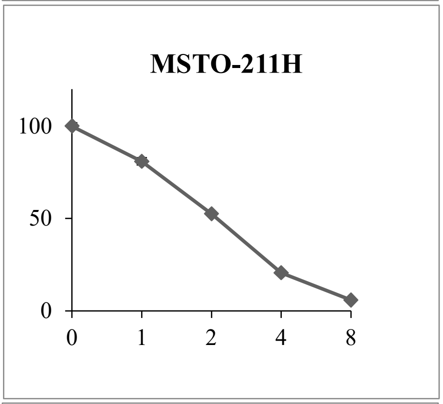

H226 (Nei san's data Exp 22)

Nutlin濃度	%AV	%SE	IC50
0	100.00	2.89	
0.5	91.36	1.27	
1	79.24	1.09	2.908
2	55.26	1.67	
4	36.31	0.58	

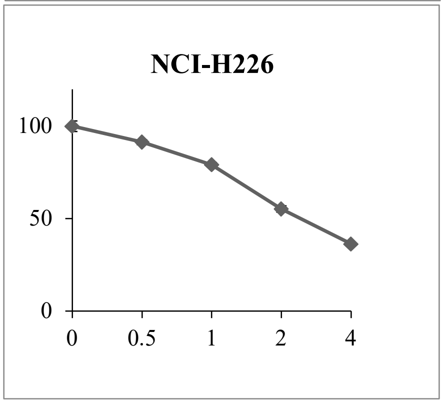

H2052 (Nei san's data Exp 29)

Nutlin濃度	%AV	%SE	IC50
0	100.00	1.14	
0.5	96.30	1.83	
1	79.66	1.25	2.531
2	49.06	0.84	
4	29.19	1.03	

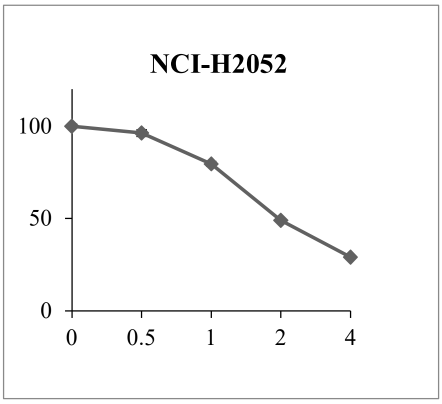

JMN1B (Nei san's data Exp 30)

Nutlin濃度	%AV	%SE	IC50
0	100.00	1.31	
10	88.17	1.81	
20	74.37	1.35	28.91
30	48.54	3.42	
40	19.04	0.88	

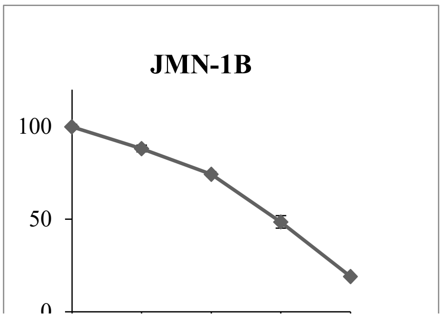

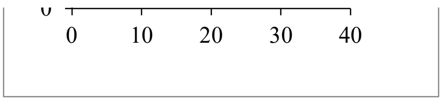

EHMES-1 (Nei san's data Exp 17)

Nutlin濃度	%AV	%SE	IC50
0	100.00	2.73	41.55
20	91.36	1.32	
30	83.08	0.68	
40	57.79	2.12	
60	2.61	0.15	

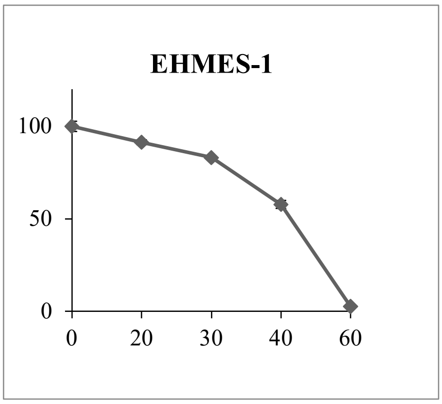

EHMES-10 (Nei san's data Exp 18)

Nutlin濃度	%AV	%SE	IC50
0	100.00	3.17	59.38
20	92.67	0.79	
30	79.04	1.13	
40	63.94	1.27	
60	8.26	1.16	

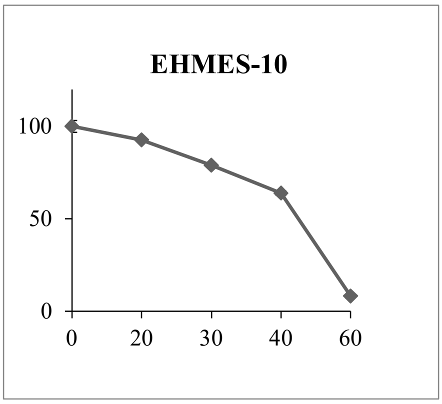

H2452 (Nei san's data Exp 28)

Nutlin濃度	%AV	%SE	IC50
0	100.00	1.97	30.89
10	97.77	1.09	
20	74.17	1.94	
30	47.74	0.52	
40	31.83	1.00	

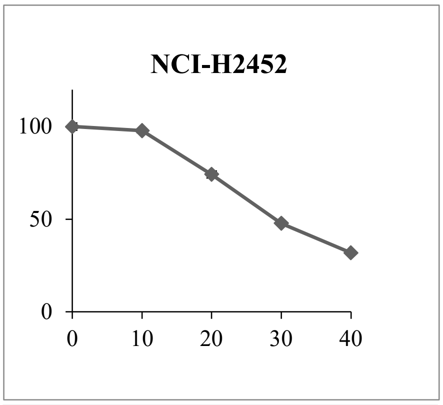

Met5A (Nei san's data Exp 31)

Nutlin濃度	%AV	%SE	IC50
0	100.00	1.27	32.62
10	101.01	1.82	
20	87.54	3.10	
30	63.97	1.06	
40	18.92	1.42	

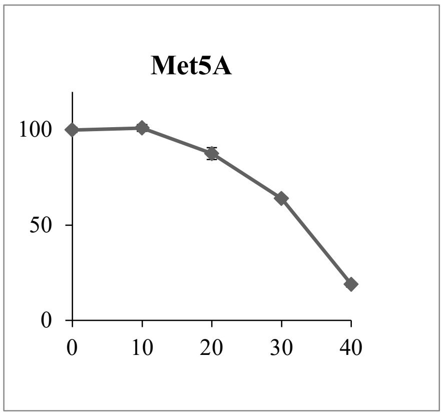

Supplement: Supplementary file 9 [file pone.0361362.s010.zip › 27 Figure 3A Summary.pdf]
