# Supplementary material for: Correction: Strategic selection of MDM2 inhibitors enhances the efficacy of FAK inhibition in mesothelioma based on TP53 genotype
Source: PLoS One. 2026 Oct 26;21(10):e0361362. doi: 10.1371/journal.pone.0361362 (PMC13704855; doi:10.1371/journal.pone.0361362)

H28 (Nei san's data Exp 11-2-4)

Rita濃度	%AV	%SE	IC50
0	100.00	2.82	0.061
0.01	86.40	1.56	
0.03	69.39	4.73	
0.06	44.00	3.02	
0.12	21.99	1.01	

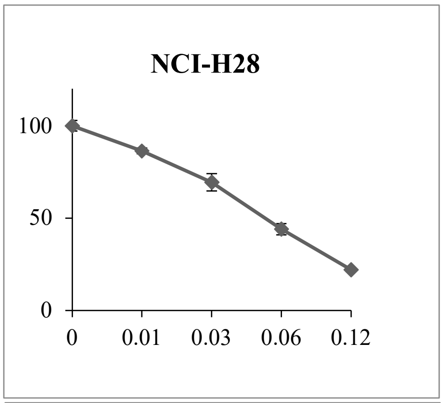

211H (Thao's data Exp 16)

Rita濃度	%AV	%SE	IC50
0	100.00	1.50	1.924
0.5	82.99	2.25	
2	47.78	1.42	
8	28.47	0.79	
32	4.13	0.05	

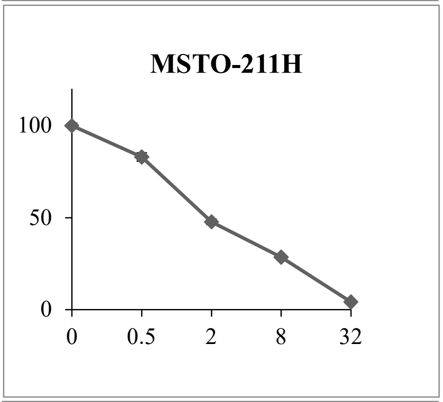

H226 (Nei san's data Exp 11-2-4)

Rita濃度	%AV	%SE	IC50
0	100.00	2.00	0.032
0.01	75.47	3.62	
0.03	49.83	5.75	
0.06	18.88	3.94	
0.12	4.08	4.16	

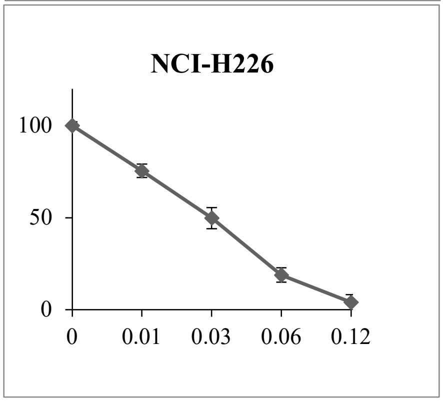

H2052 (Nei san's data Exp 11-2-2)

Rita濃度	%AV	%SE	IC50
0	100.00	1.16	7.984
0.5	104.12	2.60	
2	85.78	3.10	
8	50.22	1.82	
32	20.75	0.52	

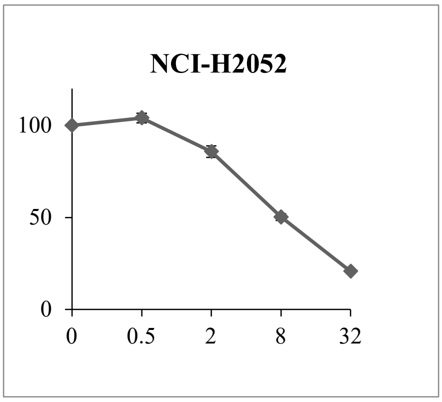

JMN1B (Nei san's data Exp 11-2-1)

Rita濃度	%AV	%SE	IC50
0	100.00	1.49	5.152
0.5	77.99	4.74	
2	56.19	0.64	
8	33.96	1.24	
32	0.43	0.22	

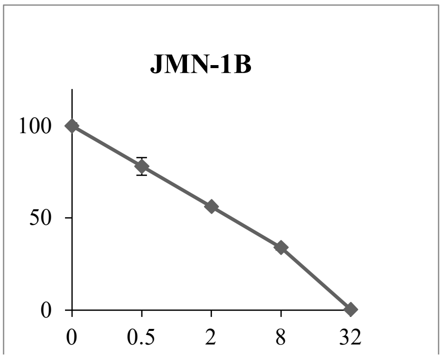

EHMES-1 (Nei san's data Exp 11-2-3)

Rita濃度	%AV	%SE	IC50
0	100.00	14.70	
0.125	75.17	10.27	
2	35.51	5.88	1.534
8	20.76	1.74	
32	6.46	0.48	

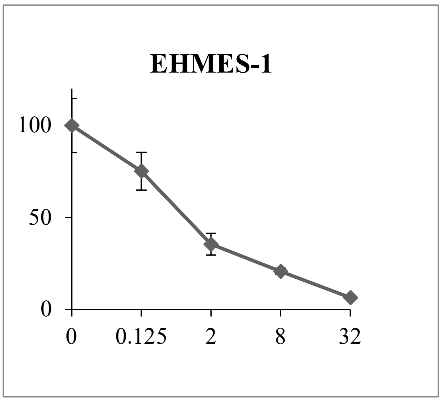

EHMES-10 (Nei san's data Exp 11-2-3)

Rita濃度	%AV	%SE	IC50
0	100.00	4.59	
0.5	102.67	0.09	
2	99.89	0.20	>32
8	88.81	0.24	
32	59.26	0.32	

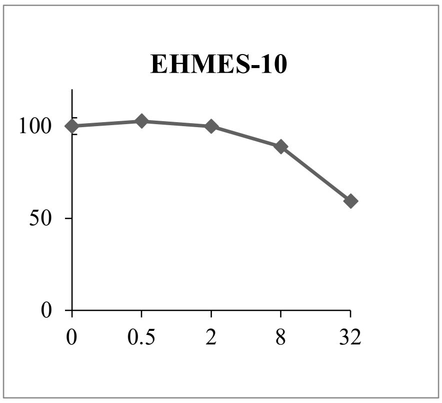

H2452 (Nei san's data Exp 11-2-4)

Rita濃度	%AV	%SE	IC50
0	100.00	3.09	
0.002	72.37	2.59	
0.01	42.04	2.62	0.009
0.03	20.06	0.61	
0.06	10.19	0.85	

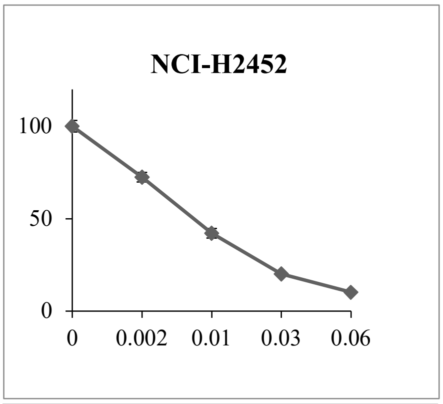

Met5A (Nei san's data Exp 11-2-4)

Rita濃度	%AV	%SE	IC50
0	100.00	0.90	
0.002	90.36	2.11	
0.01	51.20	0.90	0.019
0.03	29.96	0.94	
0.06	15.51	0.49	

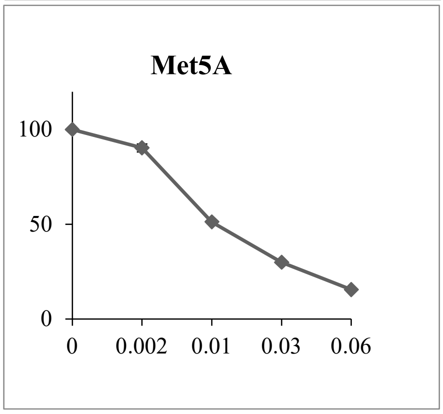

Supplement: Supplementary file 9 [file pone.0361362.s010.zip › 37 Figure 4A Summary.pdf]
