# Supplementary material for: Correction: Strategic selection of MDM2 inhibitors enhances the efficacy of FAK inhibition in mesothelioma based on TP53 genotype
Source: PLoS One. 2026 Oct 26;21(10):e0361362. doi: 10.1371/journal.pone.0361362 (PMC13704855; doi:10.1371/journal.pone.0361362)

H28 (Nei san's data Exp 21)

Nutlin-3a	%	SE%
0	100.00	2.10
1	96.91	0.18
2	85.17	1.94
4	70.07	1.09
6	53.32	0.27
8	39.22	0.39

1 µM Defa	%	SE%
0	87.43	1.30
1	82.67	0.29
2	73.08	0.40
4	54.30	2.45
6	43.25	0.59
8	35.94	0.84

CI For experimental values

nutlin-3a (uM)	defactinib (uM)	Fa	CI
1	1	0.173251	0.953
2	1	0.269155	0.92
3	1	0.372563	0.887
4	1	0.457	0.888
5	1	0.513291	0.928
6	1	0.567497	0.951
7	1	0.629209	0.934
8	1	0.640571	1.023

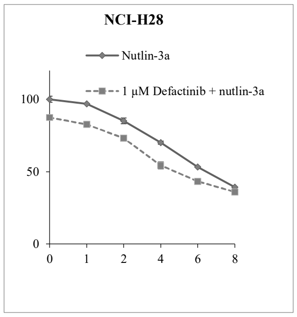

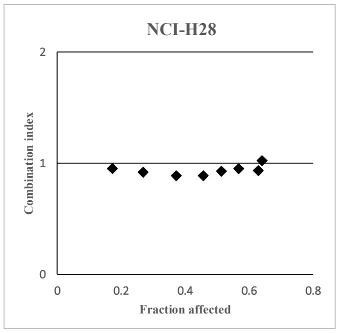

H226 (Nei san's data Exp 22)

Nutlin-3a	%	SE%
0	100.00	2.89
0.5	91.36	1.27
1	79.24	1.09
2	55.26	1.67
3	39.17	1.37
4	36.31	0.58

1.5 µM De	%	SE%
0	83.04	2.73
0.5	75.76	2.09
1	60.59	3.77
2	39.91	1.51
3	31.67	1.10
4	29.38	0.90

CI For experimental values

nutlin-3a (uM)	defactinib (uM)	Fa	CI
0.5	1.5	0.242363	0.917
1	1.5	0.394094	0.721
1.5	1.5	0.50891	0.694
2	1.5	0.600942	0.682
2.5	1.5	0.657714	0.706
3	1.5	0.683299	0.776
3.5	1.5	0.719577	0.798
4	1.5	0.706212	0.951

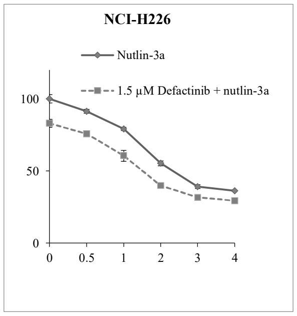

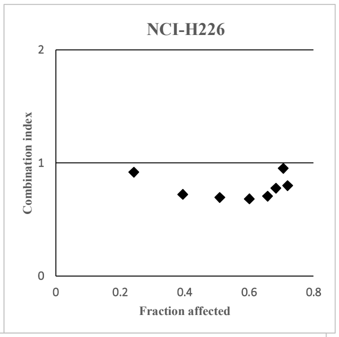

H2052 (Nei san's data Exp 19)

Nutlin-3a	%	SE%
0	100.17	2.72
0.25	96.30	1.23
0.5	93.77	1.13
1	77.86	1.15
2	56.90	0.86
3	45.82	1.54

2 µM Defa	%	SE%
0	88.94	0.84
0.25	80.30	0.97
0.5	74.87	3.87
1	61.99	2.65
2	48.75	1.14
3	39.92	0.29

CI For experimental values

nutlin-3a (uM)	defactinib (uM)	Fa	CI
0.25	2	0.196978	1.101
0.5	2	0.251304	1.094
0.75	2	0.329016	1.003
1	2	0.380104	0.997
1.5	2	0.462853	1.007
2	2	0.512502	1.069
2.5	2	0.565569	1.088
3	2	0.600827	1.134

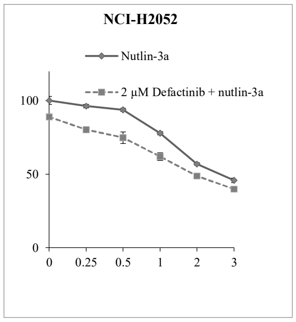

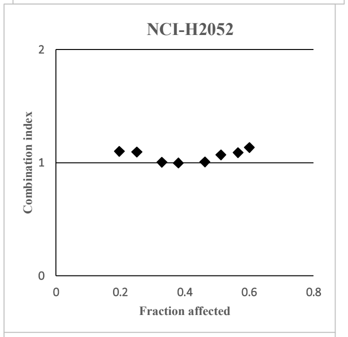

EHMES-10 (Nei san's data Exp 18)

Nutlin-3a	%	SE%
0	100.00	3.17
10	99.73	0.56
20	92.67	0.79
30	79.04	1.13
40	63.94	1.27
50	41.19	1.70

1 µM Defa	%	SE%
0	83.73	1.97
10	79.14	1.87
20	70.15	2.46
30	61.77	1.12
40	42.91	1.90
50	24.58	0.40

CI For experimental values

nutlin-3a (uM)	defactinib (uM)	Fa	CI
10	1	0.208649	1.076
20	1	0.298502	0.96
30	1	0.382299	1.021
40	1	0.570924	0.96
45	1	0.668013	0.942
50	1	0.754165	0.926
55	1	0.839643	0.882
60	1	0.960626	0.652

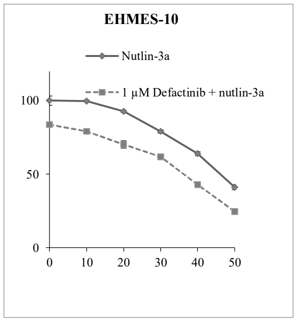

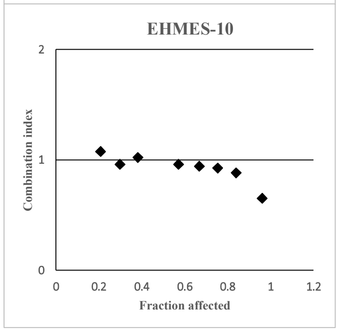

H2452 (Nei san's data Exp 16)

Nutlin-3a	%	SE%
0	90.28	1.96
10	85.72	4.33
15	74.18	0.99
20	67.83	1.45
30	46.46	1.01
40	32.17	0.16

0.5 µM De	%	SE%
0	83.23	2.58
10	70.54	1.44
15	61.13	0.87
20	54.92	1.44
30	38.28	1.03
40	30.15	0.65

CI For experimental values

nutlin-3a (uM)	defactinib (uM)	Fa	CI
5	0.5	0.245699	1.424
10	0.5	0.294638	1.189
15	0.5	0.388675	0.903
20	0.5	0.45081	0.898
25	0.5	0.541841	0.82
30	0.5	0.617171	0.778
35	0.5	0.634709	0.857
40	0.5	0.698513	0.8

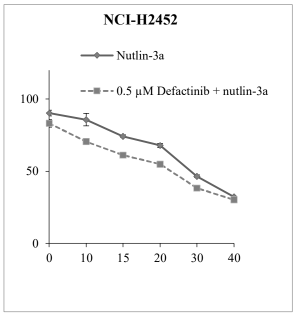

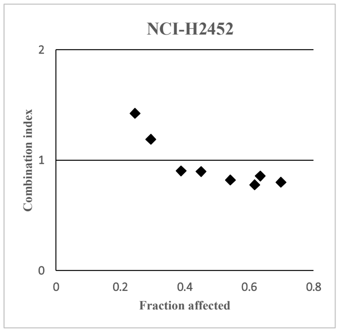

EHMES1 (Nei san's data Exp 17)

Nutlin-3a	%	SE%
0	100.00	2.82
20	91.37	1.12
30	82.13	0.49
40	56.48	2.07
50	24.86	0.35
60	2.59	0.12

1 µM Defa	%	SE%
0	87.23	1.02
20	70.55	3.90
30	65.00	0.68
40	39.69	1.80
50	16.33	1.74
60	1.98	0.18

CI For experimental values

nutlin-3a (uM)	defactinib (uM)	Fa	CI
10	1	0.19082	1.283
20	1	0.294482	1.168
30	1	0.350009	1.357
40	1	0.60306	1.114

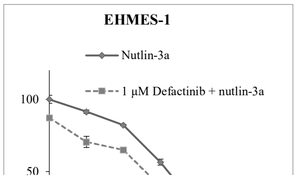

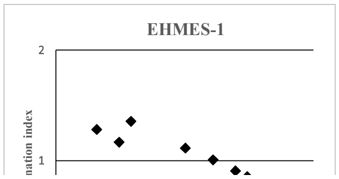

45	1	0.732508	1.009
50	1	0.836686	0.908
55	1	0.891697	0.856
60	1	0.98023	0.532

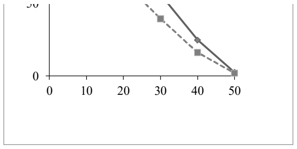

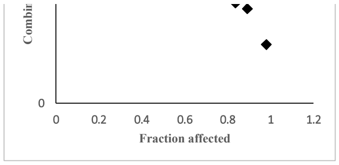

JMN1B (Nei san's data Exp 23)

Nutlin-3a	%	SE%
0	100.00	3.68
10	87.61	4.53
15	79.99	4.77
20	71.92	1.04
30	48.21	1.46
40	20.89	0.45

1 µM Defa	%	SE%
0	84.14	2.12
10	72.43	5.56
15	62.51	2.84
20	50.95	1.44
30	35.94	1.23
40	16.51	0.55

CI For experimental values

nutlin-3a (uM)	defactinib (uM)	Fa	CI
10	1	0.27566	1.235
15	1	0.374898	1.156
20	1	0.490471	1.071
25	1	0.492921	1.255
30	1	0.640621	1.067
35	1	0.732235	0.99
40	1	0.834876	0.838
45	1	0.877484	0.798

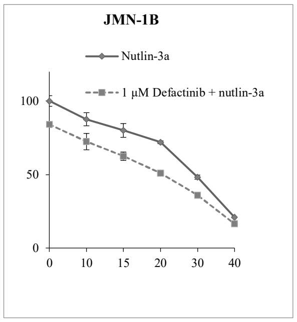

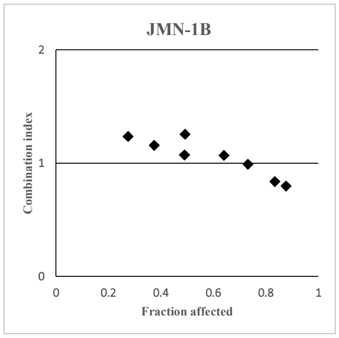

Supplement: Supplementary file 9 [file pone.0361362.s010.zip › 43 Figure 5 Summary.pdf]
