# Supplementary material for: Correction: Strategic selection of MDM2 inhibitors enhances the efficacy of FAK inhibition in mesothelioma based on TP53 genotype
Source: PLoS One. 2026 Oct 26;21(10):e0361362. doi: 10.1371/journal.pone.0361362 (PMC13704855; doi:10.1371/journal.pone.0361362)

H28 (Nei san's data Exp 12)

RITA	%	SE%
0	100.00	1.57
0.01	96.56	2.49
0.02	89.19	1.74
0.04	58.94	2.86
0.08	35.13	0.36
0.13	23.61	0.86

1 μM Defactinib	%	SE%
0	88.20	1.91
0.01	84.29	5.07
0.02	74.45	1.90
0.04	44.68	1.04
0.08	24.45	0.24
0.13	15.78	1.16

CI For experimental values

RITA (uM)	Defactinil (uM)	Fa	CI
0.005	1	0.127899	1.007
0.01	1	0.157129	1.059
0.02	1	0.255524	1.004
0.04	1	0.553177	0.756
0.06	1	0.681488	0.779
0.08	1	0.755455	0.821
0.1	1	0.809387	0.845
0.125	1	0.842185	0.92

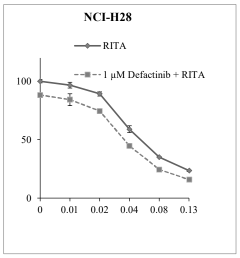

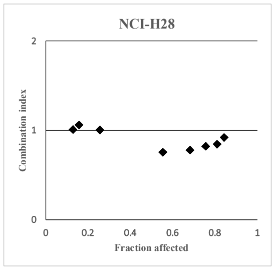

211H

RITA	%	SE%

2μM Defactinib	%	SE%

H2052 (Nei san's data Exp 8)

RITA	%	SE%
0	98.94	2.35
0.5	91.61	1.96
2	76.90	1.50
4	61.88	1.69
8	48.56	1.10
16	41.36	1.14

0.5μM Defactinil	%	SE%
0	95.60	3.47
0.5	82.99	3.85
2	65.08	2.17
4	47.62	1.98
8	36.84	2.56
16	34.14	0.52

CI For experimental values

RITA (uM)	Defactinil (uM)	Fa	CI
0.125	0.5	0.126112	1.136
0.25	0.5	0.139117	1.152
0.5	0.5	0.170089	1.094
1	0.5	0.210815	1.106
2	0.5	0.349247	0.697
4	0.5	0.523785	0.454
8	0.5	0.631588	0.449
16	0.5	0.658624	0.718

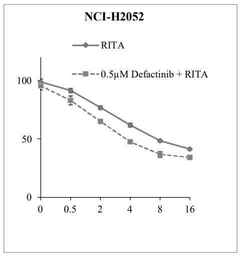

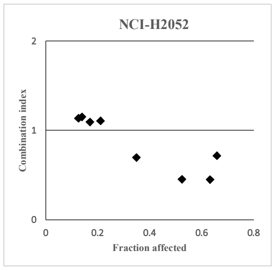

EHMES-1 (Nei san's data Exp 15)

RITA	%	SE%
0	100.00	4.99
0.13	86.72	2.46
0.25	75.07	0.51
0.5	63.18	2.27
2	37.49	1.90
8	19.30	0.43

0.5μM Defactinil	%	SE%
0	89.85	3.28
0.13	75.97	1.17
0.25	64.34	0.70
0.5	52.11	1.76
2	31.46	1.24
8	20.01	0.22

I For experimental values

RITA (uM)	Defactinil (uM)	Fa	CI
0.125	0.5	0.303704	0.909
0.25	0.5	0.329167	1.068
0.5	0.5	0.456944	0.891
1	0.5	0.605556	0.72
2	0.5	0.716204	0.67
4	0.5	0.756019	0.904
8	0.5	0.798148	1.221
16	0.5	0.842593	1.58

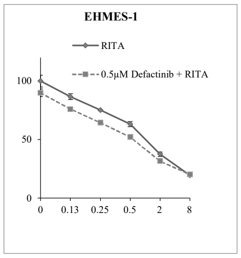

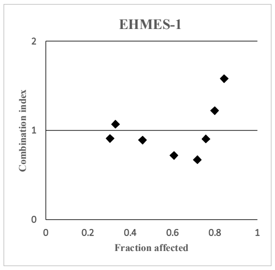

JMN1B (Nei san's data Exp 16)

RITA	%	SE%
0	97.23	1.80
0.25	97.14	3.29
0.50	94.69	1.83
2.00	74.48	2.27
4.00	56.57	1.59
8.00	45.33	2.05

1μM Defactinib -	%	SE%
0	83.24	3.21
0.25	81.99	1.29
0.5	75.70	0.61
2	52.38	0.77
4	41.52	1.46
8	27.22	0.78

CI For experimental values

RITA (uM)	defactinit (uM)	Fa	CI
0.125	1	0.163122	1.111
0.25	1	0.180112	1.102
0.5	1	0.242961	0.953
1	1	0.319116	0.888
2	1	0.476179	0.724
4	1	0.584769	0.771
8	1	0.727813	0.752
16	1	0.863015	0.645

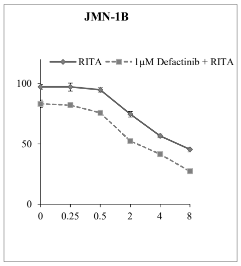

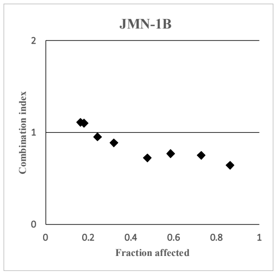

Supplement: Supplementary file 9 [file pone.0361362.s010.zip › 61 Figure 6 Summary.pdf]
