# Supplementary material for: Correction: Strategic selection of MDM2 inhibitors enhances the efficacy of FAK inhibition in mesothelioma based on TP53 genotype
Source: PLoS One. 2026 Oct 26;21(10):e0361362. doi: 10.1371/journal.pone.0361362 (PMC13704855; doi:10.1371/journal.pone.0361362)
Supplement: Supplementary file 9 [file pone.0361362.s010.zip › Explanation of underlying data files.docx]

**Figure 2A**

We have summary data in “20 Figure 2A Summary”, which shows the data for Figure 2A preparation. The following data from 21 Figure 2A No1 to 26 Figure 2A No6 are individual data.

(1) H28, H2452, JMN-1B and Met-5A

Please look at Excel data (21 Figure 2A No1) at 2.5h (H28) and 3h (H2452, JMN-1B and Met-5A) with marked blue. Clicking the graph shows the data.

(2) MSTO-211H

Please look at Excel (22 Figure 2A No2) at 3h (selected) data with marked blue.

(3) H2052

Please look at Excel (23 Figure 2A No3) at 3h (selected) data with marked blue.

(4) H226

Please look at Excel (24 Figure 2A No4) at 3h data with marked blue.

(5) EHMES-10

Please look at Excel (25 Figure 2A No5) at 4h まとめ data with marked blue. Clicking the graph shows the data.

(6) EHMES-1

Please look at Excel (26 Figure 2A No6) at 3h (selected) data (left side column). with marked blue. Please pay attention to the difference of defectinib concentrations.

**Figure 3A**

We have summary data in “27 Figure 3A Summary”, which shows the data for Figure 3A preparation. The following data from 28 Figure 3A No1 to 36 Figure 3A No9 are individual data.

(1) H28

Please look at Excel (28 Figure 3A No1) at 3h (selected) with marked blue.

(2) MSTO-211H

Please look at Excel (29 Figure 3A No2) at sheet1 with marked blue.

(3) H2052

Please look at Excel (30 Figure 3A No3) at 3h with marked blue.

(4) H226

Please look at Excel (31 Figure 3A No4) at 3h (selected) with marked blue.

(5) EHMES-10

Please look at Excel (32 Figure 3A No5) at 2.5h (selected) with marked blue.

(6) H2452

Please look at Excel (33 Figure 3A No6) at 3h (selected) with marked blue.

(7) EHMES-1

Please look at Excel (34 Figure 3A No7) at 3h with marked blue.

(8) JMN-1B

Please look at Excel (35 Figure 3A No8) at 2.5h (selected) with marked blue.

(9) Met-5A

Please look at Excel (36 Figure 3A No9) at 3h with marked blue.

**Figure 4A**

We have summary data in “37 Figure 4A Summary”. which shows the data for Figure 4A preparation. The following data from 38 Figure 4A No1 to 42 Figure 4A No5 are individual data.

(1) H28, H226, H2452 and Met-5A

Please look at Excel (38 Figure 4A No1) at 2.5 h data (for H28) and 3h data (H226, H2452 and Met-5a) with marked blue. Clicking the graph shows the data.

(2) MSTO-211H

Please look at Excel (39 Figure 4A No2 shown as 211H) at 211H (Rita only- Nut only) with marked blue.

(3) H2052

Please look at Excel (40 Figure 4A No3) at 2.5h with marked blue. Clicking the graph shows the data.

(4) EHMES-10 and EHMES-1

Please look at Excel (41 Figure 4A No4) at EXP3 3H with marked blue. Clicking the graph showed the data.

(5) JMN-1B

Please look at Excel (42 Figure 4A No5) at 3h with marked blue. Clicking the graph showed the data.

**Figure 5A and 5B**

We have summary data in “43 Figure 5 Summary” which contained the data except MSTO-211H. This summary shows the data for Figure 5A and 5B preparation. The following data from 44 Figure 5A No1 to 60 Figure 5B No8 are individual data.

(1) H28

Please look at Excel (44 Figure 5A No1) at 3h (selected) with marked blue. As for CI values, please look at 45 Figure 5B No1 (PNG file and text file).

(2) MSTO-211H

Please look at Excel (46 Figure 5A No2 shown as 211H) at 6h まとめ with marked blue and red characters. As for CI values, please look at 47 Figure 5B No2 (PNG file and text file). We also have a summary of 211H data (48 Figure 5A No2 Summary) at 211H (Nut+Def) with marked blue.

(3) H2052

Please look at Excel (49 Figure 5A No3) at 3h (selected) with marked blue. As for CI values, please look at 50 Figure 5B No3 (PNG file and text file).

(4) H226

Please look at Excel (51 Figure 5A No4) at 3h (selected) with marked blue. As for CI values, please look at 52 Figure 5B No4 (PNG file and text file).

(5) H2452

Please look at Excel (53 Figure 5A No5) at 3h (selected) with marked blue. As for CI values, please look at 54 Figure 5B No5 (PNG file and text file). In this figure, the Def concentration is 0.5 μM. We are sorry for this mistake.

(6) EHMES-10

Please look at Excel (55 Figure 5A No6) at 2.5h (selected) with marked blue. As for CI values, please look at 56 Figure 5B No6 (PNG file and text file).

(7) EHMES-1

Please look at Excel (57 Figure 5A No7) at 2.5h (selected) with marked blue. As for CI values, please look at 58 Figure 5B No7 (PNG file and text file). In this figure, nutrin-3a concentrations in horizontal X-axis should be 0, 20, 30, 40, 50 and 60 (μM). We are sorry for this mistake.

(8) JMN-1B

Please look at Excel (59 Figure 5A No8) at 3h (selected) with marked blue. As for CI values, please look at 60 Figure 5B No8 (PNG file and text file).

**Figure 6A and 6B**

We have summary data in “61 Figure 6 Summary” which contained H28, H2052, EHMES-1 and JMN-1B data. This summary shows the data for Figure 6A and 6B preparation. The other cells have summary data with Figure 6A No xx Summary below. The following data from 62 Figure 6A No1 to 81 Figure 6B No8 are individual data.

(1) H28

Please look at Excel (62 Figure 6A No1) at 3h (selected) with marked blue. As for CI values, please look at 63 Figure 6B No1 (PNG file and text file).

(2) MSTO-211H

Please look at Excel (64 Figure 6A No2) at 6hまとめwith marked blue and red characters (clicking the graph showed the data), and Excel of 65 Figure 6A No2 Summary) (211H RITA+Def) with marked blue. This summary shows the data for Figure 6A and 6B preparation. As for CI values, please look at 66 Figure 6B No2 (PNG file and text file). In this figure, the RITA concentration is 1.5 μM. We are sorry for this mistake.

(3) H2052

Please look at Excel (67 Figure 6A No3) at 3h (selected) with marked blue. As for CI values, please look at 68 Figure 6B No3 (PNG file and text file). In this figure, the RITA concentration is 0.5 μM. We are sorry for this mistake.

(4) H226

Please look at Excel (69 Figure 6A No4) at 3.5hまとめwith marked blue and red characters (clicking the graph showed the data), and Excel of 70 Figure 6A No4 Summary (H226 Rita+Def) with marked blue. This summary shows the data for Figure 6A and 6B preparation. As for CI values, please look at 71 Figure 6B No4 (PNG file and text file). In this figure, the RITA concentration is 4 μM. We are sorry for this mistake.

(5) H2452

Please look at Excel (72 Figure 6A No5) at 4hまとめ with marked blue and red characters (clicking the graph showed the data), and Excel of 73 Figure 6A No5 Summary (H2452 Rita+Def). This summary shows the data for Figure 6A and 6B preparation. As for CI values, please look at 74 Figure 6B No5 (PNG file and text file). In this figure, the Def concentration is 3μM. We are sorry for this mistake.

(6) EHMES-10

Please look at Excel (75 Figure 6A No6) at 4hまとめ with marked blue and red characters, and Excel of 76 Figure 6A No6 Summary. (EHMES10 Rita+Def). This summary shows the data for Figure 6A and 6B preparation. As for CI values, please look at 77 Figure 6B No6 (PNG file and text file). In this figure, the Def concentration is 1.5μM. We are sorry for this mistake.

(7) EHMES-1

Please look at Excel (78 Figure 6A No7) at 3h with marked blue. As for CI values, please look at 79 Figure 6B No7 (PNG file and text file). In this figure, the Def concentration is 0.5μM. We are sorry for this mistake.

(8) JMN-1B

Please look at Excel (80 Figure 6A No8) at 3h (selected) with marked blue. As for CI values, please look at 81 Figure 6B No8 (PNG file and text file).

**Supplementary Figure 1**

(1) H28 and MSTO-211H

Please look at Excel (82 Supple Figure 1 No1) at Exp63まとめwith marked blue.

(2) H2052 and H226

Please look at Excel (83 Supple Figure 1 No2) at まとめ（H2052, H226）with marked blue.

(3) H2452 and EHMES-10

Please look at Excel (84 Supple Figure 1 No3) at まとめ（EHMES-10, H2452）with marked blue.
